# Supplementary material for: Mapping ticks and tick-borne pathogens in China
Source: Nat Commun. 2021 Feb 17;12:1075. doi: 10.1038/s41467-021-21375-1 (PMC7889899; doi:10.1038/s41467-021-21375-1)
Supplement: Supplementary file 1 — Supplementary Information [file 41467_2021_21375_MOESM1_ESM.pdf]

# Supplementary Information

Mapping ticks and tick-borne pathogens in China

Zhao et al.

## Table of Contents


| Page | Item |
|---|---|
| 4 | Supplementary Notes |
| 7 | Supplementary Fig. 1: The spatial distribution of the 1,134 counties with at least one record of ticks (yellow) from 1950 to 2018, China |
| 8 | Supplementary Fig. 2: The spatial distribution of the tick genus *Ixodes* |
| 9 | Supplementary Fig. 3: The spatial distribution of the tick genus *Haemaphysalis* |
| 10 | Supplementary Fig. 4: The spatial distribution of the tick genus *Amblyomma* |
| 11 | Supplementary Fig. 5: The spatial distribution of the tick genus *Rhipicephalus* |
| 12 | Supplementary Fig. 6: The spatial distribution of the tick genus *Dermacentor* |
| 13 | Supplementary Fig. 7: The spatial distribution of the tick genus *Hyalomma* |
| 14 | Supplementary Fig. 8: The spatial distribution of the tick genus *Anomalohimalaya* |
| 15 | Supplementary Fig. 9: The spatial distribution of the tick genus *Ornithodoros* |
| 16 | Supplementary Fig. 10: The spatial distribution of the tick genus *Argas* |
| 17 | Supplementary Fig. 11: The effects of major predictors (RC≥5%) on the occurrence of *I. persulcatus* in the BRT models |
| 18 | Supplementary Fig. 12: The effects of major predictors (RC≥5%) on the occurrence of *I. sinensis* in BRT models |
| 19 | Supplementary Fig. 13: The effects of major predictors (RC≥5%) on the occurrence of *I. granulatus* in BRT models |
| 20 | Supplementary Fig. 14: The effects of major predictors (RC≥5%) on the occurrence of *I. crenulatus* in BRT models |
| 21 | Supplementary Fig. 15: The effects of major predictors (RC≥5%) on the occurrence of *I. ovatus* in BRT models |
| 22 | Supplementary Fig. 16: The effects of major predictors (RC≥5%) on the occurrence of *Ha. longicornis* in BRT models |
| 23 | Supplementary Fig. 17: The effects of major predictors (RC≥5%) on the occurrence of *Ha. concinna* in BRT models |
| 24 | Supplementary Fig. 18: The effects of major predictors (RC≥5%) on the occurrence of *Ha. japonica* |

| | |
|---|---|
| | in BRT models |
| 25 | Supplementary Fig. 19: The effects of major predictors (RC≥5%) to the occurrence of *Ha. hystricis* in BRT models |
| 26 | Supplementary Fig. 20: The effects of major predictors (RC≥5%) on the occurrence of *R. sanguineus* in BRT models |
| 27 | Supplementary Fig. 21: The effects of major predictors (RC≥5%) on the occurrence of *R. microplus* in BRT models |
| 28 | Supplementary Fig. 22: The effects of major predictors (RC≥5%) on the occurrence of *R. haemaphysaloides* in BRT models |
| 29 | Supplementary Fig. 23: The effects of major predictors (RC≥5%) on the occurrence of *D. nuttalli* in BRT models |
| 30 | Supplementary Fig. 24: The effects of major predictors (RC≥5%) on the occurrence of *D. silvarum* in BRT models |
| 31 | Supplementary Fig. 25: The effects of major predictors (RC≥5%) on the occurrence of *D. daghestanicus* in BRT models |
| 32 | Supplementary Fig. 26: The effects of major predictors (RC≥5%) on the occurrence of *D. marginatus* in BRT models |
| 33 | Supplementary Fig. 27: The effects of major predictors (RC≥5%) on the occurrence of *Hy. scupens* in BRT models |
| 34 | Supplementary Fig. 28: The effects of major predictors (RC≥5%) on the occurrence of *Hy. asiaticum* in BRT models |
| 35 | Supplementary Fig. 29: The effects of major predictors (RC≥5%) on the occurrence of *Ar. persicus* in BRT models |
| 36 | Supplementary Fig. 30: The predicted distributions of the top five *Ixodes* tick species based on the BRT models |
| 37 | Supplementary Fig. 31: The predicted distributions of the top four *Haemaphysalis* tick species based on the BRT models |
| 38 | Supplementary Fig. 32: The predicted distributions of the top three *Rhipicephalus* tick species based on the BRT models |
| 39 | Supplementary Fig. 33: The predicted distributions of the top four *Demacentor* tick species based on the BRT models |
| 40 | Supplementary Fig. 34: The predicted distributions of the top two *Hyalomma* and the top one *Argas* tick species based on the ensemble BRT model |
| 41 | Supplementary Fig. 35: The locations of species or subspecies of spotted fever group rickettsiae detected in ticks during 1950–2018 in China |
| 42 | Supplementary Fig. 36: The locations of species or subspecies of *Anaplasmataceae* detected in ticks during 1950–2018 in China. |
| 43 | Supplementary Fig. 37: The locations of species or subspecies of *Borrelia* detected in ticks during 1950–2018 in China |

| | |
|---|---|
| 44 | Supplementary Fig. 38: The locations of species or subspecies of *Babesia* spp. detected in ticks during 1950–2018 in China |
| 45 | Supplementary Fig. 39: The locations of species or subspecies of *Theileria* detected in ticks during 1950–2018 in China |
| 46 | Supplementary Fig. 40: The locations of other tick-borne bacteria detected in ticks during 1950–2018 in China |
| 47 | Supplementary Fig. 41: The locations of tick-borne viruses detected in ticks during 1950–2018 in China |
| 48 | Supplementary Fig. 42: The effects of major predictors (RC≥5%) on the occurrence of SFTSV in BRT models |
| 49 | Supplementary Fig. 43: The effects of major predictors (RC≥5%) on the occurrence of TBEV in BRT models |
| 50 | Supplementary Fig. 44: The flow diagram of literature review |
| 51 | Supplementary Table 1: BRT-model-estimated mean (standard deviation) relative contributions of top environmental and ecoclimatic factors (RC≥5%) to the spatial distribution of five most prevalent tick species in the *Ixodes* genus |
| 53 | Supplementary Table 2: BRT-model-estimated mean (standard deviation) relative contributions of top environmental and ecoclimatic factors (RC≥5%) to the spatial distribution of four most prevalent tick species in the *Haemaphysalis* genus |
| 55 | Supplementary Table 3: BRT-model-estimated mean (standard deviation) relative contributions of top environmental and ecoclimatic factors (RC≥5%) to the spatial distribution of three most prevalent tick species in the *Rhipicephalus* genus |
| 56 | Supplementary Table 4: BRT-model-estimated mean (standard deviation) relative contributions of major environmental and ecoclimatic factors (RC≥5%) to the spatial distribution of four most prevalent tick species in the *Demacentor* genus |
| 57 | Supplementary Table 5: BRT-model-estimated mean (standard deviation) relative contributions of major environmental and ecoclimatic factors (RC≥5%) to the spatial distribution of two most prevalent tick species in the *Hyalomma* genus and one most prevalent tick specie in the *Argas* genus |
| 58 | Supplementary Table 6: The specific references for all 124 tick species in China from 1950 to 2018 |
| 62 | Supplementary Table 7: The social, environmental and ecoclimatic variables used for ecological modeling in this study |
| 64 | Supplementary References |

# Supplementary Notes

## Supplementary Note 1: The integrated database for tick species and tick-borne pathogens

Through literature review, we found a total of 8,953 references, 3,859 in English and 5,094 in Chinese, which met our search criteria. With consensus of two independent reviewers, 605 publications met our study inclusion criteria and were used for data extraction, of which 324 reported detection of tick-borne agents (Supplementary Fig. 44).

After pooling data from all sources, we obtained an integrated database of 7,344 tick records, and 2,060 tick-borne pathogen records. For tick genus, we assembled 1,943, 1,738, 1,163, 1,069, 1048, 193, 112, 68, and ten occurrence records for *Heamaphysalis*, *Dermacentor*, *Ixodes*, *Rhipicephalus*, *Hyalomma*, *Argas*, *Amblyomma*, *Ornithodoros*, and *Anomalohimalaya*, respectively. Supplementary Data 1 shows the numbers of occurrence counties used in our analyses for each tick species.

## Supplementary Note 2: The locations of each specific agent in ticks in mainland China

We georeferenced and mapped recorded locations with positive detection of 103 tick-borne agents from 54 tick species in China, including 22 *Rickettsia* species, 18 *Anaplasmataceae*, 12 *Borrelia*, 18 *Babesia* spp., seven *Theileria*, 19 viruses, seven bacteria including *Coxiella burnetii, Francisella tularensis*, *Colpodella* spp., *Bartonella* spp., *Alcaligenes faecalis*, *Brucella abortus* and *Brucella melitensis* (Fig. 3). Altogether, 22 species or subspecies of *Rickettsia* were detected from 28 species of ticks, with *R. raoultii*, *R. heilongjiangensis*, and *R. sibirica* being the most common species detected from ticks in mainland China (Supplementary Fig. 35). A total of 18 species or subspecies in the *Anaplasmataceae* were detected from 26 species of ticks,

the distribution of which covers a wide range of forest areas in northeastern, northwestern and northern China, as well as some areas in central and southern China (Supplementary Fig. 36). *Borrelia*, the earliest reported tick borne pathogens in China, had a high frequency of literature reporting. Altogether 12 species of *Borrelia* were detected from 31 tick species, mainly distributed in the forest areas of northeastern, northwestern, and central-southern China. *B. burgdorferi* sensu stricto and *B. garinii* were the two most commonly seen species of *Borrelia*, both of which were detected in a variety of tick species (Supplementary Fig. 37). Eighteen *Babesia* species were detected in 20 tick species, which were mainly distributed in pastoral or semi-pastoral areas of northeastern, northwestern and central China. It is interesting to note that, despite the rich variety of *Babesia* species, their geographic distribution was rather limited. Except for *Ba. microti*, most *Babesia* species were reported only in local areas (Supplementary Fig. 38). Seven *Theileria* species were detected from 13 tick species mainly in northwestern and central-eastern China, and *T. annulate* appeared to the most common species. Two other species, *B. persica* and *B. latyshevyi*, were only detected from the tick species of *O. papillipes* and *O. tartakovskyi*, spatially confined to the Xinjiang Uygur Autonomous Region in northwestern China (Supplementary Fig. 39). Seven bacteria were detected from 18 tick species. *F. tularensis* and *C. bumetii*, were detected from five and 14 tick species respectively, which were distributed in forest patches across northeastern and northwestern China (Supplementary Fig. 40). Nineteen viruses were detected from 20 tick species. TBEV was mainly detected from five tick species in northeastern and northwestern China, with *I. persulcatus* as the dominant host tick species. CCHFV was exclusively detected from *Hy. asiaticum* in the Xinjiang Uygur Autonomous Region of northwestern China. SFTSV was detected from four tick species (*Ha. longicornis*, *Ha.*

*concinna*, *R. sanguineus* and *R. microplus*) in its ecological niches in eastern and central China (Supplementary Fig. 41).

## Supplementary Figures:

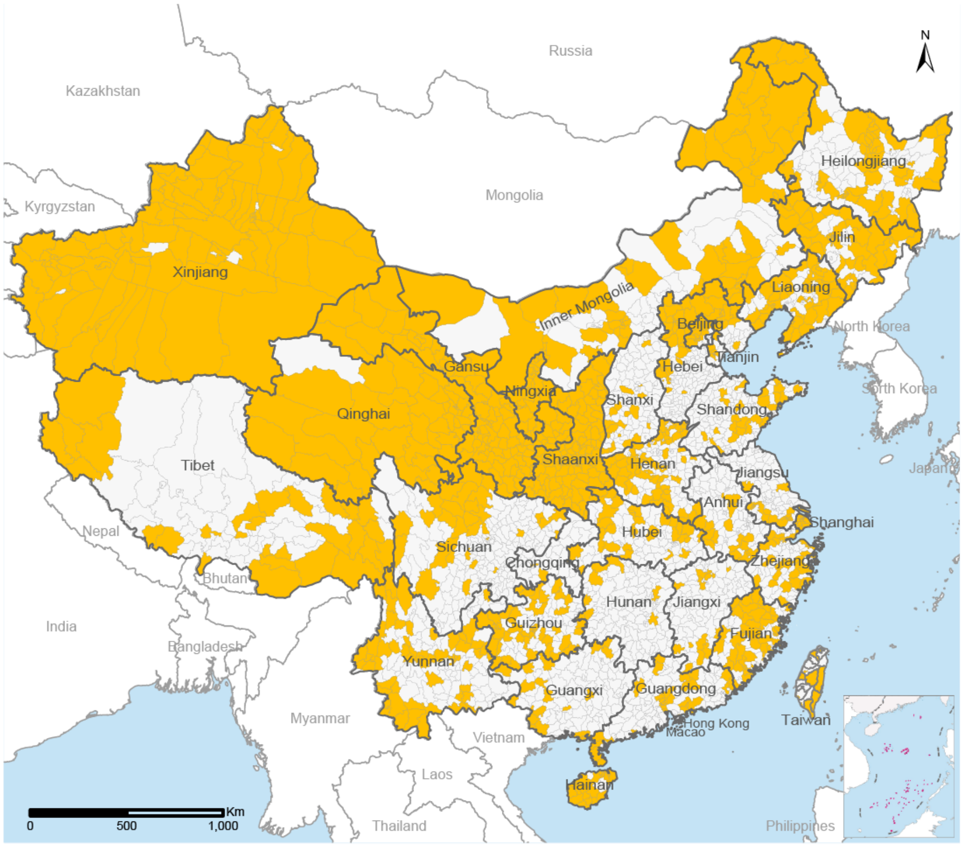


**Supplementary Fig. 1 The spatial distribution of the 1,134 counties with at least one record of ticks (yellow) from 1950 to 2018, China.** Source data are provided as a Source Data file.

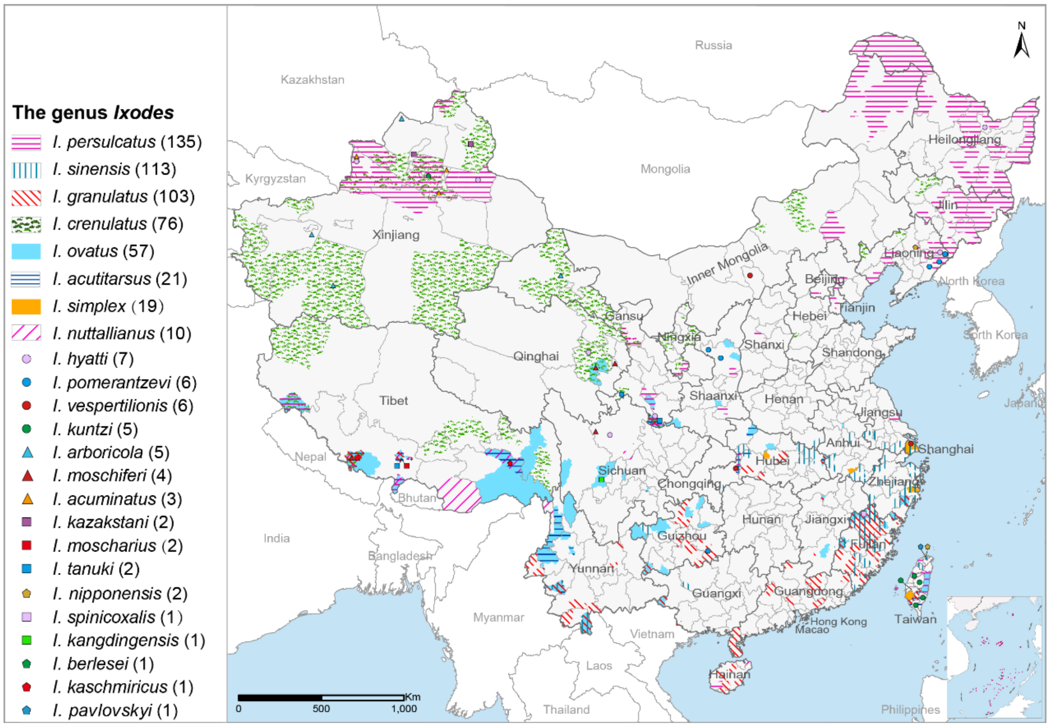


**Supplementary Fig. 2 The spatial distribution of the tick genus *Ixodes* recorded at the county level from 1950 to 2018 in China.** Source data are provided as a Source Data file.

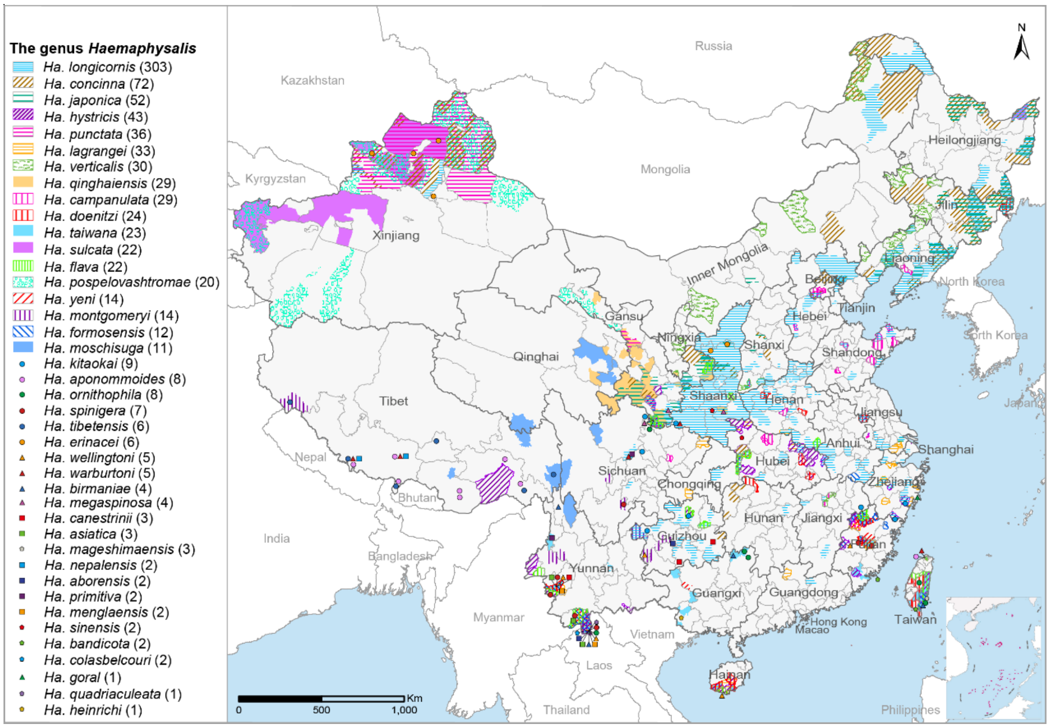


**Supplementary Fig. 3 The spatial distribution of the tick genus *Haemaphysalis* recorded at the county level from 1950 to 2018 in China.** Source data are provided as a Source Data file.

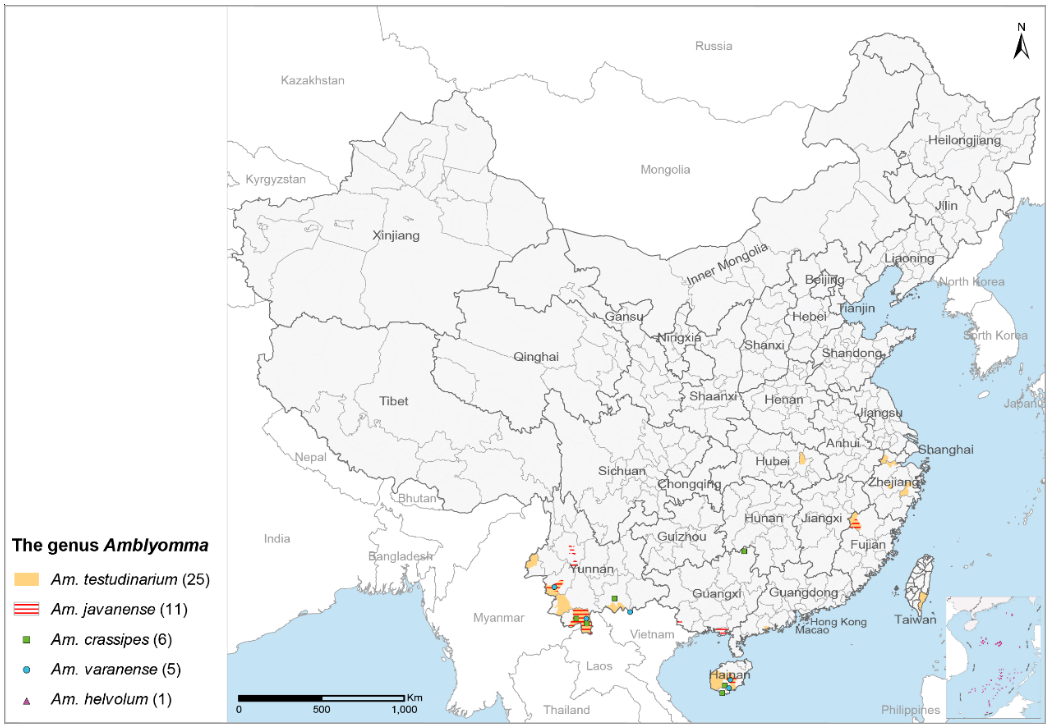

**Supplementary Fig. 4 The spatial distribution of the tick genus *Amblyomma* recorded at the county level from 1950 to 2018 in China.** Source data are provided as a Source Data file.

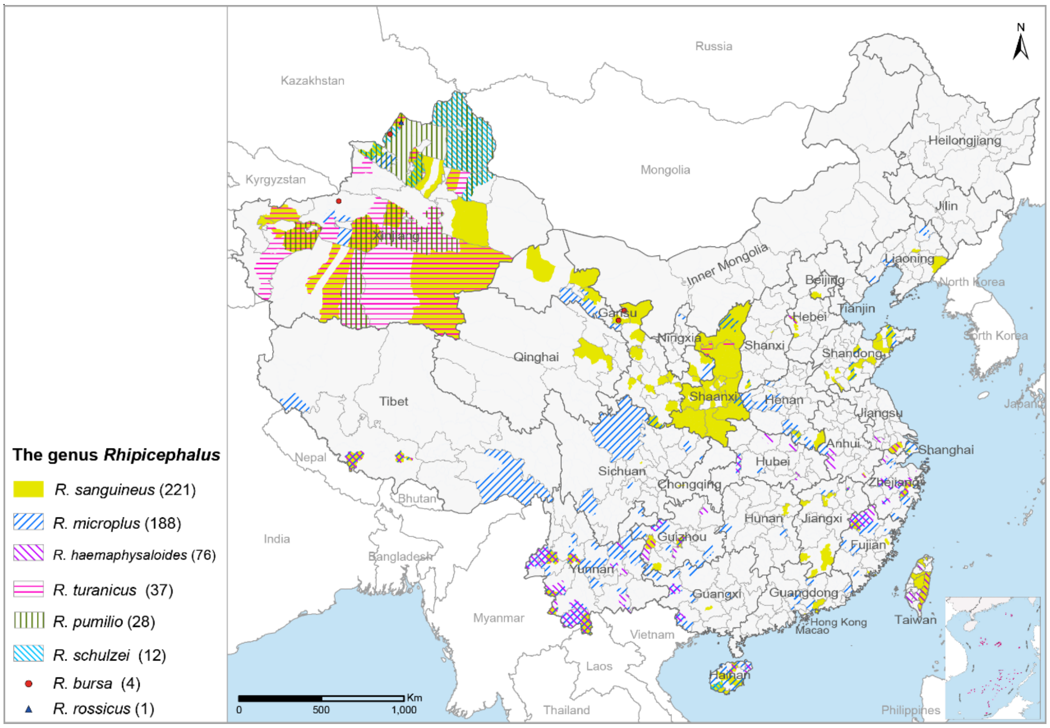


**Supplementary Fig. 5 The spatial distribution of the tick genus *Rhipicephalus* recorded at the county level from 1950 to 2018 in China.** Source data are provided as a Source Data file.

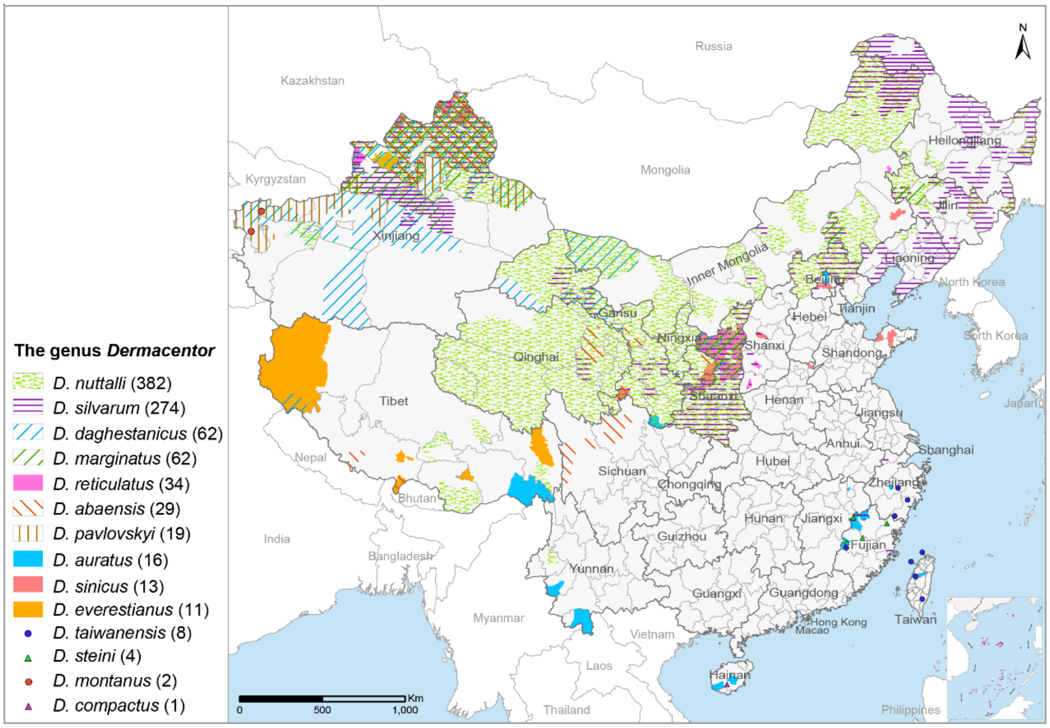


**Supplementary Fig. 6 The spatial distribution of the tick genus *Dermacentor* recorded at the county level from 1950 to 2018 in China.** Source data are provided as a Source Data file.

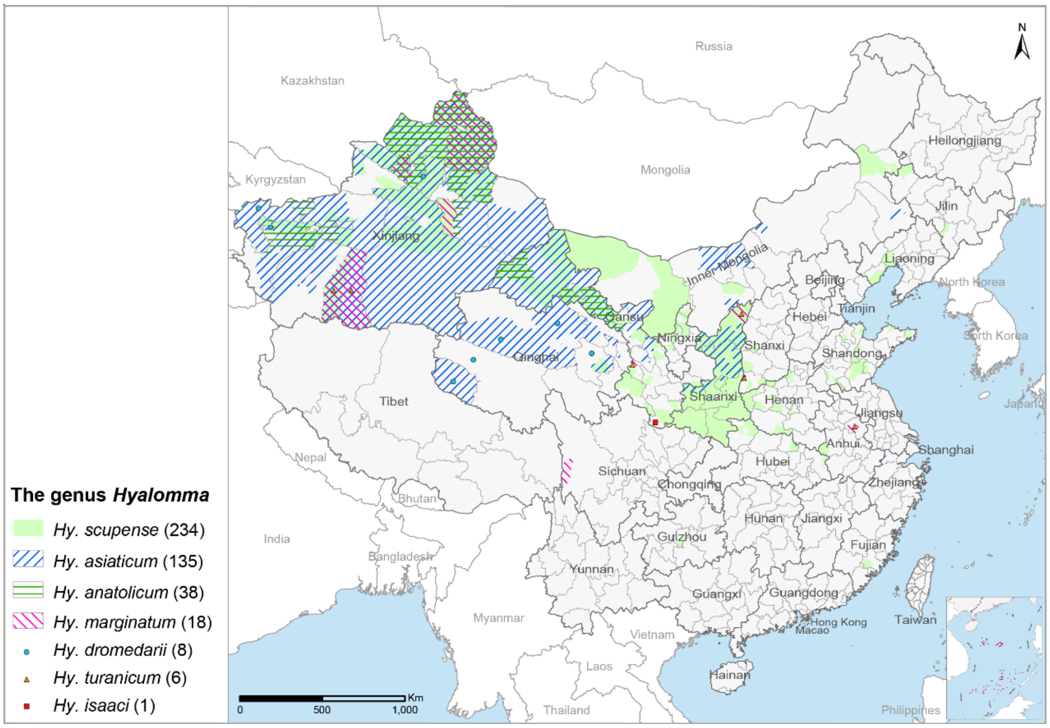


**Supplementary Fig. 7 The spatial distribution of the tick genus *Hyalomma* recorded at the county level from 1950 to 2018 in China.** Source data are provided as a Source Data file.

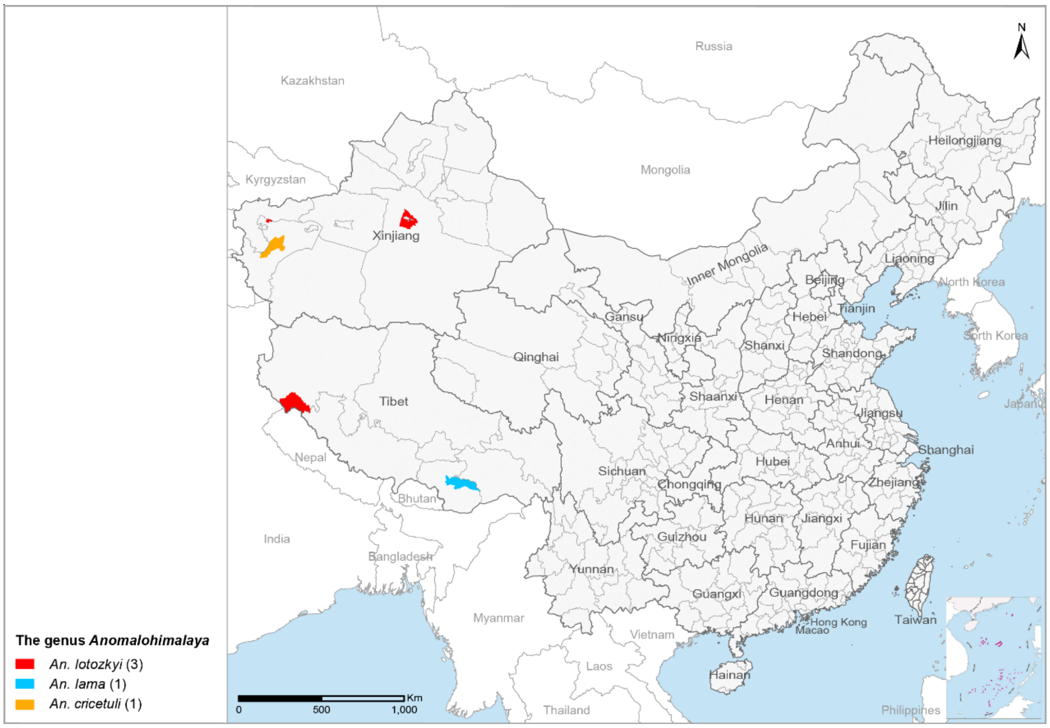


**Supplementary Fig. 8 The spatial distribution of the tick genus *Anomalohimalaya* recorded at the county level from 1950 to 2018 in China.** Source data are provided as a Source Data file.

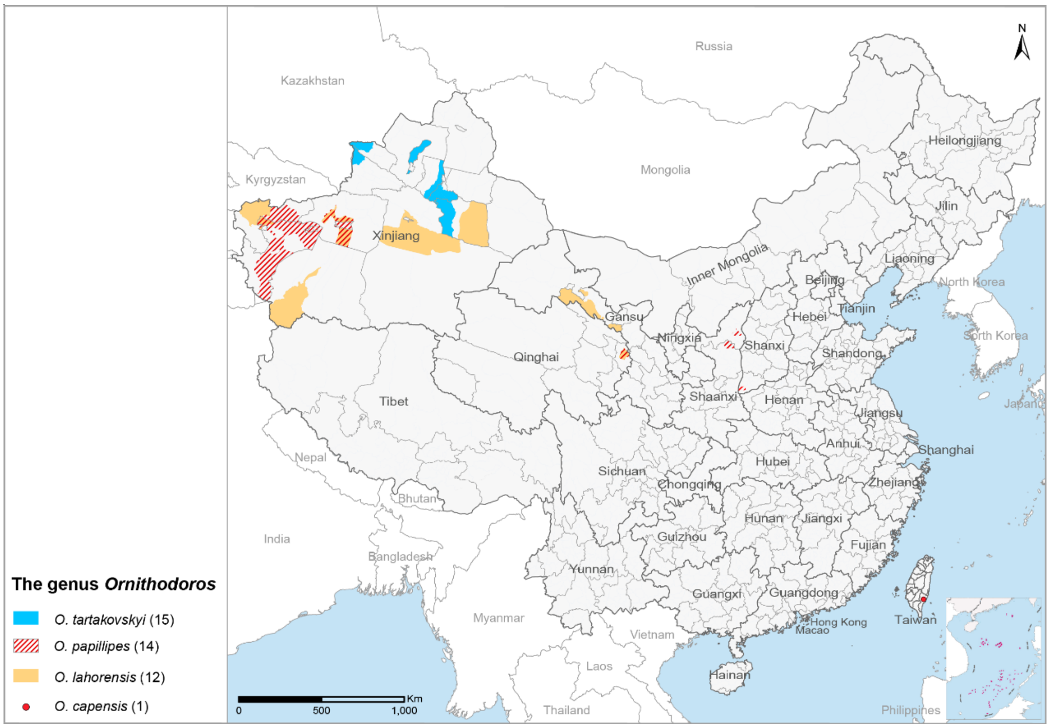


**Supplementary Fig. 9 The spatial distribution of the tick genus *Ornithodoros* recorded at the county level from 1950 to 2018 in China.** Source data are provided as a Source Data file.

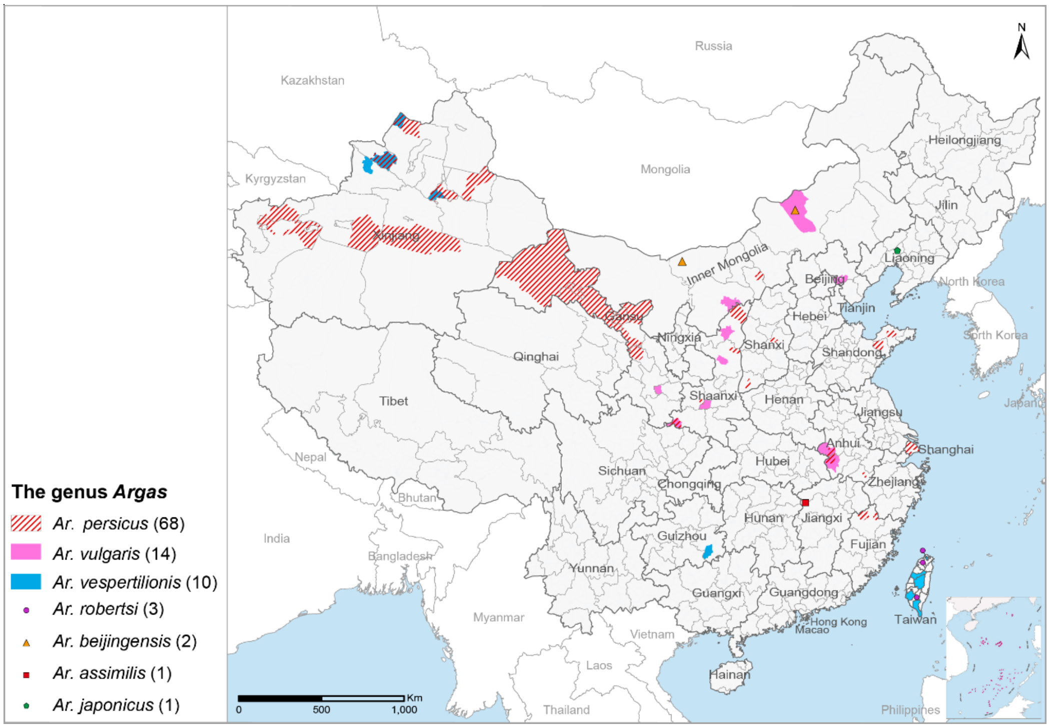


**Supplementary Fig. 10 The spatial distribution of the tick genus *Argas* recorded at the county level from 1950 to 2018 in China.** Source data are provided as a Source Data file.

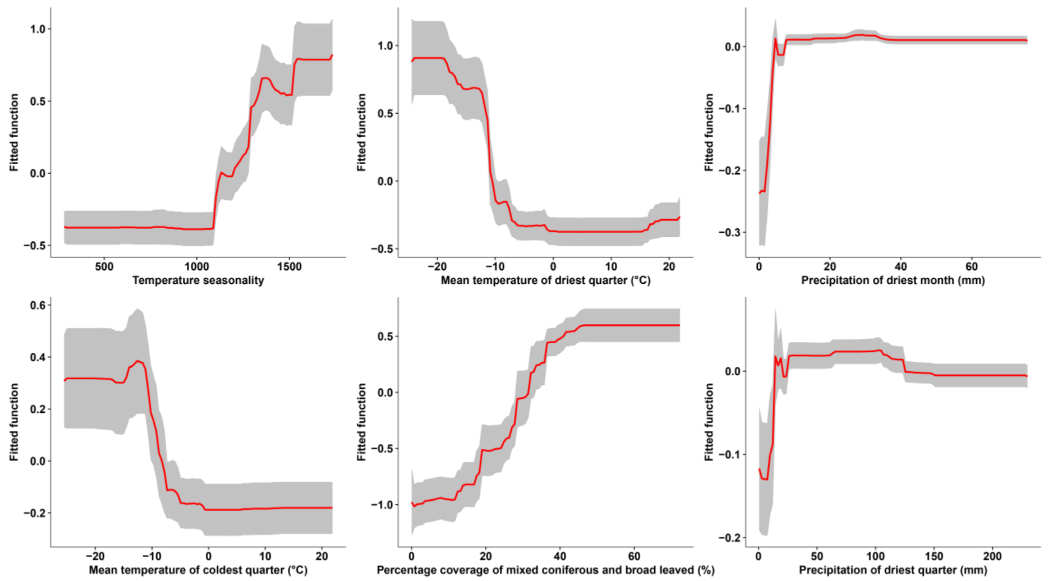


**Supplementary Fig. 11 The mean curves (red) and 95% percentiles (gray) for the effects of major predictors (RC≥5%) on the logit-transformed probability of occurrence of *I. persulcatus* based on the ensemble of BRT models.**

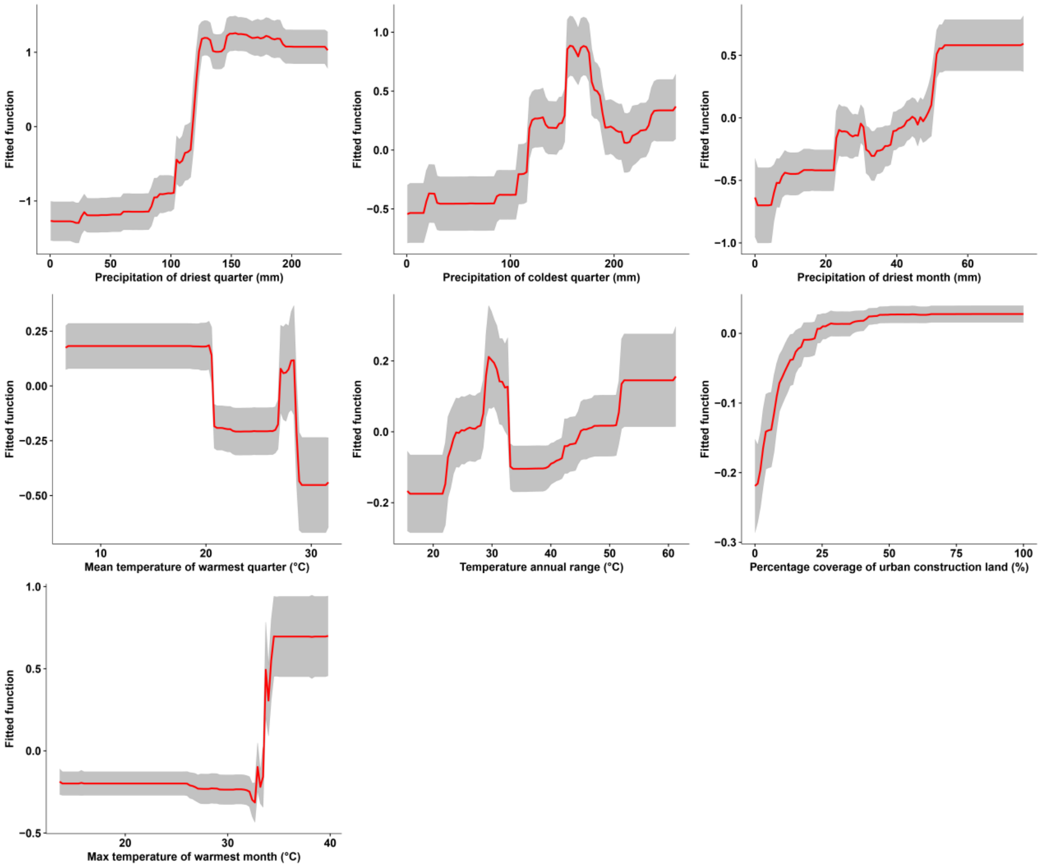


**Supplementary Fig. 12 The mean curves (red) and 95% percentiles (gray) for the effects of major predictors (RC≥5%) on the logit-transformed probability of occurrence of *I. sinensis* based on the ensemble of BRT models.**

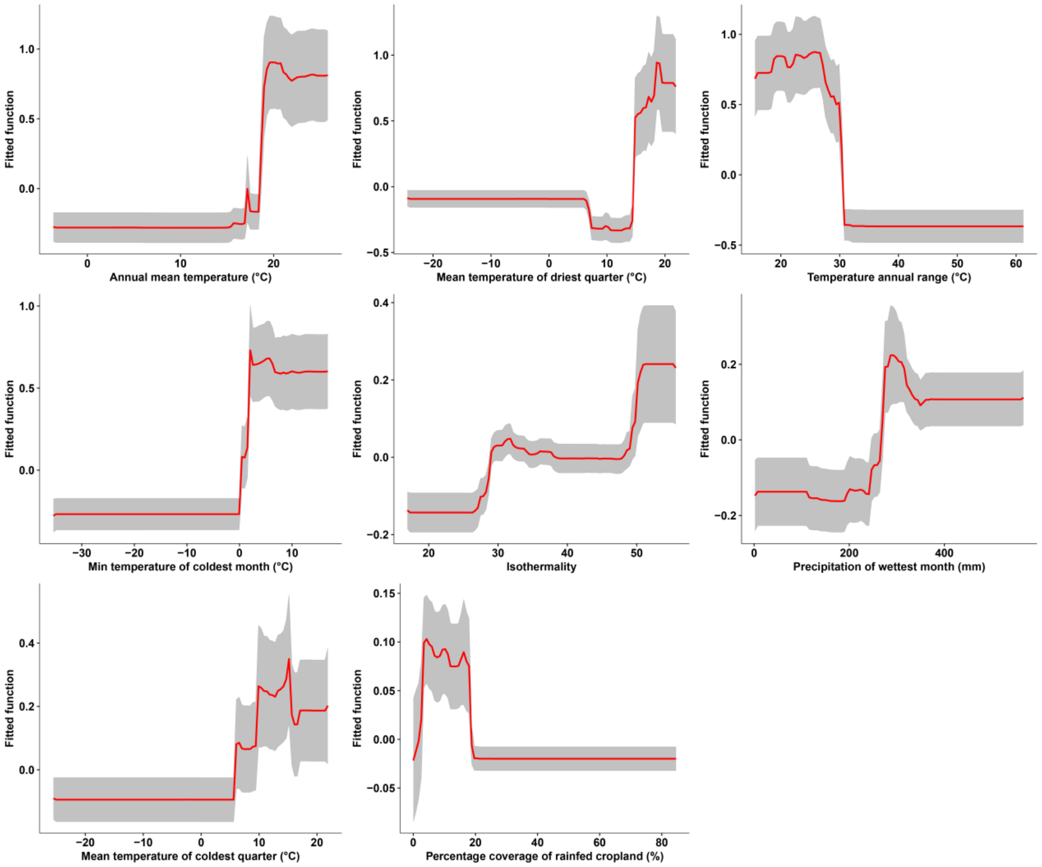


**Supplementary Fig. 13 The mean curves (red) and 95% percentiles (gray) for the effects of major predictors (RC≥5%) on the logit-transformed probability of occurrence of *I. granulatus* based on the ensemble of BRT models.**

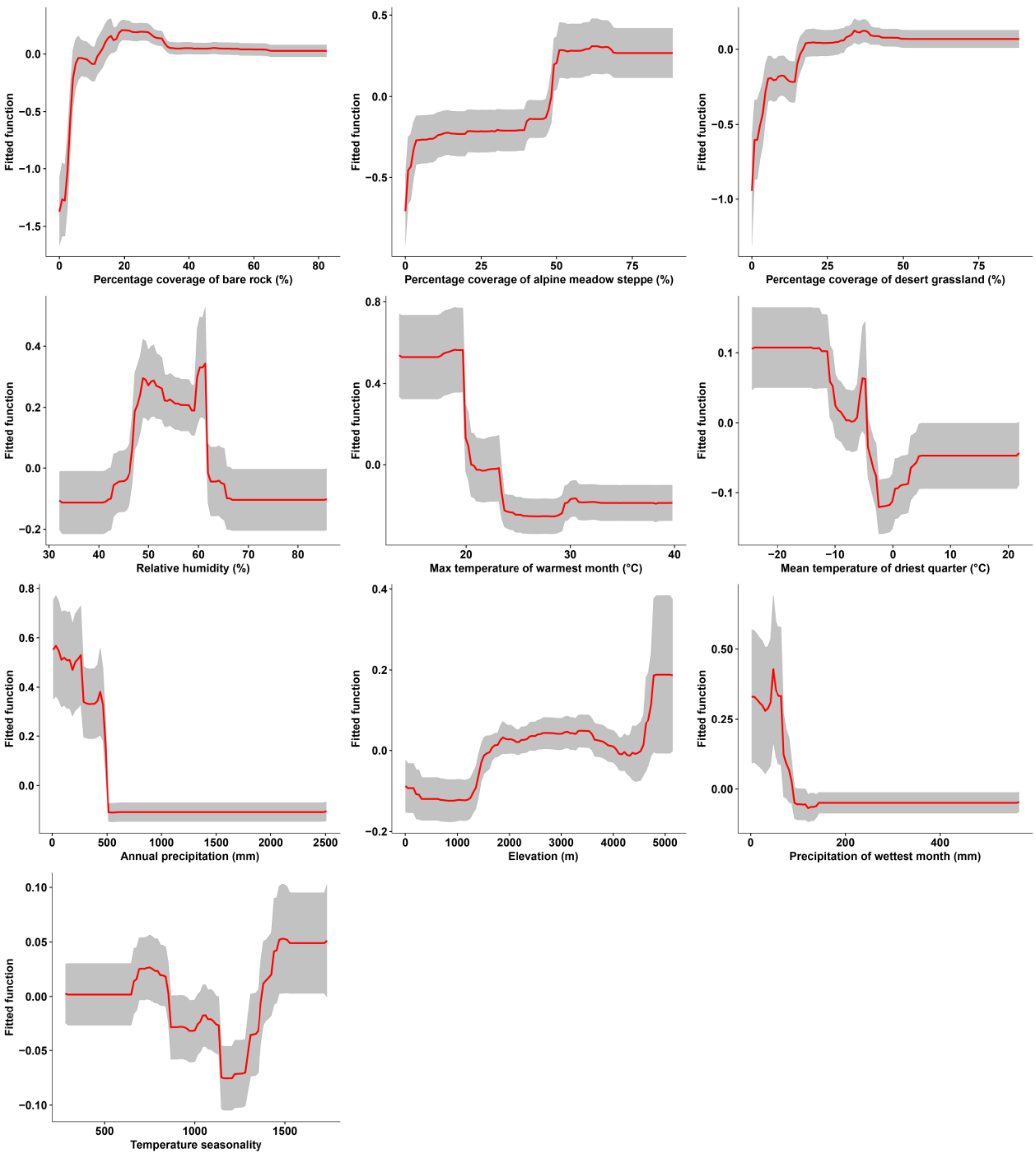


**Supplementary Fig. 14 The mean curves (red) and 95% percentiles (gray) for the effects of major predictors (RC≥5%) on the logit-transformed probability of occurrence of *I. crenulatus* based on the ensemble of BRT models.**

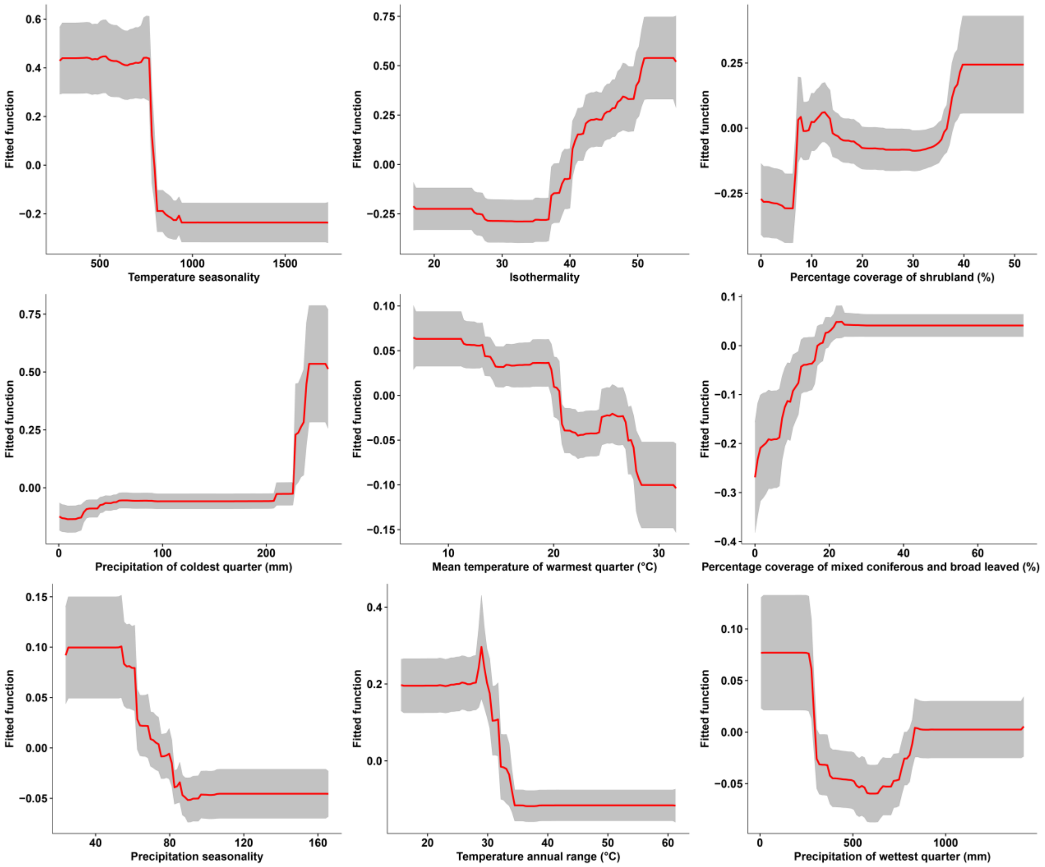


**Supplementary Fig. 15 The mean curves (red) and 95% percentiles (gray) for the effects of major predictors (RC≥5%) on the logit-transformed probability of occurrence of *I. ovatus* based on the ensemble of BRT models.**

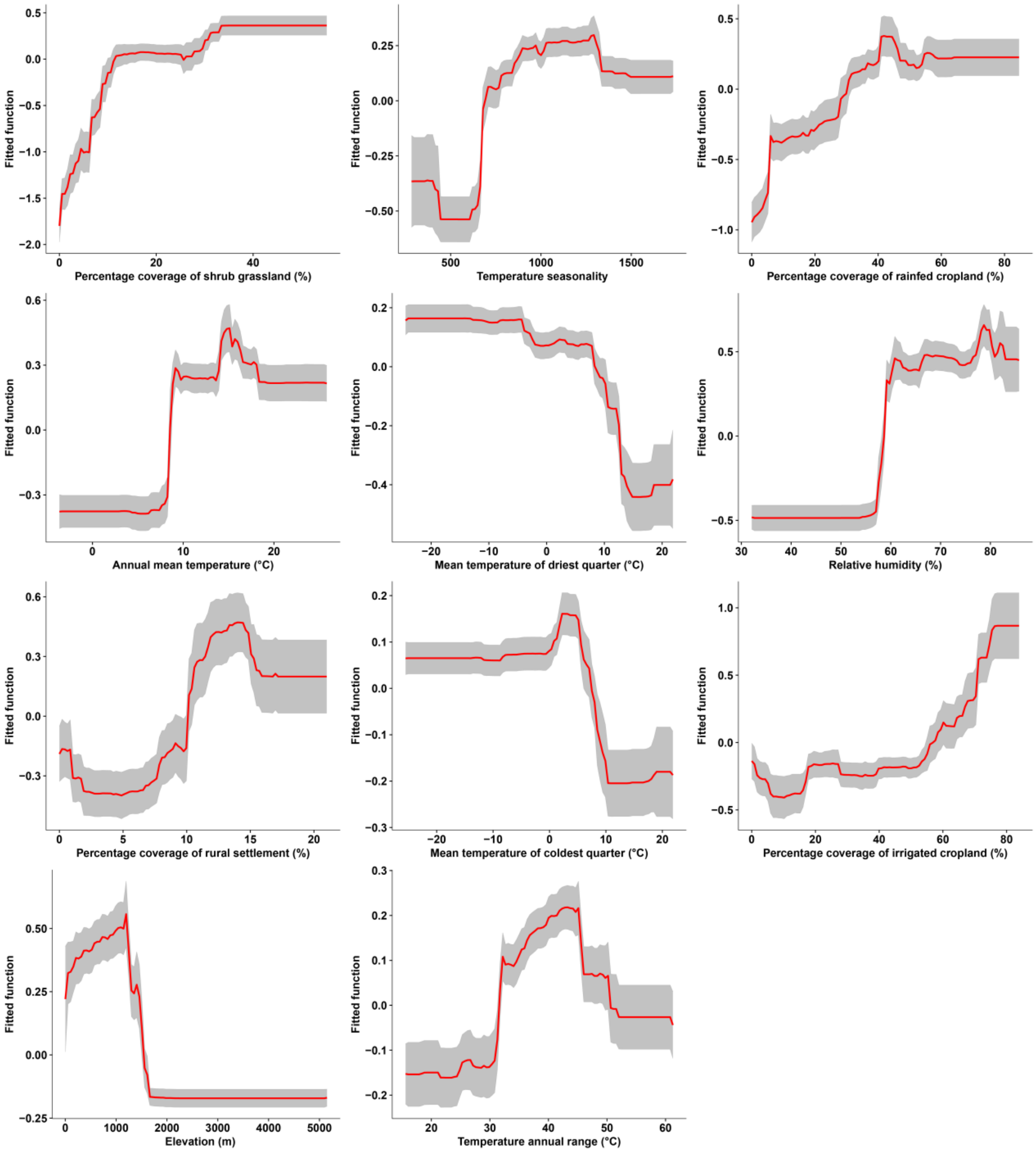


**Supplementary Fig. 16 The mean curves (red) and 95% percentiles (gray) for the effects of major predictors (RC≥5%) on the logit-transformed probability of occurrence of *Ha. longicornis* based on the ensemble of BRT models.**

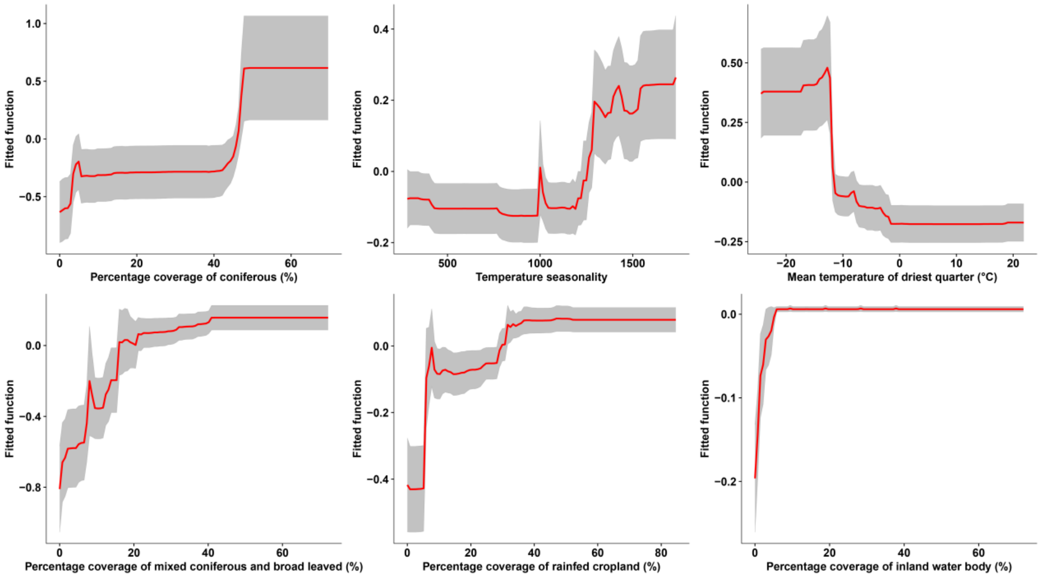


**Supplementary Fig. 17 The mean curves (red) and 95% percentiles (gray) for the effects of major predictors (RC≥5%) on the logit-transformed probability of occurrence of *Ha. concinna* based on the ensemble of BRT models.**

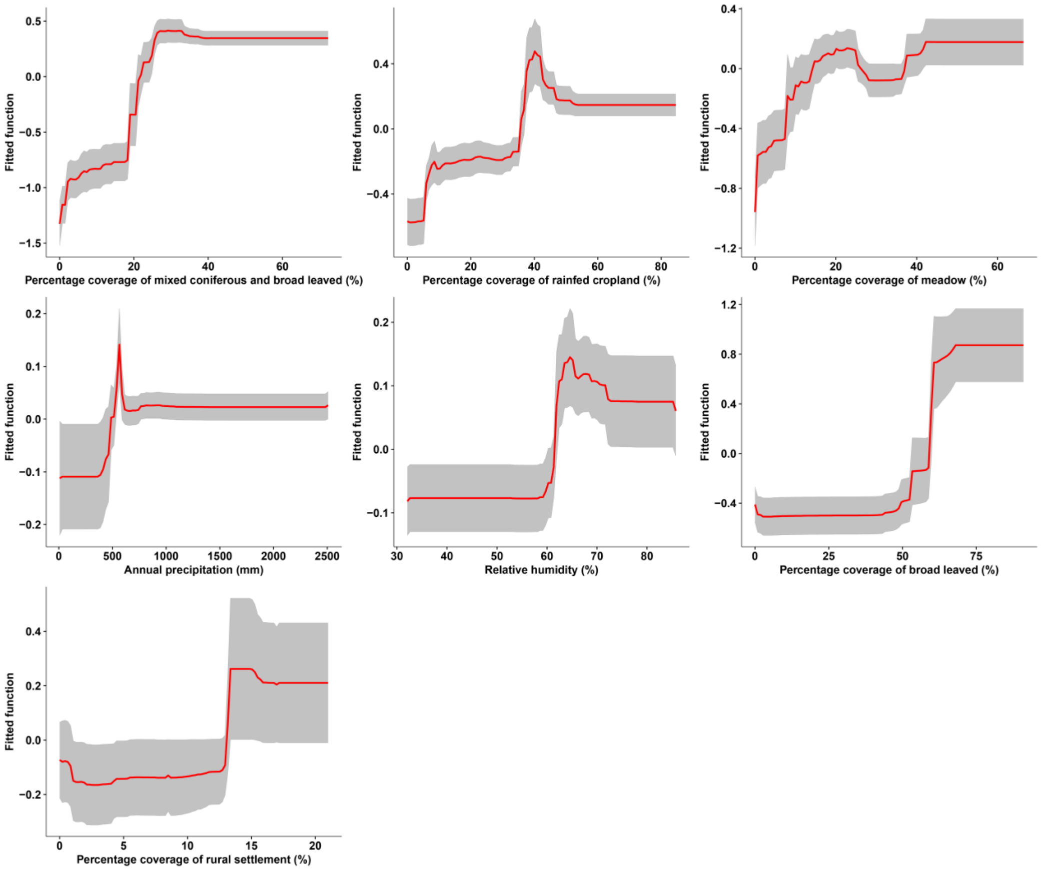


**Supplementary Fig. 18 The mean curves (red) and 95% percentiles (gray) for the effects of major predictors (RC≥5%) on the logit-transformed probability of occurrence of *Ha. japonica* based on the ensemble of BRT models.**

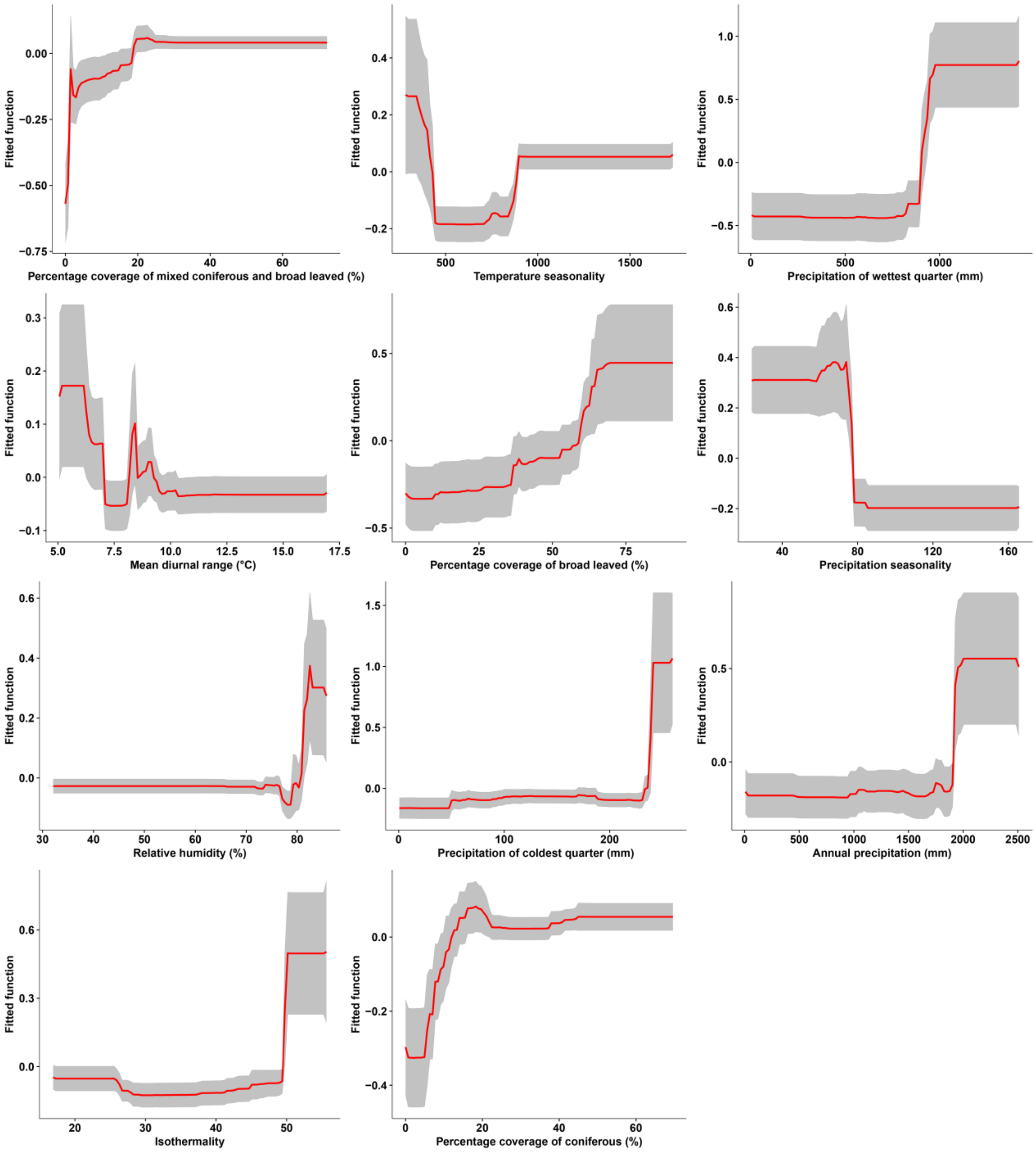


**Supplementary Fig. 19 The mean curves (red) and 95% percentiles (gray) for the effects of major predictors (RC≥5%) on the logit-transformed probability of occurrence of *Ha. hystricis* based on the ensemble of BRT models.**

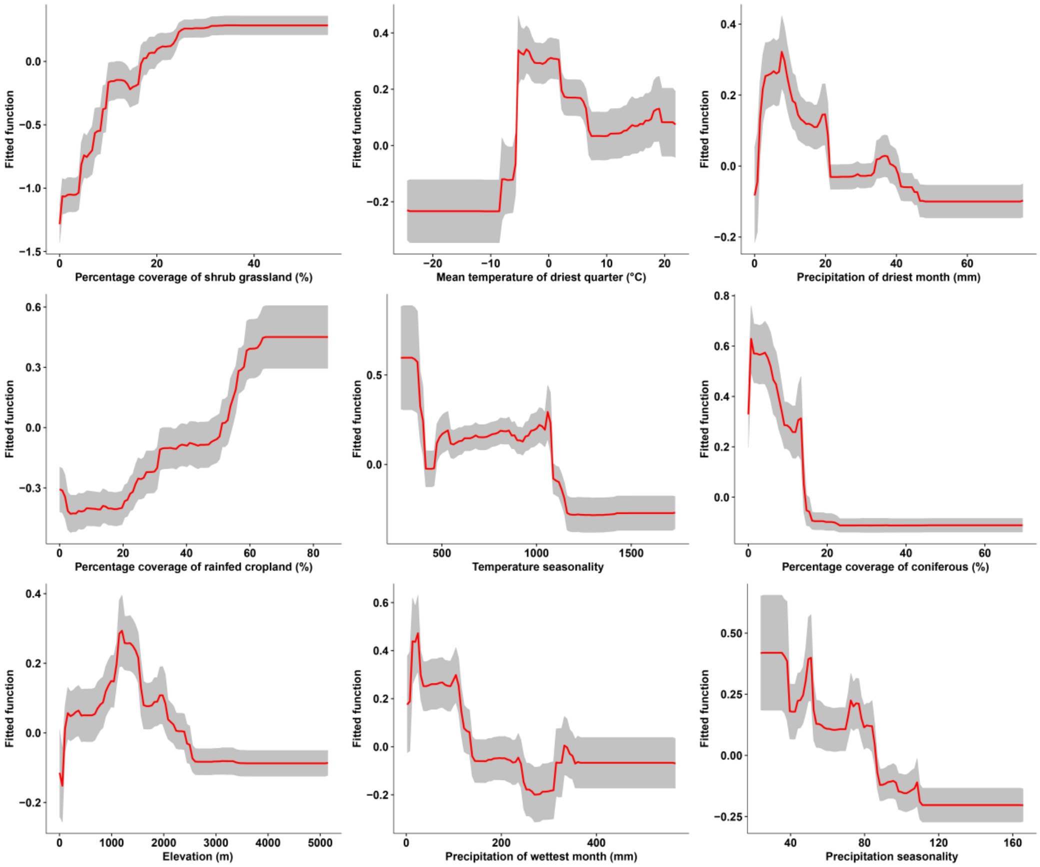


**Supplementary Fig. 20 The mean curves (red) and 95% percentiles (gray) for the effects of major predictors (RC≥5%) on the logit-transformed probability of occurrence of *R. sanguineus* based on the ensemble of BRT models.**

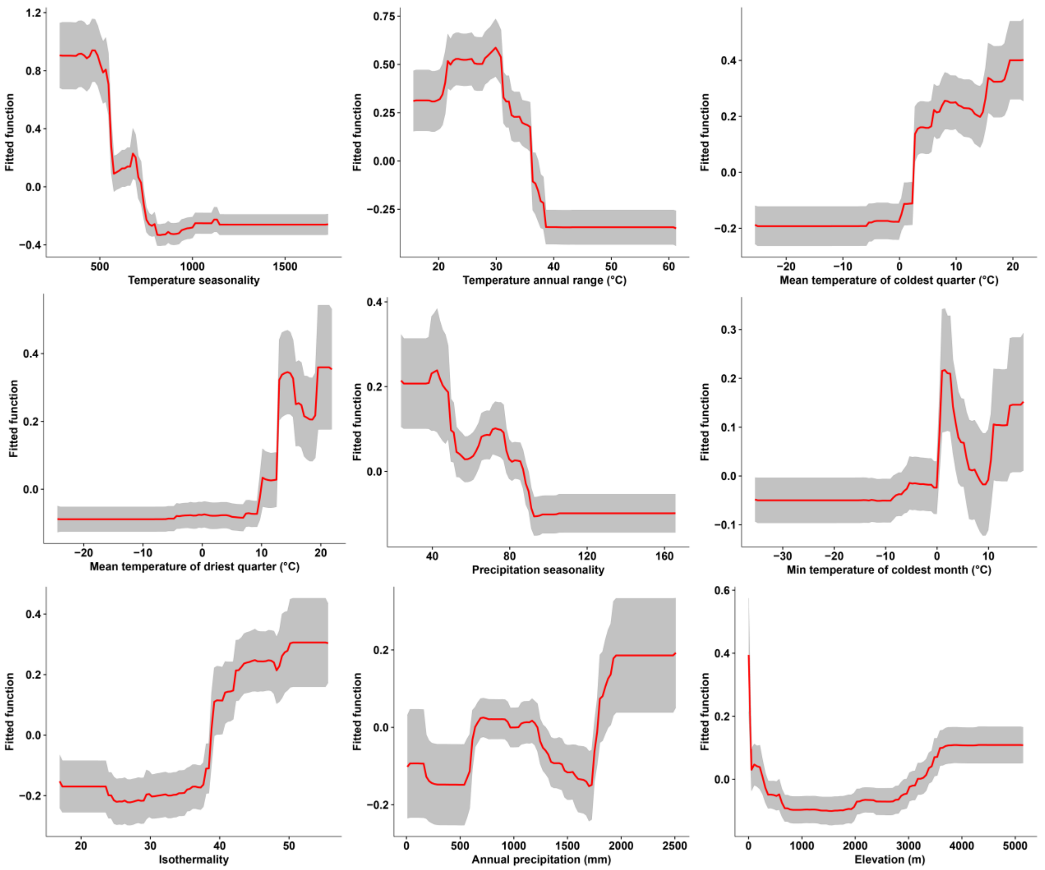


**Supplementary Fig. 21 The mean curves (red) and 95% percentiles (gray) for the effects of major predictors (RC≥5%) on the logit-transformed probability of occurrence of *R. microplus* based on the ensemble of BRT models.**

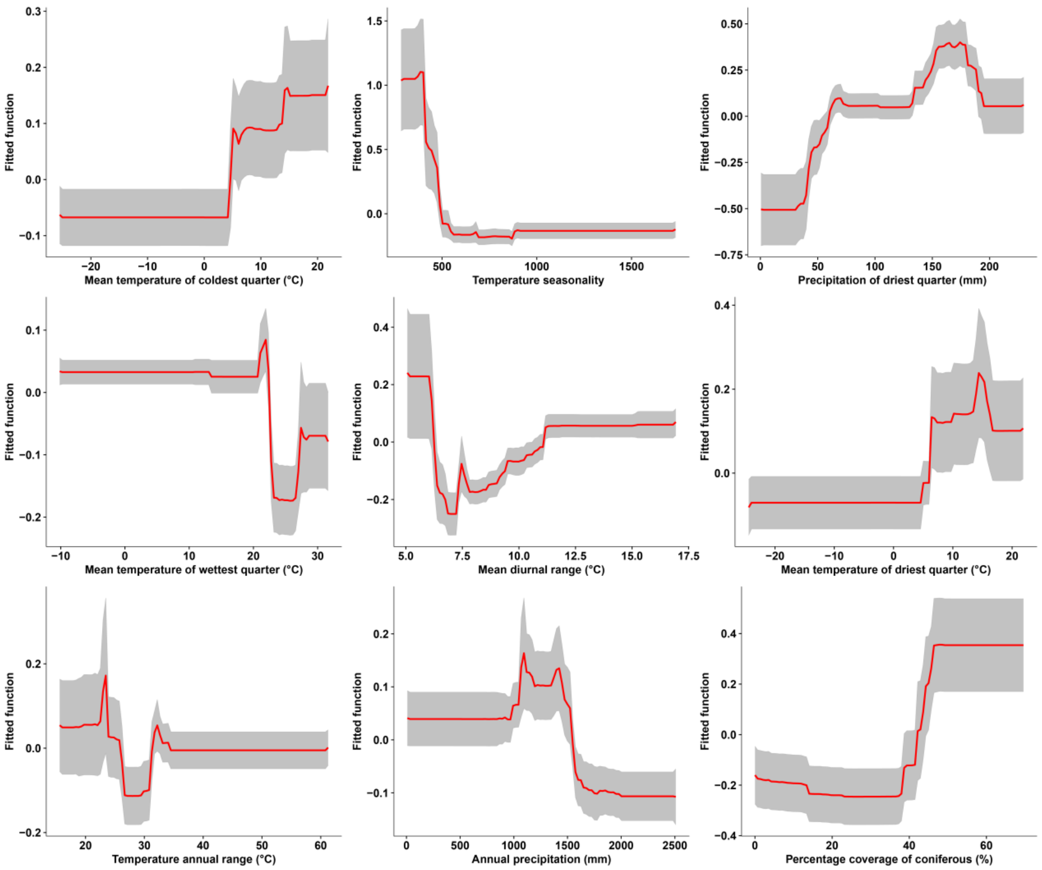


**Supplementary Fig. 22 The mean curves (red) and 95% percentiles (gray) for the effects of major predictors (RC≥5%) on the logit-transformed probability of occurrence of *R. haemaphysaloides* based on the ensemble of BRT models.**

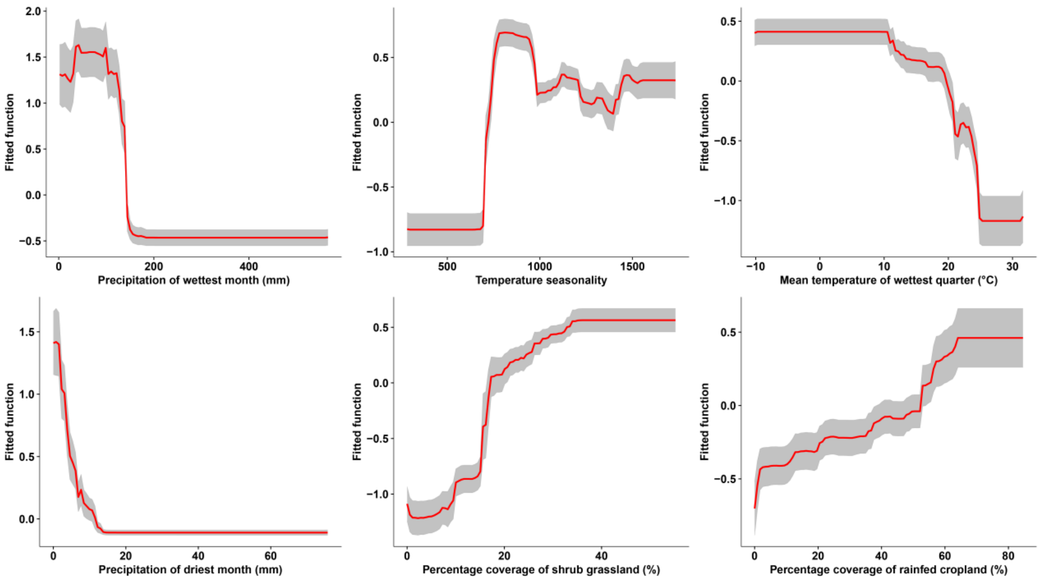


**Supplementary Fig. 23 The mean curves (red) and 95% percentiles (gray) for the effects of major predictors (RC≥5%) on the logit-transformed probability of occurrence of *D. nuttalli* based on the ensemble of BRT models.**

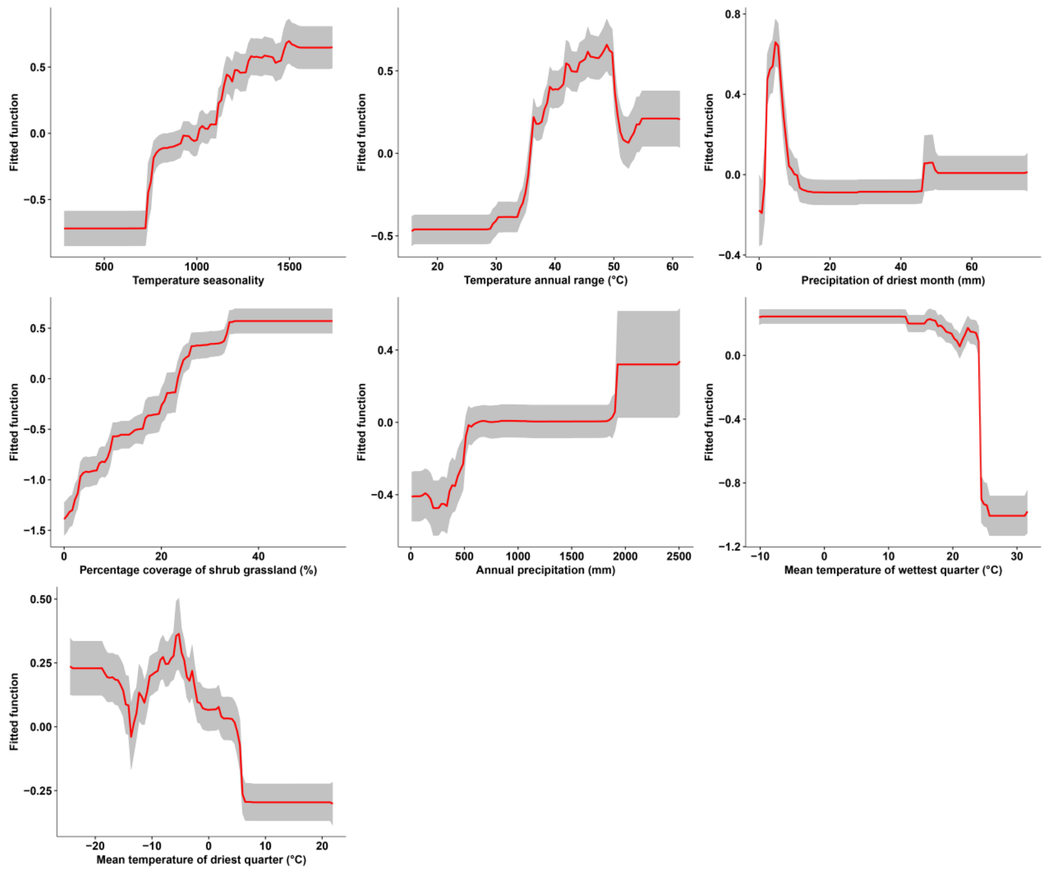


**Supplementary Fig. 24 The mean curves (red) and 95% percentiles (gray) for the effects of major predictors (RC≥5%) on the logit-transformed probability of occurrence of *D. silvarum* based on the ensemble of BRT models.**

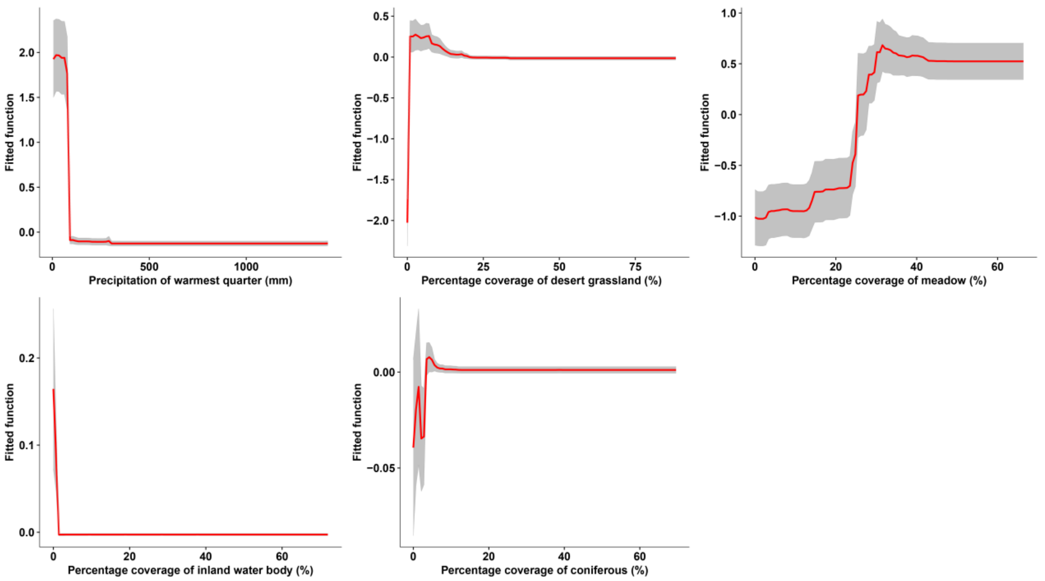


**Supplementary Fig. 25 The mean curves (red) and 95% percentiles (gray) for the effects of major predictors (RC≥5%) on the logit-transformed probability of occurrence of *D. daghestanicus* based on the ensemble of BRT models.**

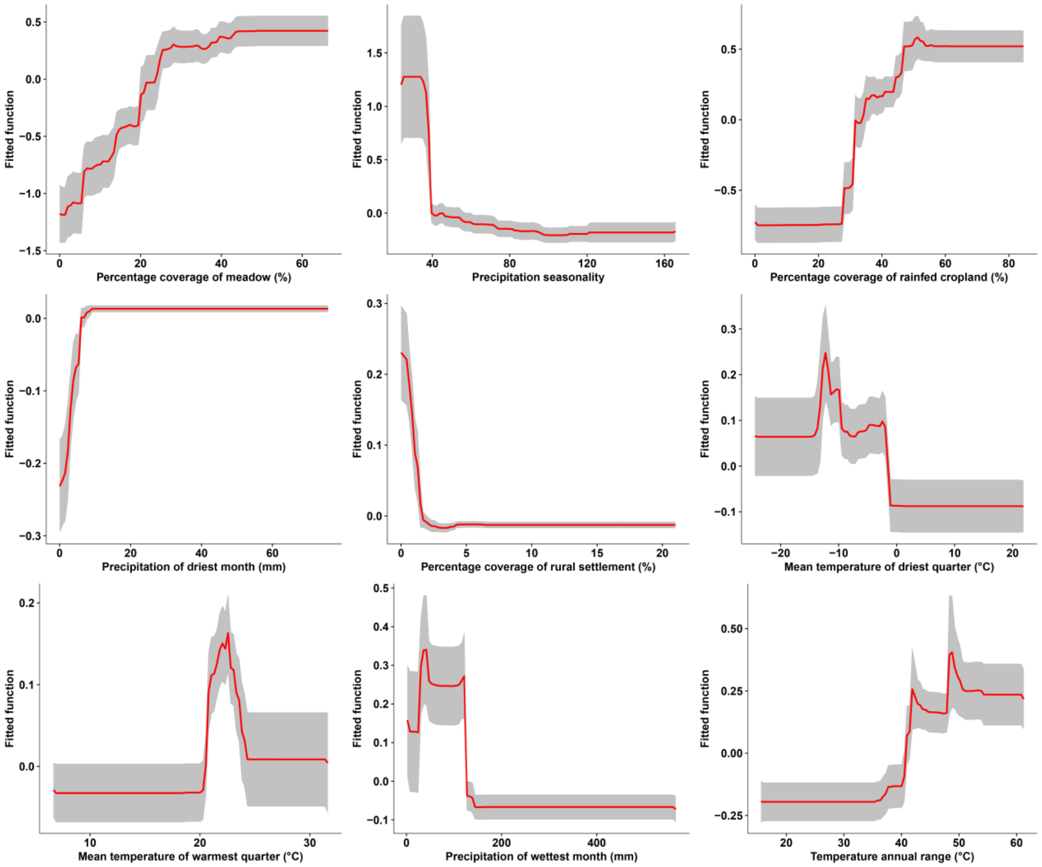


**Supplementary Fig. 26 The mean curves (red) and 95% percentiles (gray) for the effects of major predictors (RC≥5%) on the logit-transformed probability of occurrence of *D. marginatus* based on the ensemble of BRT models.**

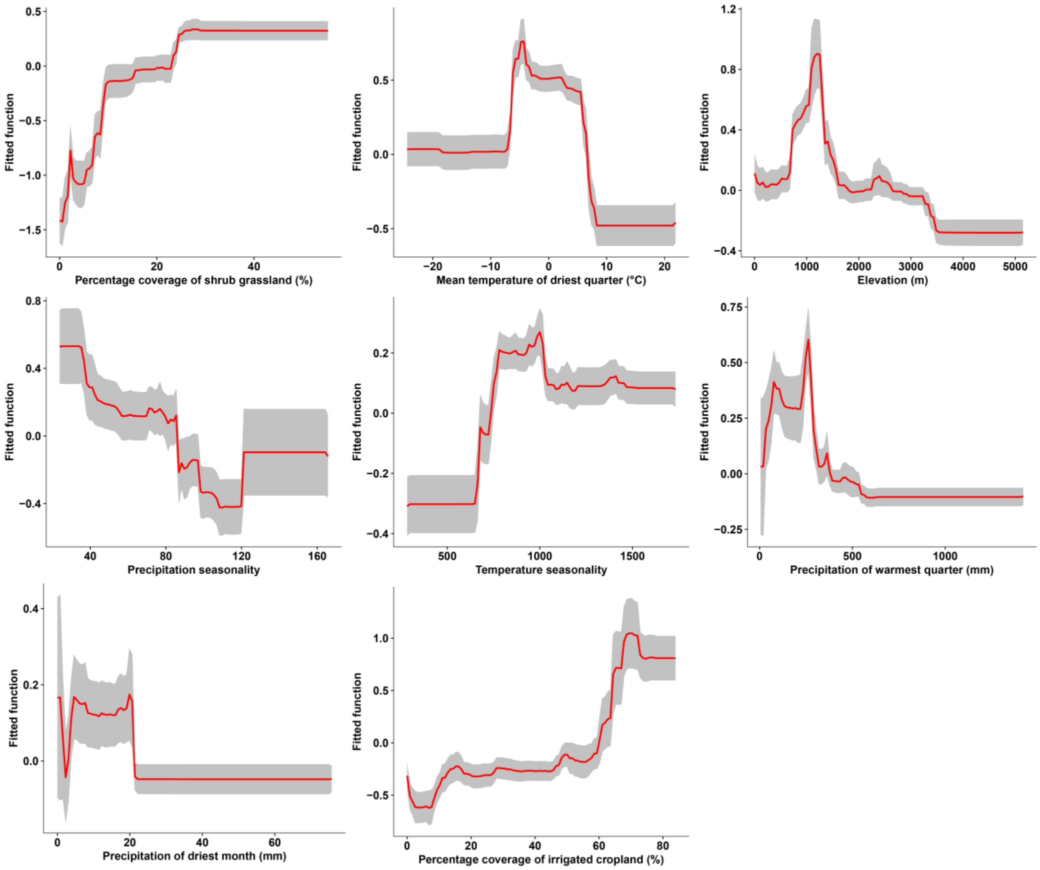


**Supplementary Fig. 27 The mean curves (red) and 95% percentiles (gray) for the effects of major predictors (RC≥5%) on the logit-transformed probability of occurrence of *Hy. scupense* based on the ensemble of BRT models.**

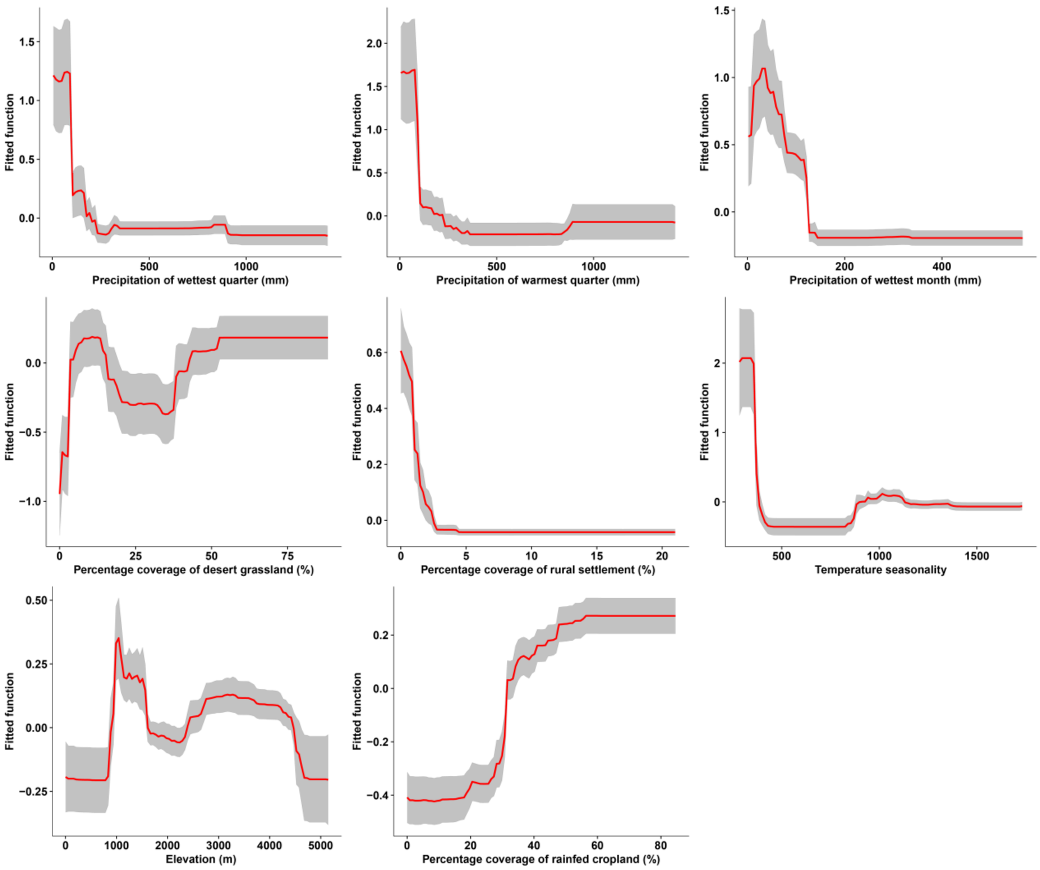


**Supplementary Fig. 28 The mean curves (red) and 95% percentiles (gray) for the effects of major predictors (RC≥5%) on the logit-transformed probability of occurrence of *Hy. asiaticum* based on the ensemble of BRT models.**

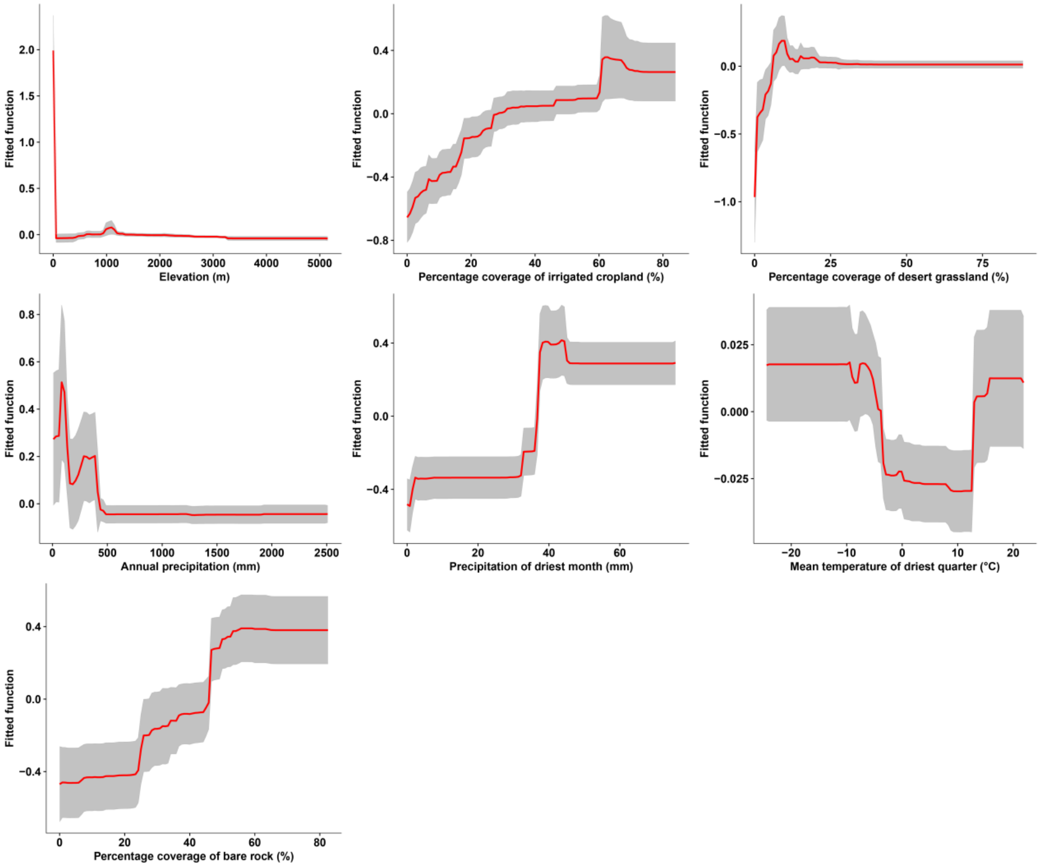


**Supplementary Fig. 29 The mean curves (red) and 95% percentiles (gray) for the effects of major predictors (RC≥5%) on the logit-transformed probability of occurrence of *Ar. persicus* based on the ensemble of BRT models.**

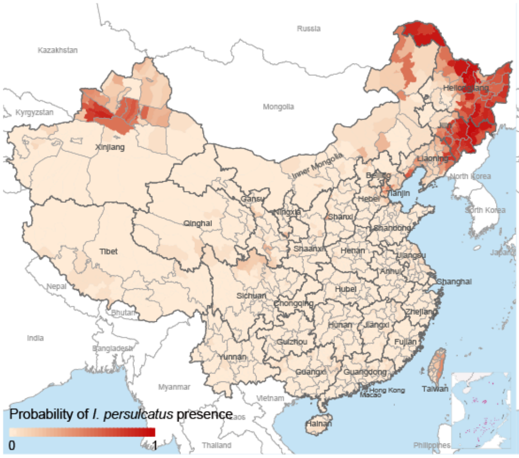


(a) *I. persulcatus*

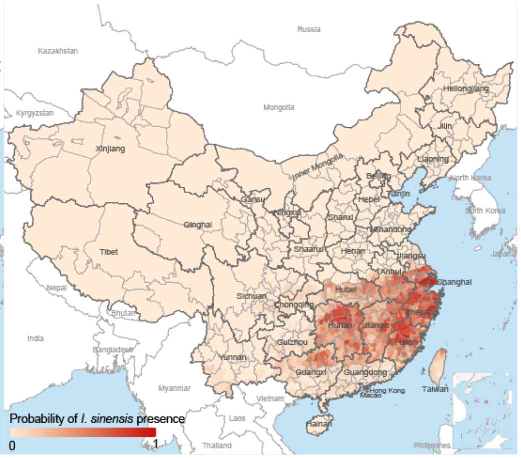


(b) *I. sinensis*

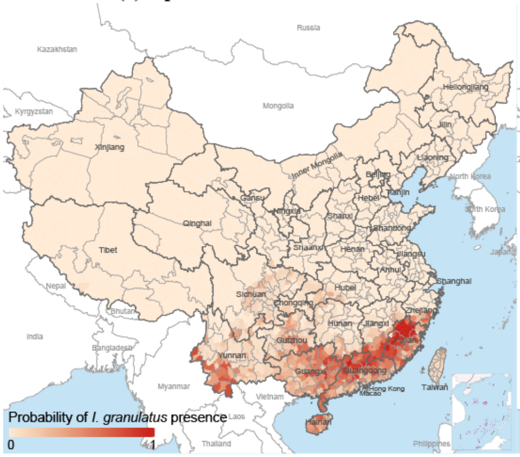


(c) *I. granulatus*

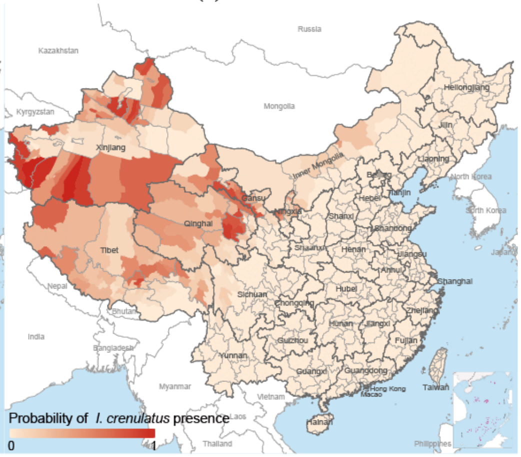


(d) *I. crenulatus*

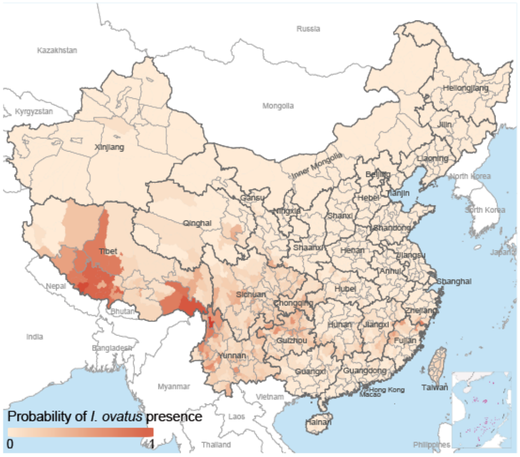


(e) *I. ovatus*

**Supplementary Fig. 30 The predicted county-level distributions of the five most prevalent tick species in the *Ixodes* genus, averaged over the ensemble of BRT models (a) *I. persulcatus*, (b) *I. sinensis*, (c) *I. granulatus*, (d) *I. crenulatus*, and (e) *I. ovatus*.** Source data are provided as a Source Data file.

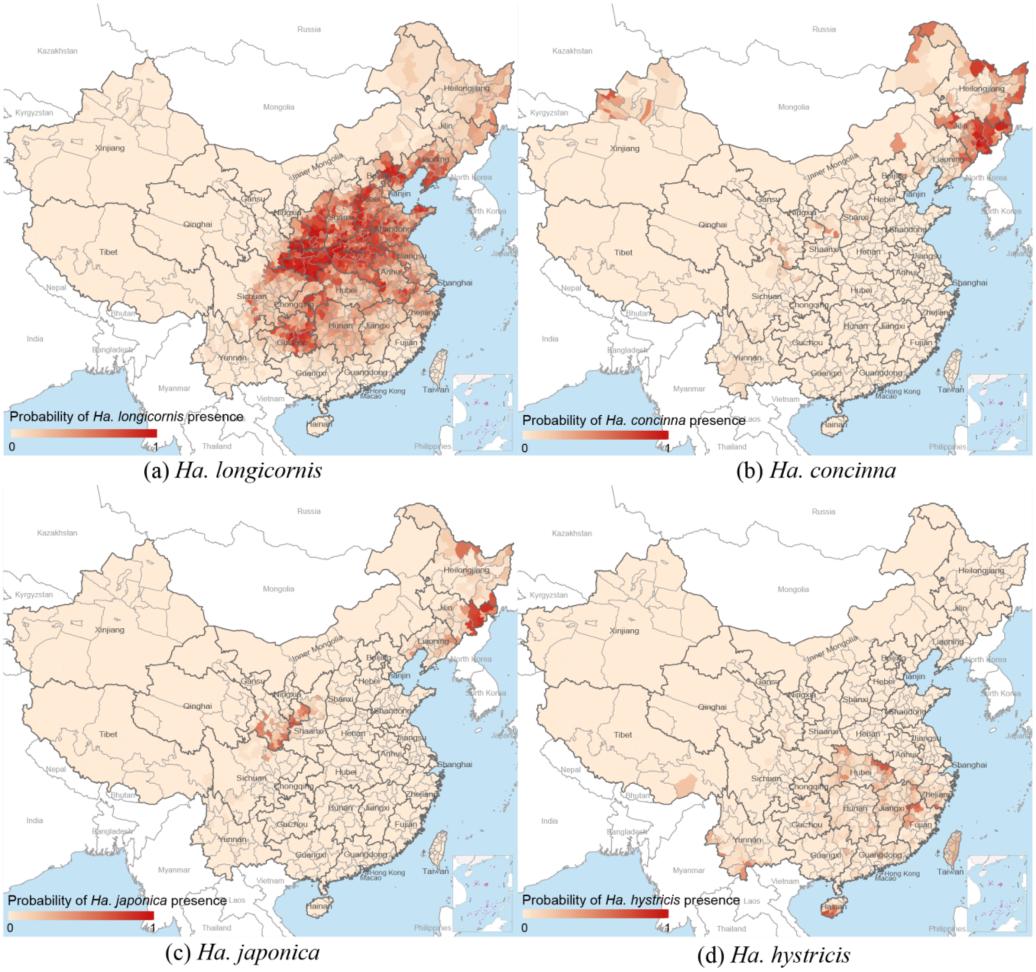


**Supplementary Fig. 31 The predicted county-level distributions of the four most prevalent tick species in the *Haemaphysalis* genus, averaged over the ensemble of BRT models (a) *Ha. longicornis*, (b) *Ha. concinna*, (c) *Ha. japonica*, and (d) *Ha. hystricis*.** Source data are provided as a Source Data file.

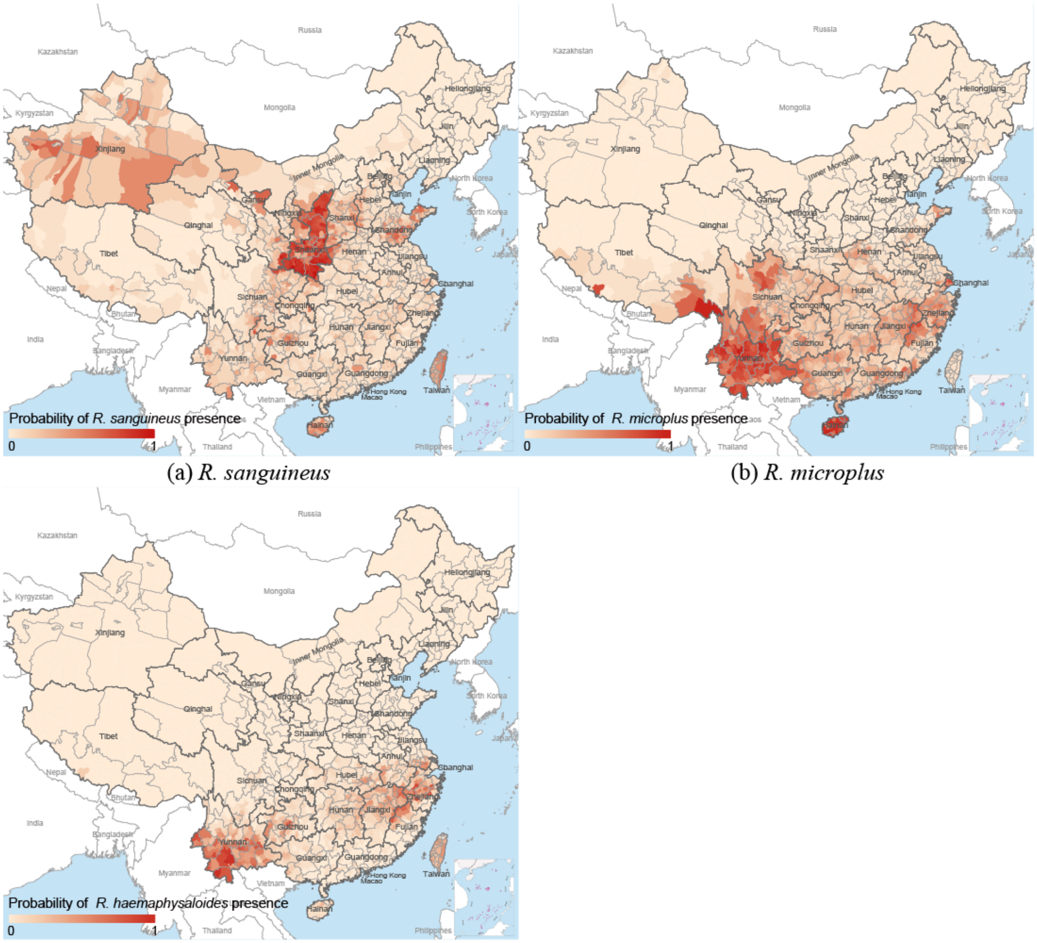


(a) *R. sanguineus* (b) *R. microplus*

(c) *R. haemaphysaloides*

**Supplementary Fig. 32 The predicted county-level distributions of the three most prevalent tick species in the *Rhipicephalus* genus, averaged over the ensemble of BRT models (a) *R. sanguineus,* (b) *R. microplus*, and (c) *R. haemaphysaloides*.** Source data are provided as a Source Data file.

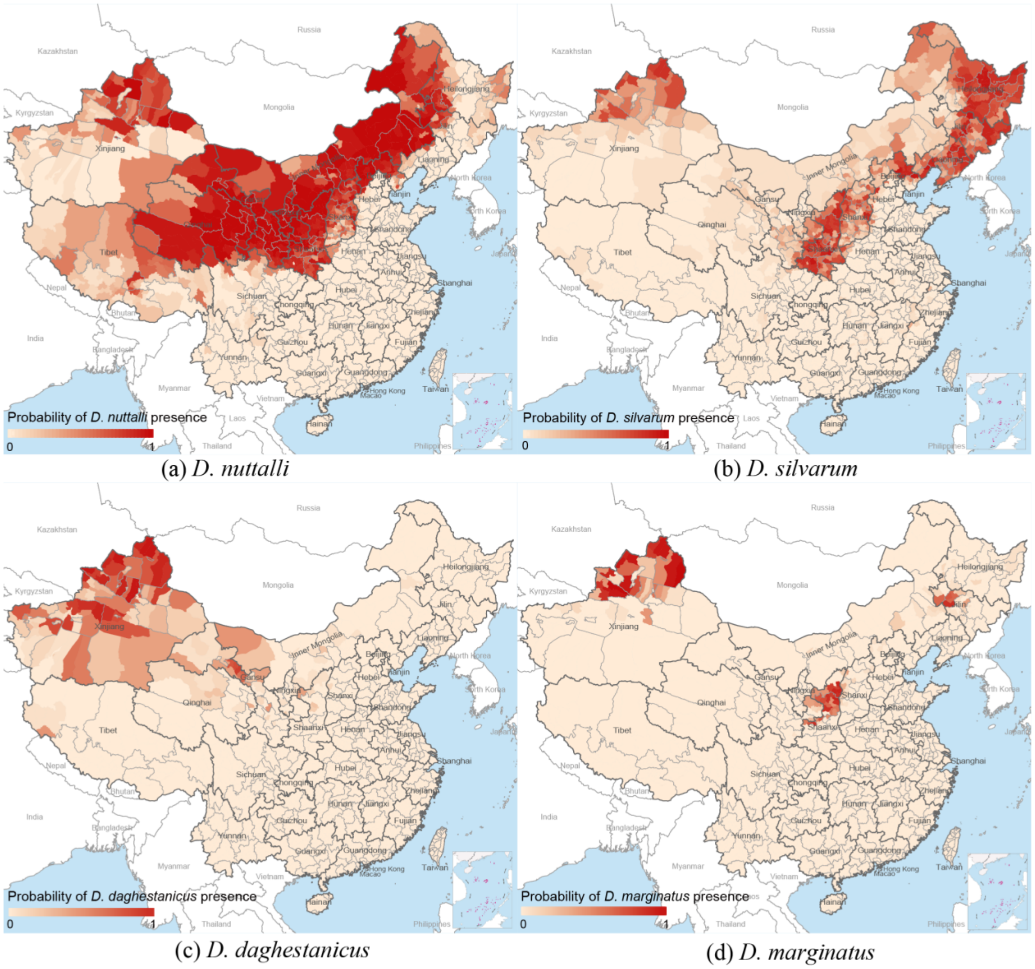


**Supplementary Fig. 33 The predicted county-level distributions of the four most prevalent tick species in the *Demacentor* genus, averaged over the ensemble of BRT models: (a) *D. nuttalli,* (b) *D. silvarum*, (c) *D. daghestanicus*, and (d) *D. marginatus*.** Source data are provided as a Source Data file.

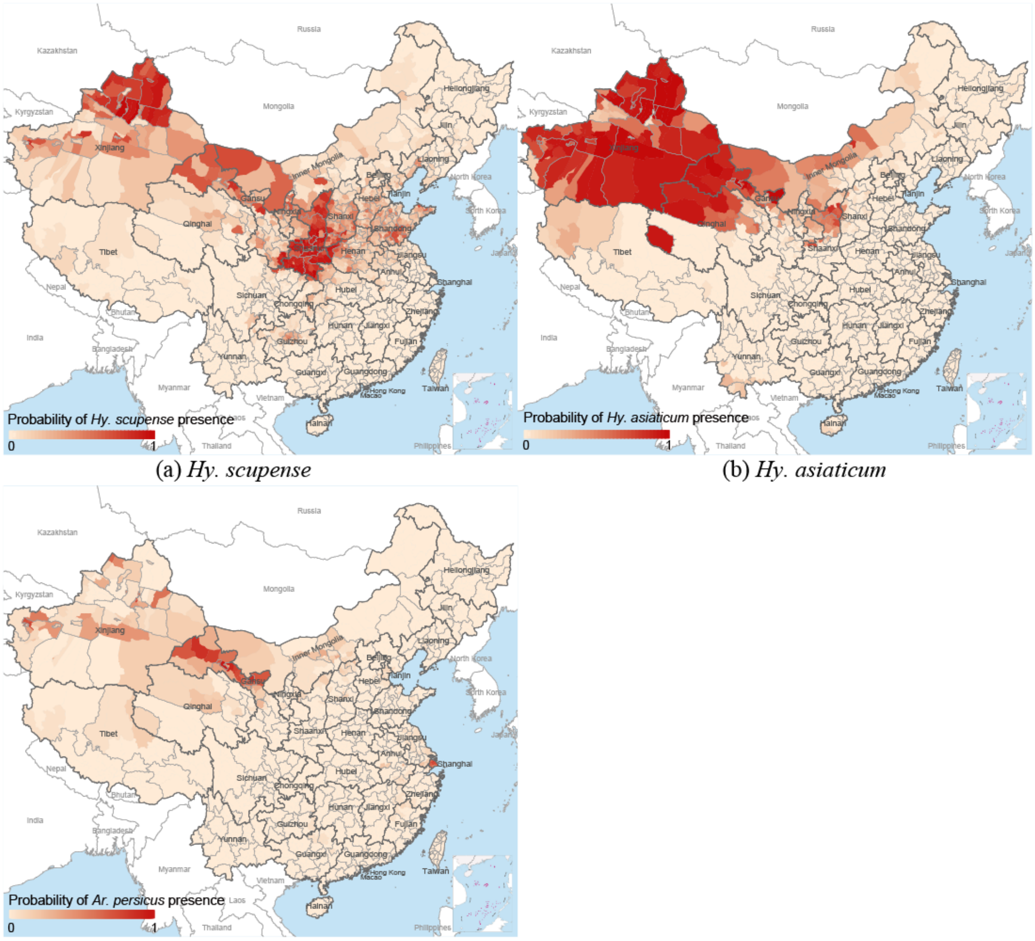


(a) *Hy. scupense* (b) *Hy. asiaticum*

(c) *Ar. persicus*

**Supplementary Fig. 34 The predicted county-level distributions of the two most prevalent tick species in the *Hyalomma* genus and one in the *Argas* genus, averaged over the ensemble of BRT models (a) *Hy. scupense,* (b) *Hy. asiaticum*, and (c) *Ar. persicus*.** Source data are provided as a Source Data file.

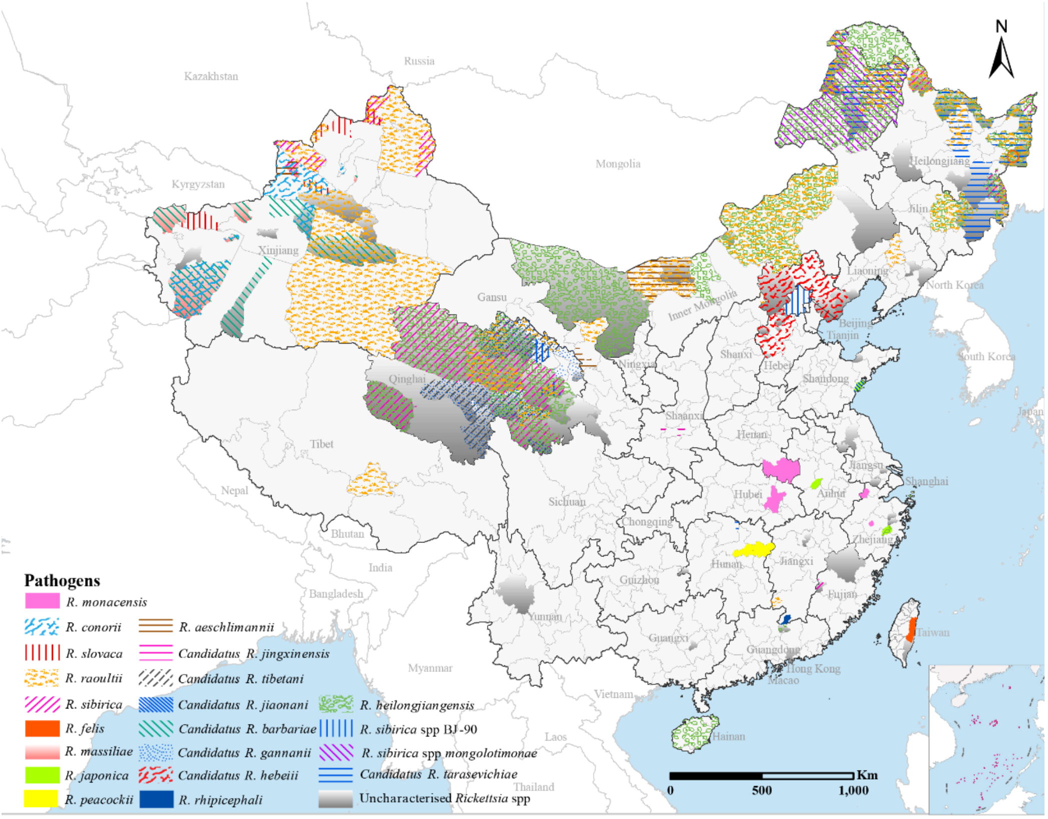


**Supplementary Fig. 35 The locations of species or subspecies of spotted fever group rickettsiae detected in ticks during 1950–2018 in China. Data were plotted at the county or prefecture level, depending on available resolution in the literature.** Source data are provided as a Source Data file.

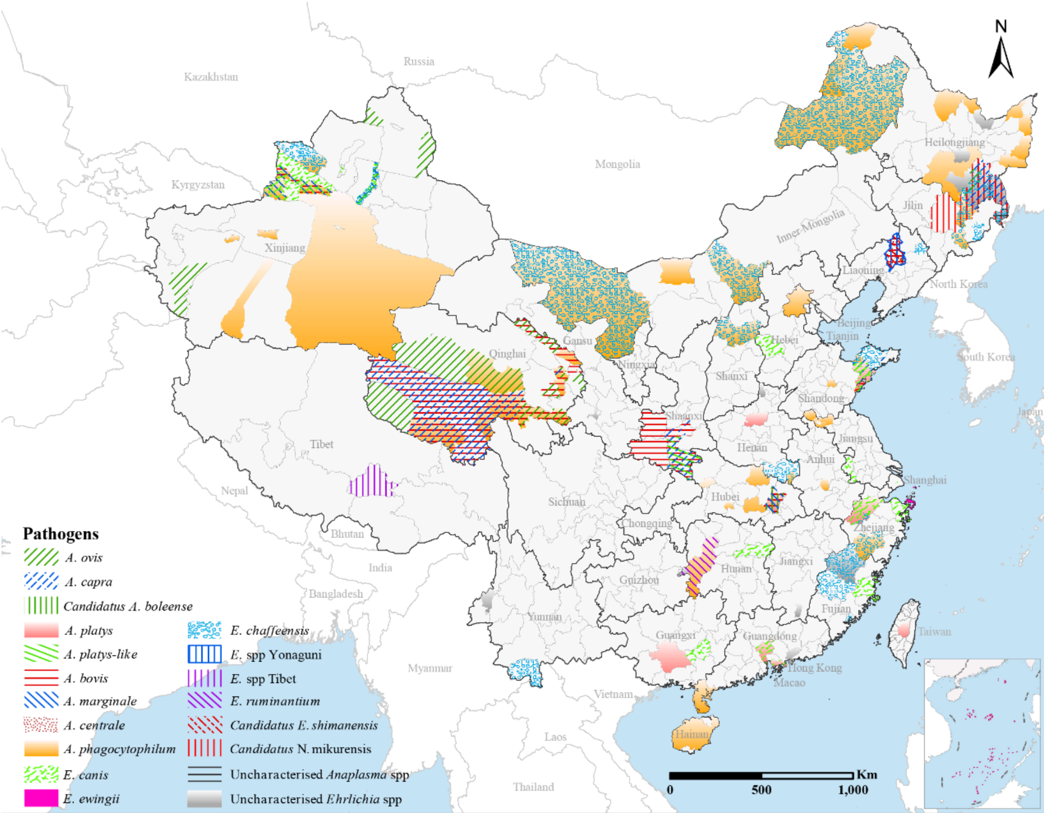


**Supplementary Fig. 36 The locations of species or subspecies of *Anaplasmataceae* detected in ticks during 1950–2018 in China.** Data were plotted at the county or prefecture level, depending on available resolution in the literature. Source data are provided as a Source Data file.

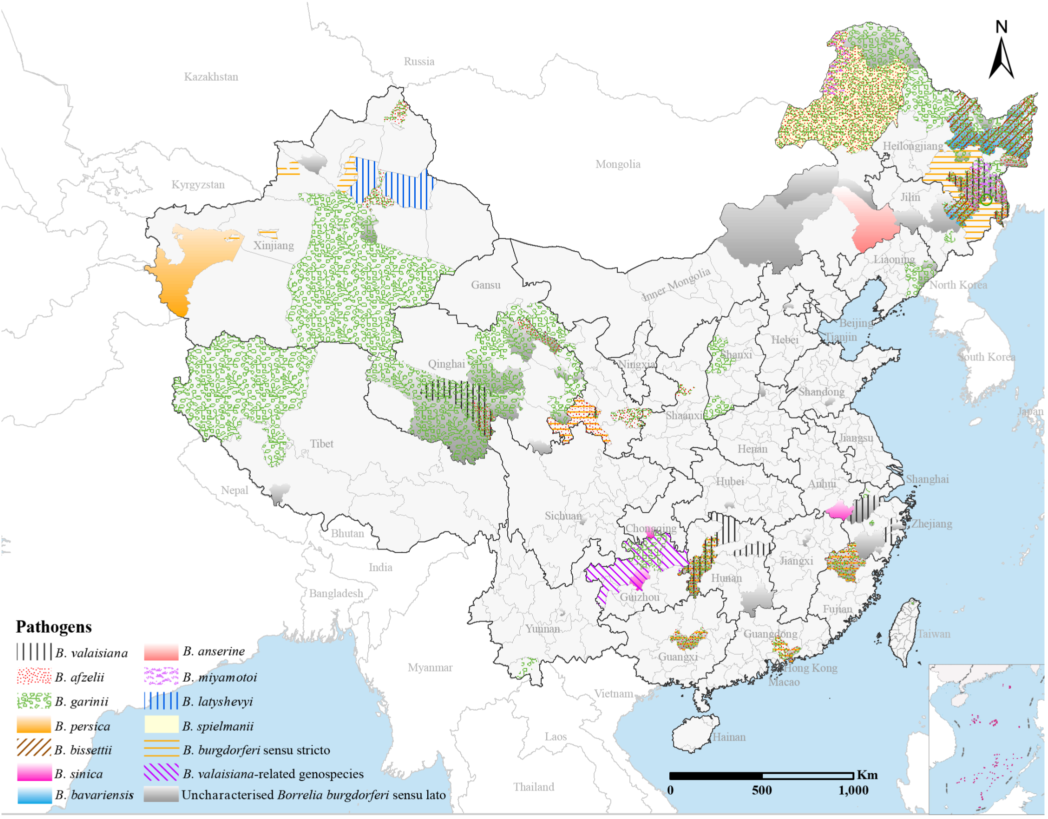


**Supplementary Fig. 37 The locations of species or subspecies of *Borrelia* detected in ticks during 1950–2018 in China.** Data were plotted at the county or prefecture level, depending on available resolution in the literature. Source data are provided as a Source Data file.

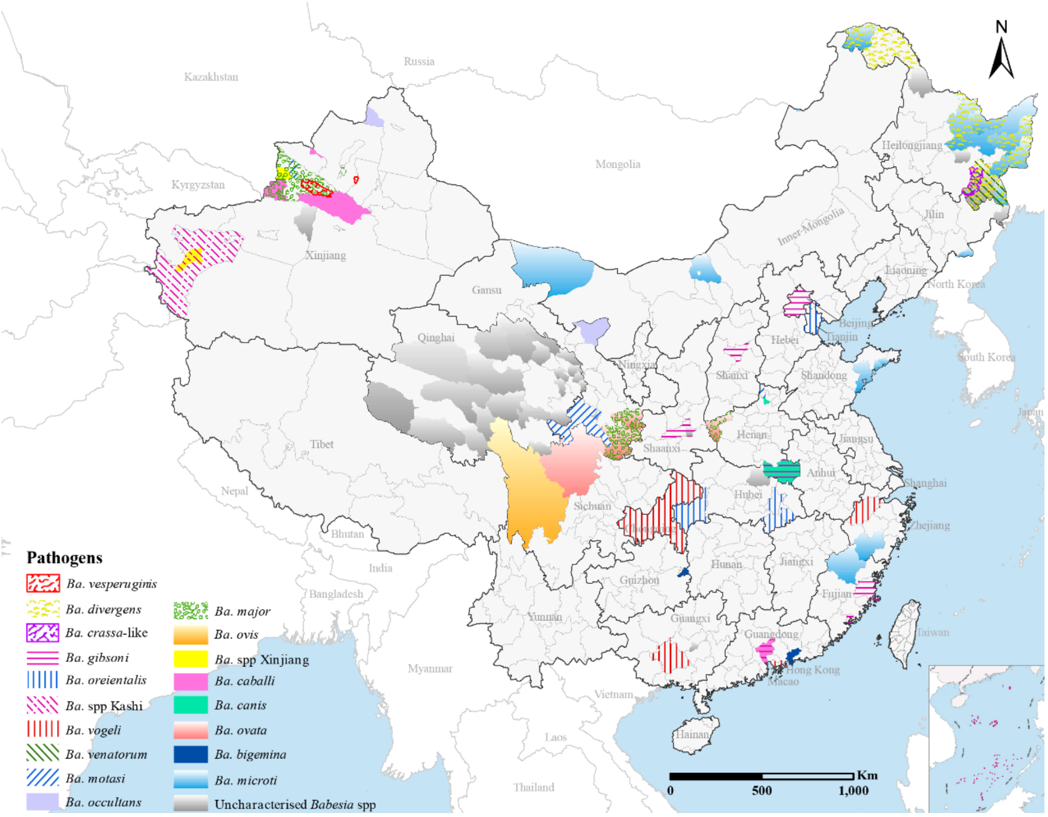


**Supplementary Fig. 38 The locations of species or subspecies of *Babesia* spp. detected in ticks during 1950–2018 in China.** Data were plotted at the county or prefecture level, depending on available resolution in the literature. Source data are provided as a Source Data file.

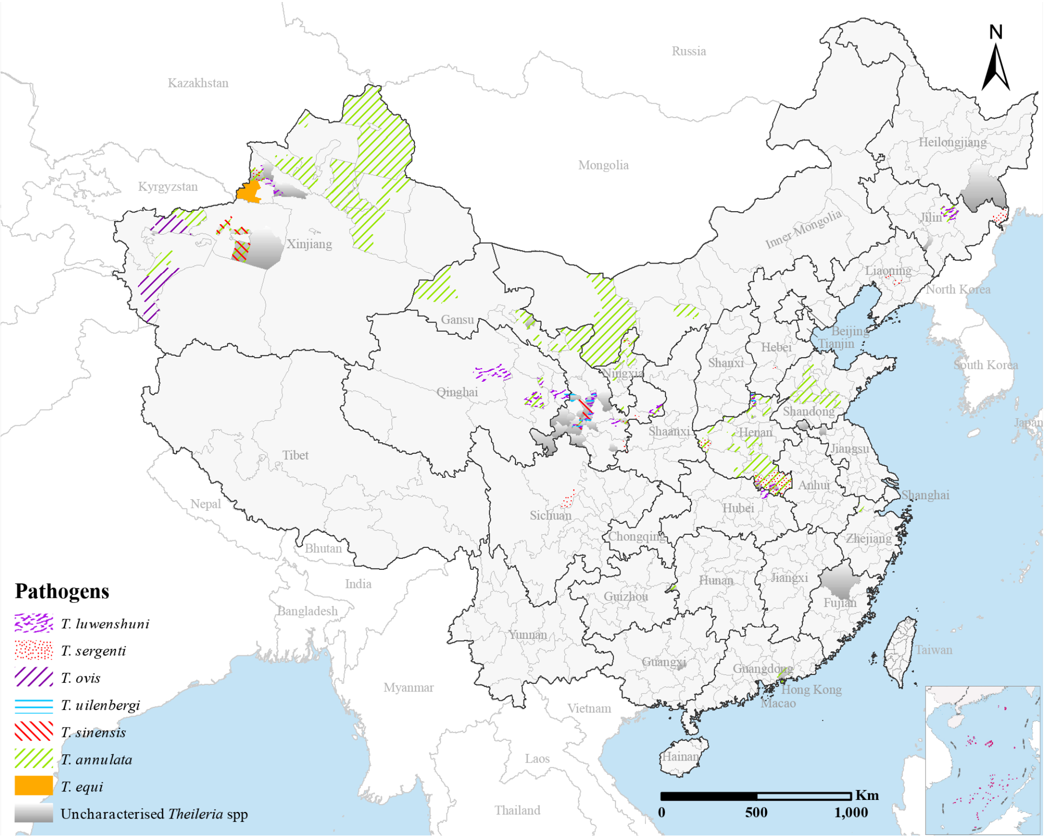


**Supplementary Fig. 39 The locations of species or subspecies of *Theileria* detected in ticks during 1950–2018 in China.** Data were plotted at the county or prefecture level, depending on available resolution in the literature. Source data are provided as a Source Data file.

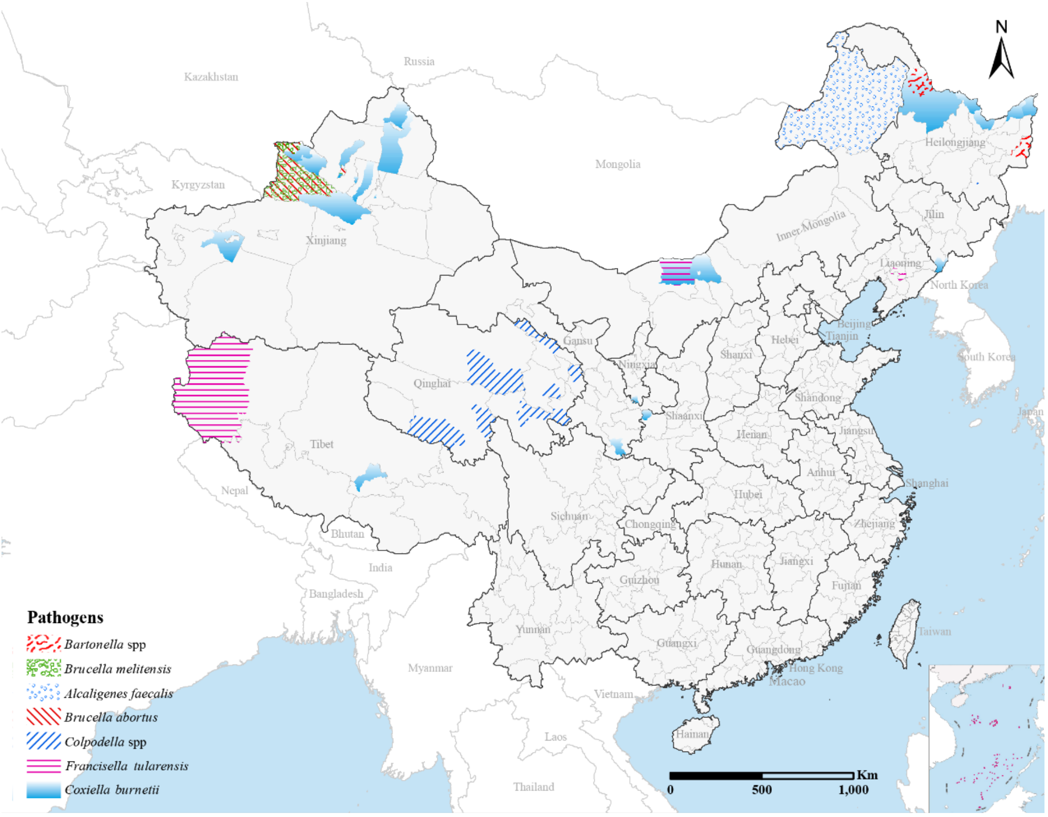


**Supplementary Fig. 40 The locations of other tick-borne bacteria detected in ticks during 1950–2018 in China.** Data were plotted at the county or prefecture level, depending on available resolution in the literature. Source data are provided as a Source Data file.

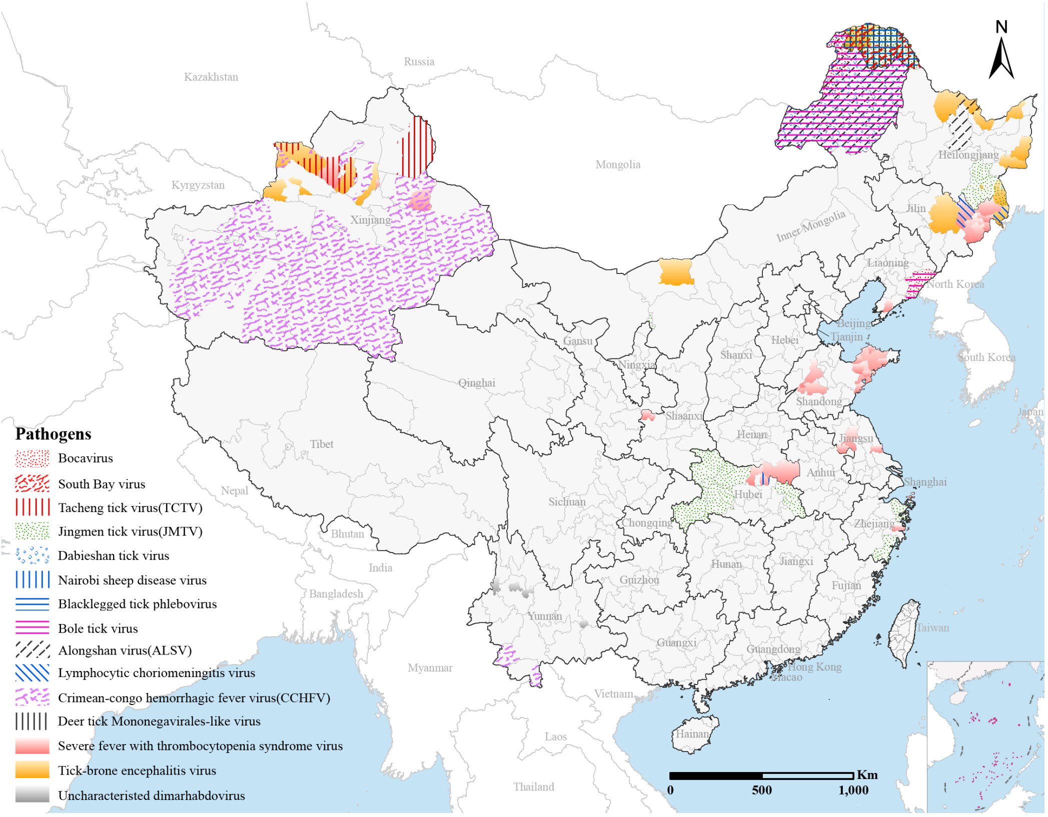


**Supplementary Fig. 41 The locations of tick-borne viruses detected in ticks during 1950–2018 in China.** Data were plotted at the county or prefecture level, depending on available resolution in the literature. Source data are provided as a Source Data file.

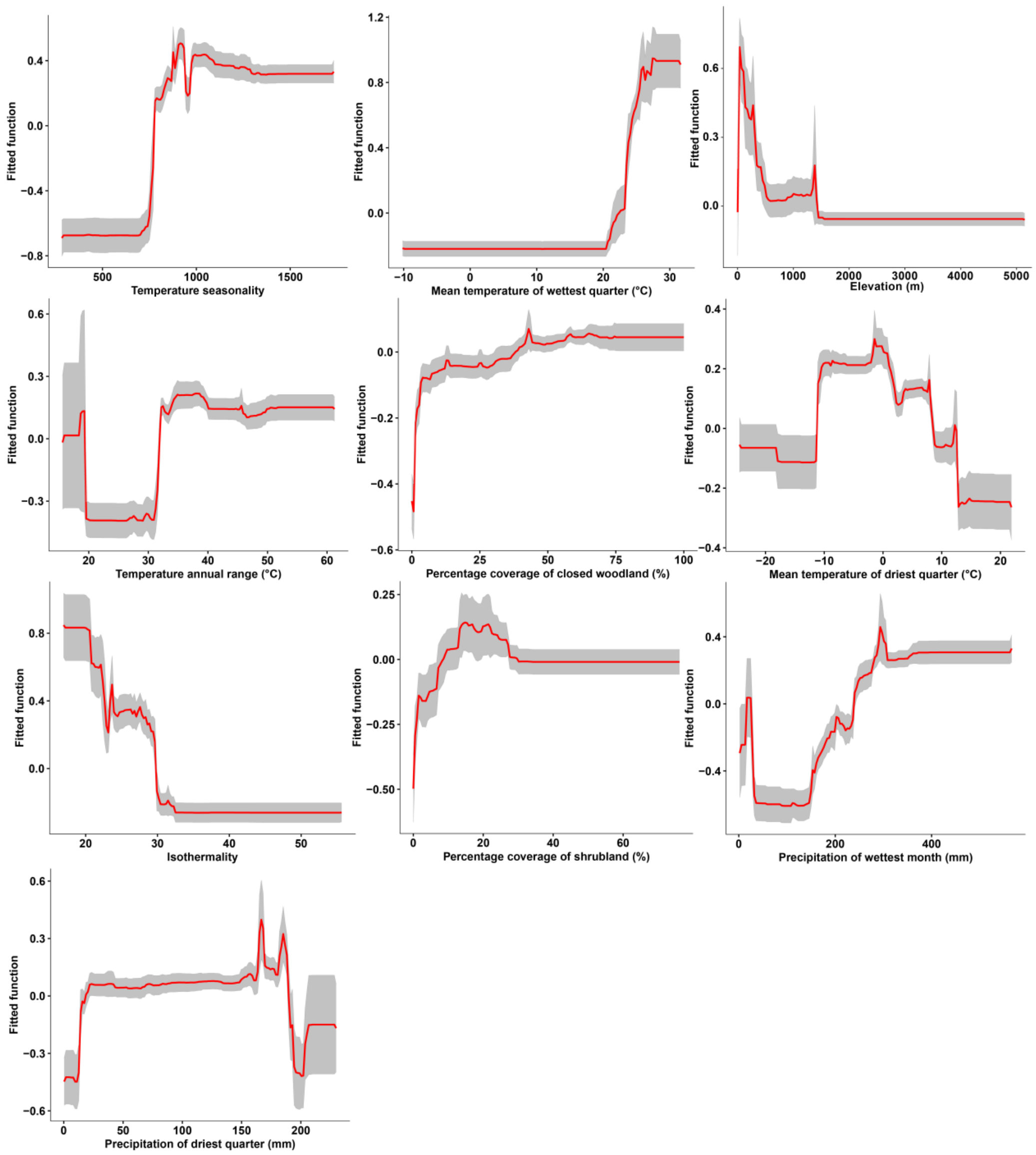


**Supplementary Fig. 42 The mean curves (red) and 95% percentiles (gray) for the effects of major predictors (RC≥5%) on the logit-transformed probability of occurrence of SFTSV based on the ensemble of BRT models.**

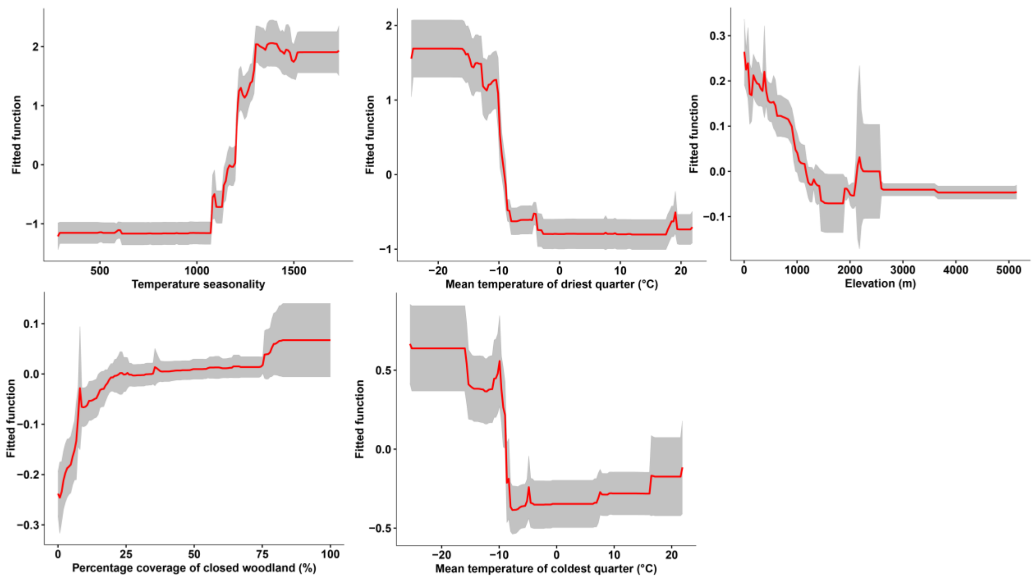


**Supplementary Fig. 43 The mean curves (red) and 95% percentiles (gray) for the effects of major predictors (RC≥5%) on the logit-transformed probability of occurrence of TBEV based on the ensemble of BRT models.**

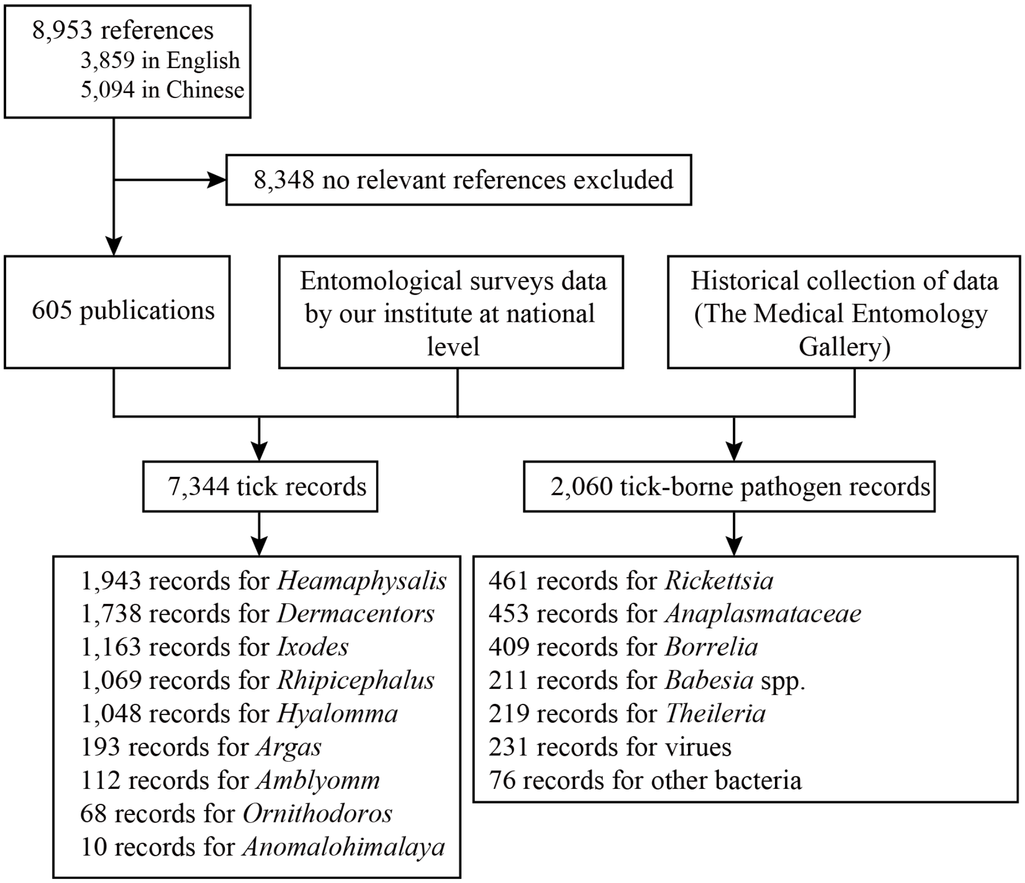


**Supplementary Fig. 44 The flow diagram of literature review.**

**Supplementary Table 1 BRT-model-estimated mean (standard deviation) relative contributions of top environmental and ecoclimatic factors (RC≥5%) to the spatial distribution of five most prevalent tick species in the Ixodes genus.** Mean AUCs (95% percentiles) and partial area AUC ratio (calculated at tolerance level of 0.2) are given.

| Category | Variable | *I. persulcatus* | *I. sinensis* | *I. granulatus* | *I. crenulatus* | *I. ovatus* |
|---|---|---|---|---|---|---|
| **Ecoclimatic** | Annual mean temp. | | | 18.23 (6.41) | | |
| | Isothermality | | | 6.59 (1.34) | | 7.23 (2.42) |
| | Temp. seasonality | 16.97 (4.39) | 5.34 (1.42) | | 5.23 (1.17) | 9.82 (3.20) |
| | Max temp. warmest month | | 6.68 (1.20) | | 6.69 (1.62) | |
| | Min temp. coldest month | | | 9.58 (3.40) | | |
| | Annual temp. range | | 5.75 (1.46) | 10.21 (3.02) | | 5.13 (1.57) |
| | Mean temp. driest quarter | 13.27 (4.79) | | 15.85 (5.42) | 6.42 (1.38) | |
| | Mean temp. warmest quarter | | 8.21 (1.73) | | | 5.45 (2.14) |
| | Mean temp. coldest quarter | 8.25 (3.84) | | 5.08 (2.87) | | |
| | Annual precip. | | | | 5.87 (1.80) | |
| | Precip. Wettest month | | | 5.43 (1.64) | 5.43 (2.42) | |
| | Precip. Driest month | 8.79 (2.61) | 7.63 (2.24) | | | |
| | Precip. Seasonality | | | | | 5.30 (1.87) |
| | Precip. Wettest quarter | | | | | 5.05 (1.62) |
| | Precip. Driest quarter | 6.54 (2.56) | 39.74 (6.37) | | | |
| | Precip. Coldest quarter | | 15.35 (4.02) | | | 5.92 (2.44) |
| **Environmental** | Mixed coniferous & broad leaved | 7.21 (2.32) | | | | 5.21 (1.58) |
| | Shrubland | | | | | 6.42 (1.73) |
| | Desert grassland | | | | 9.54 (3.47) | |
| | Alpine meadow steppe | | | | 9.83 (2.24) | |
| | Rainfed cropland | | | 5.25 (1.30) | | |
| | Urban construction land | | 7.08 (1.45) | | | |
| | Bare rock | | | | 18.32 (4.03) | |
| | Elevation | | | | 5.60 (1.32) | |
| | Relative humidity | | | | 7.25 (1.71) | |

| | | | | | | |
|---|---|---|---|---|---|---|
| **AUC** | Train | 0.986 (0.977-0.995) | 0.987 (0.980-0.993) | 0.991 (0.985-0.997) | 0.989 (0.981-0.997) | 0.979 (0.962-0.995) |
| | Test | 0.892 (0.855-0.929) | 0.924 (0.894-0.953) | 0.943 (0.920-0.965) | 0.918 (0.885-0.951) | 0.828 (0.778-0.878) |
| **Partial AUC Ratio** | Train | 1.79 | 1.81 | 1.84 | 1.87 | 1.87 |
| | Test | 1.54 | 1.65 | 1.71 | 1.68 | 1.47 |

**Supplementary Table 2 BRT-model-estimated mean (standard deviation) relative contributions of top environmental and ecoclimatic factors (RC≥5%) to the spatial distribution of four most prevalent tick species in the Haemaphysalis genus.** Mean AUCs (95% percentiles) and partial area AUC ratio (calculated at tolerance level of 0.2) are given.

| Category | Variable | *Ha. longicornis* | *Ha. concinna* | *Ha. japonica* | *Ha. hystricis* |
|---|---|---|---|---|---|
| **Ecoclimatic** | Annual mean temp. | 6.98 (1.13) | | | |
| | Mean diurnal range | | | | 6.82 (2.06) |
| | Isothermality | | | | 5.35 (1.94) |
| | Temp. seasonality | 9.43 (1.72) | 7.23 (2.41) | | 9.38 (2.87) |
| | Annual temp. range | 5.22 (1.15) | | | |
| | Mean temp. driest quarter | 7.09 (1.83) | 6.97 (2.65) | | |
| | Mean temp. coldest quarter | 5.64 (1.53) | | | |
| | Annual precip. | | | 8.02 (2.36) | 5.51 (2.89) |
| | Precip. seasonality | | | | 6.13 (1.82) |
| | Precip. wettest quarter | | | | 7.62 (3.50) |
| | Precip. coldest quarter | | | | 5.97 (2.71) |
| **Environmental** | Coniferous | | 9.61 (2.43) | | 5.05 (1.31) |
| | Broad leaved | | | 5.81 (1.61) | 6.44 (2.73) |
| | Mixed coniferous & broad leaved | | 6.74 (2.00) | 17.19 (2.28) | 9.85 (2.32) |
| | Meadow | | | 9.10 (1.83) | |
| | Shrub grassland | 23.25 (2.29) | | | |
| | Irrigated cropland | 5.66 (1.21) | | | |
| | Rainfed cropland | 8.01 (0.98) | 5.92 (1.43) | 13.15 (2.21) | |
| | Rural settlement | 5.83 (1.26) | | 5.51 (1.80) | |
| | Inland water body | | 5.41 (1.74) | | |
| | Elevation | 5.24 (0.91) | | | |
| | Relative humidity | 6.12 (0.93) | | 5.92 (1.89) | 5.99 (2.30) |
| **AUC** | Train | 0.979 (0.972-0.986) | 0.995 (0.988-0.999) | 0.994 (0.988-0.999) | 0.998 (0.996-0.999) |
| | Test | 0.900 (0.879-0.920) | 0.875 (0.827-0.922) | 0.888 (0.835-0.941) | 0.893 (0.854-0.933) |
| **Partial AUC Ratio** | Train | 1.59 | 1.90 | 1.92 | 1.93 |

| | | | | |
|---|---|---|---|---|
| Test | 1.45 | 1.54 | 1.59 | 1.59 |

**Supplementary Table 3 BRT-model-estimated mean (standard deviation) relative contributions of top environmental and ecoclimatic factors (RC≥5%) to the spatial distribution of three most prevalent tick species in the Rhipicephalus genus.** Mean AUCs (95% percentiles) and partial area AUC ratio (calculated at tolerance level of 0.2) are given.

| Category | Variable | *R. sanguineus* | *R. microplus* | *R. haemaphysaloides* |
|---|---|---|---|---|
| **Ecoclimatic** | Mean diurnal range | | | 6.51 (1.34) |
| | Isothermality | | 5.27 (1.53) | |
| | Temp. seasonality | 6.41 (1.14) | 12.88 (3.52) | 8.11 (2.93) |
| | Min temp. coldest month | | 5.67 (1.99) | 7.98 (1.82) |
| | Annual temp. range | | 11.55 (2.74) | 5.35 (1.24) |
| | Mean temp. driest quarter | 8.32 (1.28) | 5.74 (2.17) | 5.56 (2.43) |
| | Mean temp. warmest quarter | | | 7.08 (1.93) |
| | Mean temp. coldest quarter | | 6.79 (1.83) | |
| | Annual precip. | | 5.66 (1.28) | 5.16 (1.38) |
| | Precip. wettest month | 5.28 (1.23) | | |
| | Precip. driest month | 7.72 (1.17) | | |
| | Precip. seasonality | 5.25 (1.18) | 5.45 (1.22) | |
| | Precip. driest quarter | | | 7.43 (2.11) |
| **Environmental** | Coniferous | 6.05 (1.42) | | 5.04 (1.52) |
| | Shrub grassland | 13.61 (1.38) | | |
| | Rainfed cropland | 6.09 (0.96) | | |
| | Elevation | 5.80 (1.01) | 5.18 (1.02) | |
| **AUC** | Train | 0.983 (0.975-0.991) | 0.970 (0.987-0.998) | 0.989 (0.978-0.999) |
| | Test | 0.833 (0.800-0.866) | 0.871 (0.844-0.898) | 0.901 (0.862-0.940) |
| **Partial AUC Ratio** | Train | 1.69 | 1.70 | 1.87 |
| | Test | 1.30 | 1.47 | 1.58 |

**Supplementary Table 4 BRT-model-estimated mean (standard deviation) relative contributions of major environmental and ecoclimatic factors (RC≥5%) to the spatial distribution of four most prevalent tick species in the Demacentor genus.** Mean AUCs (95% percentiles) and partial area AUC ratio (calculated at tolerance level of 0.2) are given.

| **Category** | **Variable** | ***D. nuttalli*** | ***D. silvarum*** | ***D. daghestanicus*** | ***D. marginatus*** |
|---|---|---|---|---|---|
| **Ecoclimatic** | Temp. seasonality | 9.38 (1.18) | 14.57 (3.50) | | |
| | Annual temp. range | | 11.39 (2.57) | | 5.01 (1.84) |
| | Mean temp. wettest quarter | 6.70 (1.27) | 5.86 (0.94) | | |
| | Mean temp. driest quarter | | 5.85 (1.52) | | 5.68 (1.53) |
| | Mean temp. warmest quarter | | | | 5.21 (1.12) |
| | Annual Precip. | | 8.06 (1.22) | | |
| | Precip. wettest month | 44.32 (3.31) | | | 5.09 (1.77) |
| | Precip. driest month | 6.67 (1.22) | 9.79 (2.47) | | 6.58 (1.80) |
| | Precip. seasonality | | | | 7.61 (2.23) |
| | Precip. wettest quarter | | | | |
| | Precip. driest quarter | | | | |
| | Precip. warmest quarter | | | 18.69 (4.47) | |
| **Environmental** | Coniferous | | | 5.42 (2.29) | |
| | Meadow | | | 7.24 (2.69) | 8.13 (1.94) |
| | Desert grassland | | | 22.06 (5.41) | |
| | Shrub grassland | 5.92 (0.74) | 8.80 (1.01) | | |
| | Rainfed cropland | 5.00 (1.10) | | | 7.45 (1.08) |
| | Rural settlement | | | | 6.09 (1.72) |
| | Inland water body | | | 5.75 (1.22) | |
| | Elevation | | | | |
| **AUC** | Train | 0.998 (0.997-0.999) | 0.993 (0.987-0.998) | 0.994 (0.987-0.999) | 0.999(0.998-0.999) |
| | Test | 0.966 (0.956-0.975) | 0.926 (0.907-0.944) | 0.930 (0.882-0.977) | 0.960 (0.942-0.978) |
| **Partial AUC Ratio** | Train | 1.56 | 1.66 | 1.90 | 1.91 |
| | Test | 1.50 | 1.52 | 1.72 | 1.78 |

**Supplementary Table 5 BRT-model-estimated mean (standard deviation) relative contributions of major environmental and ecoclimatic factors (RC≥5%) to the spatial distribution of two most prevalent tick species in the Hyalomma genus and one most prevalent tick specie in the Argas genus.** Mean AUCs (95% percentiles) and partial area AUC ratio (calculated at tolerance level of 0.2) are given.

| Category | Variable | *Hy. scupense* | *Hy. asiaticum* | *Ar. persicus* |
|---|---|---|---|---|
| **Ecoclimatic** | Temp. seasonality | 7.18 (1.30) | 6.67 (1.64) | |
| | Mean temp. driest quarter | 10.21 (1.35) | | 5.42 (2.97) |
| | Annual precip. | | | 7.84 (2.79) |
| | Precip. wettest month | | 9.06 (3.86) | |
| | Precip. driest month | 6.52 (1.37) | | 6.08 (1.49) |
| | Precip. seasonality | 7.45 (1.60) | | |
| | Precip. wettest quarter | 6.71 (1.09) | 23.08 (6.78) | |
| | Precip. warmest quarter | | 20.02 (7.62) | |
| **Environmental** | Desert grassland | | 7.31 (2.20) | 7.29 (2.72) |
| | Shrub grassland | 12.79 (1.62) | | |
| | Irrigated cropland | 6.08 (0.96) | | 10.72 (2.17) |
| | Rainfed cropland | | 5.32 (0.92) | |
| | Rural settlement | | 7.12 (1.23) | |
| | Bare rock | | | 5.06 (2.14) |
| | Elevation | 9.52 (1.53) | 6.03 (1.05) | 12.83 (2.89) |
| **AUC** | Train | 0.993 (0.987-0.998) | 0.993 (0.987-0.998) | 0.988 (0.976-0.999) |
| | Test | 0.912 (0.893-0.932) | 0.948 (0.926-0.969) | 0.874 (0.836-0.902) |
| **Partial AUC Ratio** | Train | 1.70 | 1.81 | 1.88 |
| | Test | 1.50 | 1.67 | 1.49 |

**Supplementary Table 6 The specific references for all 124 tick species in China from 1950 to 2018**

| Tick species | Reference |
|---|---|
| *Ixodes* | 1-154 |
| *I. persulcatus* | 2-5,9-14,19,21,23-26,28,29,31-34,36-38,40,42-46,49-51,53,55,57-63,65-71,73-79,81,82,99,104,105,108,110,111,113,115-132,134,136,137,140,142-146,148-154 |
| *I. sinensis* | 5,8,12,15-19,21-23,27,28,39,41,43,49,80,83,85,90,93,94,96,98,106,107,133,139,140 |
| *I. granulatus* | 5,17,20,23,39,43,48,56,86,88,91,92,97,102,107,135,138,140,147 |
| *I. crenulatus* | 5,47,87,89,95,101,103,140,144-146 |
| *I. ovatus* | 5,7,29,35,39,41,48,54,64,87,91,97,102,109,114,135,139,140,147 |
| *I. acutitarsus* | 5,35,48,49,64,84,135,140,144 |
| *I. simplex* | 5,141 |
| *I. spinicoxalis* | 5,109 |
| *I. nuttallianus* | 5,35,64,109,140 |
| *I. hyatti* | 5,49,84,112,126,135 |
| *I. pomerantzevi* | 5,49,84,126,140 |
| *I. vespertilionis* | 5,35,37,112 |
| *I. kuntzi* | 49,84,135 |
| *I. moschiferi* | 87 |
| *I. acuminatus* | 49,84,100,101 |
| *I. arboricola* | 5,47,52,140,145 |
| *I. moscharius* | 5 |
| *I. tanuki* | 5 |
| *I. kazakstani* | 6 |
| *I. kaschmiricus* | 5 |
| *I. pavlovskyi* | 30 |
| *I. nipponensis* | 30,148 |
| *I. berlesei* | 6 |
| *I. kangdingensis* | 1 |
| *I. myospalacis* | 5,72 |
| *I. semenovi* | 5 |
| *Haemaphysalis* | 2-5,15-18,23,25,27-29,35-37,39,41-43,45-49,52,53,55,57-59,62-69,72,74,77,80-82,84-88,90-92,94,96,98-104,106-112,116,121-127,129,131-135,137-140,143-149,151-279 |
| *Ha. longicornis* | 3,5,17,18,23,27,37,42,43,49,53,77,80-82,84,85,92,96,98,102,103,106-108,110,112,124,126,135,140,143,146,148,149,153,155,158-161,164,166,167,173,174,176-182,188,189,192,193,202-204,206,208,210,212,213,216-219,221-223,226,228,230,235,237-240,244,250,255-259,261,262,265,266,271,273,274,278,279 |
| *Ha. concinna* | 2,4,5,15,16,27,28,36,42,43,45,46,49,55,57-59,62,63,65-69,72,74,81,84,99,104,108,112,116,121-123,125,127,129,131,132,134,135,137,140,143,145-148,151,152,154,156,163,168,169,172,185,190-192,205,227,229,247-249,252,256,275-277 |
| *Ha. japonica* | 5,37,46,49,57-59,62,63,65-67,72,74,84,87,99,121,123,125,134,135,140,143,146 |

| | |
|---|---|
| | ,148,185,227,251 |
| *Ha. hystricis* | 5,17,35,39,49,84,92,94,102,107,135,139,140,175,179,217 |
| *Ha. lagrangei* | 5,39,49,53,84,92,133,135,140,178,215,217,220,272 |
| *Ha. punctata* | 5,101,111,129,140,144,145,148,164,169,195,198,199,201,249,253,277 |
| *Ha. verticalis* | 5,37,49,69,84,135,140,146,211,246,254,268 |
| *Ha. qinghaiensis* | 5,72,144,149,170,171,173,183,184,192,196,202,207,224,227,230-234,236,237,239-242,245,270 |
| *Ha. campanulata* | 5,37,39,49,84,90,107,135,143,151,154,192,200,246,261,274 |
| *Ha. sulcata* | 5,47,52,101,111,145,197,199,243 |
| *Ha. taiwana* | 5,35,39,90,143,179,214,217,264,269 |
| *Ha. doenitzi* | 5,15,39,86,92,94,106,107,174,217,278 |
| *Ha. pospelovashtromae* | 47,52,101,111,195,267 |
| *Ha. flava* | 5,29,35,48,49,84,106,135,140,147,148,157,190,192,205,278 |
| *Ha. yeni* | 5,17,35,39,41,49,84,90,107,135,138,139,162,209,260,263 |
| *Ha. montgomeryi* | 5,48,91,147,225 |
| *Ha. formosensis* | 107 |
| *Ha. aponommoides* | 5,39,64,217 |
| *Ha. moschisuga* | 5,270 |
| *Ha. kitaokai* | 5,35,102 |
| *Ha. spinigera* | 5,35,39,49,84,135 |
| *Ha. tibetensis* | 5,109 |
| *Ha. ornithophila* | 5,144,147,264 |
| *Ha. erinacei* | 5,100,111,143,194 |
| *Ha. warburtoni* | 5,108,109,112 |
| *Ha. wellingtoni* | 5,39 |
| *Ha. birmaniae* | 5,35,264 |
| *Ha. goral* | 5 |
| *Ha. nepalensis* | 5,49,64,84,135,214 |
| *Ha. megaspinosa* | 5,48,148 |
| *Ha. quadriaculeata* | 186 |
| *Ha. asiatica* | 5,35 |
| *Ha. canestrinii* | 5 |
| *Ha. primitiva* | 5,269 |
| *Ha. menglaensis* | 5 |
| *Ha. mageshimaensis* | 5,147 |
| *Ha. aborensis* | 5 |
| *Ha. sinensis* | 5,84,135 |
| *Ha. bandicota* | 5,25,88 |
| *Ha. colasbelcouri* | 91,147 |
| *Ha. heinrichi* | 165 |
| *Ha. megalaimae* | 187 |
| *Amblyomma* | 5,17,30,35,39,41,80,85,90,92,94,107,143,162,175,217,264,280,281 |
| *Am. testudinarium* | 5,17,35,39,41,80,85,90,92,94,107,143,162 |
| *Am. javanense* | 5,35,92,107,217 |
| *Am. crassipes* | 5,35,264 |
| *Am. varanense* | 5,35,217,280 |
| *Am. helvolum* | 5,175,281 |

| | |
|---|---|
| *Am. pattoni* | 107 |
| *Am. cordiferum* | 30 |
| *Am. geoemydae* | 5 |
| *Am. breviscutatum* | 30 |
| *Am. pseudolaeve* | 5 |
| *Rhipicephalus* | 5-7,17,25,35,37,39,43,47,49,52,64,77,80,84,85,87,88,92,96,98,100,102,103,106-109,111,112,129,133,135,140,143-147,149,164,169,173,178-180,182,190,192,194,195,197,199,201,205,207,213,214,237,246,253,255,257,261,263,264,274,276-279,282-313 |
| *R. sanguineus* | 5,7,25,35,37,47,77,80,85,88,92,100,103,111,129,140,143,145,149,164,173,179,182,190,194,205,207,213,237,246,255,257,261,274,278,279,283-285,287,289,292,293,296,297,305,307,310-312 |
| *R. microplus* | 5,17,35,37,39,43,49,64,84,85,87,92,102,106-109,112,129,133,135,140,143,144,146,147,149,164,178-180,182,192,207,214,246,261,263,264,276,279,285,288,290,293-295,297,298,300,303,305,306,308,309 |
| *R. haemaphysaloides* | 5,17,35,39,49,64,84,85,92,96,98,103,107,129,133,179,180,282,285,291,293 |
| *R. pumilio* | 5,47,100,111,140,145,195,197,199,201,264,292,293,301,302,304 |
| *R. turanicus* | 5,52,111,140,143,145,169,253,277,286,292,299,301,302,313 |
| *R. schulzei* | 111,195 |
| *R. bursa* | 5,140,144 |
| *R. rossicus* | 6 |
| *Dermacentor* | 2-5,14,15,17,19,20,27,28,36,37,39,42,43,45-47,52,53,55,57-59,62-69,71,72,74,80,82,85,87,89,92,99,100,102,103,106,107,109,111,113,116-118,121,123,125-129,131,132,134,137,139,140,142-146,148,150-154,163,164,167,169,185,190,192,194,195,197-199,201,205,211,213,217,227,236,237,240,249,252-256,263,274,277,292,302,304,310,313-340 |
| *D. nuttalli* | 5,15,19,27,28,42,43,57,69,72,87,109,111,129,140,144-146,150,151,153,154,167,169,192,195,197-199,201,213,227,236,237,240,255,277,304,310,315,317,324,326,328,330-333,335,336,338,339 |
| *D. silvarum* | 2-5,14,19,20,27,36,37,39,42,43,45,46,53,55,58,59,62,63,65-69,71,72,74,82,99,103,109,111,113,116-118,121,123,125-129,131,132,134,137,139,140,142-146,148,151-154,163,164,185,192,195,198,199,213,227,236,252,254-256,313,316,319,321,326,327,334,337-340 |
| *D. daghestanicus* | 5,47,52,72,100,111,195,197,199,201,211,227,237,292,304,325-327,333,336 |
| *D. marginatus* | 5,111,144,145,150,151,154,164,169,194,195,198,199,201,249,253,255,277,302,320,334 |
| *D. reticulatus* | 5,64,143,195,255 |
| *D. abaensis* | 5,87,109,111,140,144,192,195,199 |
| *D. pavlovskyi* | 5,47,52,111,145,327,339 |
| *D. auratus* | 5,39,80,85,92,140,217,263 |
| *D. sinicus* | 5,72,106,129,140,143,190,192,205,274,318,323 |
| *D. everestianus* | 5,89,329 |
| *D. taiwanensis* | 5,17,102 |
| *D. steini* | 107 |
| *D. montanus* | 5,52,327,336 |
| *D. compactus* | 322 |
| *D. raskemensis* | 314 |

| | |
|---|---|
| *Hyalomma* | 5,17,35,37,47,52,53,72,77,80,81,85,88,92,100,103,106,111,129,135,140,143-145,151,154,164,169,179,180,182,190,194,195,197-199,201,202,205,207,211,213,227,233,237,240,246,249,253-255,257,274,277,279,283-285,287,288,292,293,296,297,301,302,304,305,307,310,313,315,318,323,325,326,332,333,336,341-358 |
| *Hy. scupense* | 5,37,47,52,53,72,100,106,111,129,135,140,143-145,151,154,164,169,180,195,197,199,201,202,227,237,240,249,277,279,288,345,353,356-358 |
| *Hy. asiaticum* | 5,17,35,37,47,77,80,81,85,88,92,100,103,111,129,140,143-145,164,169,179,182,190,194,195,197-199,201,205,207,211,213,237,246,249,253-255,257,274,277,283-285,287,292,293,296,297,301,302,304,305,307,310,313,315,318,325,326,332,333,336,341,342,346-348,350-353,355,359 |
| *Hy. anatolicum* | 5,47,111,144,145,164,195,199,240,279,288,292,333,336,343,349,353,354 |
| *Hy. marginatum* | 5,111,195,233,323,333,353,357 |
| *Hy. dromedarii* | 5,47,52,143,197,353 |
| *Hy. turanicum* | 344 |
| *Hy. isaaci* | 5 |
| *Anomalohimalaya* | 5,6,47,360 |
| *An. cricetuli* | 5,47,360 |
| *An. lama* | 5 |
| *An. lotozkyi* | 6 |
| *Ornithodoros* | 30,47,52,140,143,144,196,279,361-364 |
| *O. tartakovskyi* | 143,362 |
| *O. papillipes* | 47,143,196,362 |
| *O. lahorensis* | 47,52,140,143,144,196,279,361,363,364 |
| *O. capensis* | 30 |
| *Argas* | 37,47,135,140,143,144,150,178,365-370 |
| *Ar. persicus* | 37,47,135,140,143,144,178,366-368 |
| *Ar. vulgaris* | 140,178 |
| *Ar. vespertilionis* | 135 |
| *Ar. robertsi* | 369 |
| *Ar. beijingensis* | 369 |
| *Ar. japonicus* | 150,369 |
| *Ar. assimilis* | 370 |
| *Ar. pusillus* | 369 |
| *Ar. sinensis* | 365 |

**Supplementary Table 7 The social, environmental and ecoclimatic variables used for ecological modeling for tick species and tick-borne pathogens at the county level in this study.**

| Category | Variable | Description |
|---|---|---|
| Ecoclimatic | BIO01* | Annual mean temperature (℃) |
| | BIO02 | Mean diurnal range (Mean of monthly (max temp-min temp)) (℃) |
| | BIO03 | Isothermality (BIO02/BIO07)(*100) |
| | BIO04 | Temperature seasonality (standard deviation*100) |
| | BIO05 | Max temperature of warmest month (℃) |
| | BIO06* | Min temperature of coldest month (℃) |
| | BIO07 | Annual range of temperature (BIO05-BIO06) (℃) |
| | BIO08* | Mean temperature of wettest quarter (℃) |
| | BIO09* | Mean temperature of driest quarter (℃) |
| | BIO10 | Mean temperature of warmest quarter (℃) |
| | BIO11* | Mean temperature of coldest quarter (℃) |
| | BIO12* | Annual precipitation (mm) |
| | BIO13* | Precipitation of wettest month (mm) |
| | BIO14 | Precipitation of driest month (mm) |
| | BIO15* | Precipitation seasonality(Coefficient of variation) |
| | BIO16 | Precipitation of wettest quarter (mm) |
| | BIO17* | Precipitation of driest quarter (mm) |
| | BIO18* | Precipitation of warmest quarter (mm) |
| | BIO19 | Precipitation of coldest quarter (mm) |
| Environmental | Coniferous* | Percentage coverage of coniferous (1%) |
| | Broad leaved | Percentage coverage of broad leaved (1%) |
| | Mixed coniferous and broad leaved | Percentage coverage of mixed coniferous and broad leaved (1%) |
| | Shrubland* | Percentage coverage of shrubland (1%) |
| | Meadow* | Percentage coverage of meadow (1%) |
| | Desert grassland* | Percentage coverage of desert grassland (1%) |
| | Typical grassland | Percentage coverage of typical grassland (1%) |
| | Alpine meadow steppe* | Percentage coverage of alpine meadow steppe (1%) |
| | Shrub grassland* | Percentage coverage of shrub grassland (1%) |
| | Paddy field | Percentage coverage of paddy field (1%) |
| | Irrigated cropland* | Percentage coverage of irrigated cropland (1%) |
| | Rainfed cropland* | Percentage coverage of rainfed cropland (1%) |
| | Urban construction land* | Percentage coverage of urban construction land (1%) |
| | Rural settlement* | Percentage coverage of rural settlement (1%) |
| | Swamp | Percentage coverage of swamp (1%) |

| | | |
|---|---|---|
| | Coastal wetlands | Percentage coverage of coastal wetlands (1%) |
| | Inland water body* | Percentage coverage of inland water body (1%) |
| | River and lake beach land | Percentage coverage of river and lake beach land (1%) |
| | Ice and snow | Percentage coverage of ice and snow (1%) |
| | Bare rock | Percentage coverage of bare rock (1%) |
| | Bare land | Percentage coverage of bare land (1%) |
| | Desert | Percentage coverage of desert (1%) |
| | Elevation* | Average elevation (m) |
| | Relative humidity | Annually average relative humidity (1%) |
| Social | Proportion of rural population | # of rural population / # of total population |
| | Density of rural population | # of rural population / total area |

* Used as predictors in the logistic model for selection of counties for tick survey. The output sampling probabilities of counties (after taking reciprocal) were used as weights for the BRT models for the 19 tick species.

## Supplementary References


1 Guo, T., Sun, Y., Xu, G. & Durden, L. A. *Ixodes kangdingensis* (Acari: Ixodidae), a new species from the Siberian weasel, *Mustela sibirica* (Carnivora: Mustelidae) in China. *Parasitology Open* **3, e7** (2017).

2 Li, H. *Investigations on natural foci and pathogenic characteristics of two vector-borne diseases*, Ph.D Dissertation. The Academy of Military Medical Sciences, (2013).

3 Li, Y. *Investigation on the infection with Anaplasma phagocytophilum in ticks from forest area in Northeast of China*, Master Dissertation. Suzhou University, (2012).

4 Tang, H. *An investigation of carried status of three pathogens in ticks and mice in forest region of Heilongjiang*, Master Dissertation. Central South University, (2012).

5 Teng, K. F. & Jiang, Z. J. *Economic insect fauna of China. fasc 39 Acari: Ixodidae*. 1–359 (Beijing, Science Press, 1991).

6 Yu, X., Ye, R. Y. & Gong, Z. D. *Xinjiang tick genus*. (Urumchi, Xinjiang Science and Technology Medical Publishing House, 1997).

7 Chao, L. L., Liao, H. T., Ho, T. Y. & Shih, C. M. First detection and molecular identification of *Babesia gibsoni* from *Rhipicephalus sanguineus* ticks. *Acta Trop* **166**, 356–362 (2017).

8 Hou, J. et al. Prevalence of *Borrelia burgdorferi* Sensu Lato in Ticks from Eastern China. *Am J Trop Med Hyg* **92**, 262–266 (2015).

9 Tsai, K. H. et al. Isolation and identification of a novel spotted fever group rickettsia, strain IG-1, from *Ixodes granulatus* ticks collected on Orchid Island (Lanyu), Taiwan. *Am J Trop Med Hyg* **79**, 256–261 (2008).

10 Ai, C. X. et al. Clinical manifestations and epidemiological characteristics of Lyme disease in Hailin County, Heilongjiang Province, China. *Ann N Y Acad Sci* **539**, 302–313 (1988).

11 Li, M. Q., T. M., Nobuhiro, T., Fubito, I., Hiromi, F. Lyme disease *Borrelia* species in northeastern China resemble those isolated from Far Eastern Russia and Japan. *Appl Environ Microbiol* **64**, 2705–2709 (1998).

12 Takada, N. et al. Lyme disease *Borrelia* spp. in ticks and rodents from northwestern China. *Appl Environ Microbiol* **67**, 5161–5165 (2001).

13 Liu, S., C. Y., Cui, Y. F., Li, B. X., Wu, L. J., Liu, Y. Investigation of *Borrelia* spp. in ticks (Acari: Ixodidae) at the border crossings between China and Russia in Heilongjiang Province, China. *Asian Pac J Trop Med* **5**, 459–464 (2012).

14 Zhang, F. et al. Detection of *Francisella tularensis* in ticks and identification of their genotypes using multiple-locus variable-number tandem repeat analysis. *BMC Microbiol* **8**, 152 (2008).

15 Jiang, J. F. et al. *Anaplasma phagocytophilum* infection in ticks, China–Russia border. *Emerg Infect Dis* **17**, 932–934 (2011).

16 Li, H. et al. Human infection with *Candidatus* Neoehrlichia mikurensis, China. *Emerg Infect Dis* **18**, 1636–1639 (2012).

17 Sun, J. et al. Detection of spotted fever group rickettsiae in ticks from Zhejiang Province, China. *Exp Appl Acarol* **65**, 403–411 (2015).

18 Wang, T. et al. Epidemiological characteristics and environmental risk factors of severe fever with thrombocytopenia syndrome in Hubei Province, China, from 2011 to 2016. *Front Microbiol* **8**, 387 (2017).

19 Wei, F. et al. Molecular detection and characterization of zoonotic and veterinary pathogens in ticks from northeastern China. *Front Microbiol* **7**, 1913 (2016).

20 Masuzawa, T. et al. *Borrelia sinica* sp. nov., a lyme disease-related *Borrelia* species isolated in China. *Int J Syst Evol Microbiol* **51**, 1817–1824 (2001).

21 Jiang, B. et al. Genome sequence of *Borrelia garinii* strain NMJW1, isolated from China. *J Bacteriol* **194**, 6660–6661 (2012).

22 Cao, W. C. et al. *Granulocytic Ehrlichiae* in *Ixodes persulcatus* ticks from an area in China where Lyme disease is endemic. *J Clin Microbiol* **38**, 4208–4210 (2000).

23 Chu, C. Y. et al. Novel genospecies of *Borrelia burgdorferi* sensu lato from rodents and ticks in southwestern China. *J Clin Microbiol* **46**, 3130–3133 (2008).

24 Hao, Q., Hou, X., Geng, Z. & Wan, K. Distribution of *Borrelia burgdorferi* sensu lato in China. *J Clin Microbiol* **49**, 647–650 (2011).

25 Kuo, C. C. et al. Widespread *Rickettsia* spp. Infections in Ticks (Acari: Ixodoidea) in Taiwan. *J Med Entomol* **52**, 1096–1102 (2015).

26 Chao, L. L., Liu, L. L. & Shih, C. M. Prevalence and molecular identification of *Borrelia* spirochetes in *Ixodes granulatus* ticks collected from *Rattus losea* on Kinmen Island of Taiwan. *Parasit Vectors* **5**, 167 (2012).

27 Liu, H. et al. Characterization of *Rickettsiae* in ticks in northeastern China. *Parasit Vectors* **9**, 498 (2016).

28 Zhou, X. et al. Human babesiosis, an emerging tick-borne disease in the People's Republic of China. *Parasit Vectors* **7**, 509 (2014).

29 Qiu, Y., Nakao, R., Ohnuma, A., Kawamori, F. & Sugimoto, C. Microbial population analysis of the salivary glands of Ticks; A possible strategy for the surveillance of bacterial pathogens. *PloS One* **9**, e103961 (2014).

30 Robbins, R. G. The ticks (Acari: Ixodida: Argasidae, Ixodidae) of Taiwan: a synonymic checklist. *Proc Entomol Soc Wash.* **107**, 245–253 (2005).

31 Zhang, X. C. et al. The composition and transmission of microbiome in hard tick, *Ixodes persulcatus*, during blood meal. *Ticks Tick Borne Dis* **5**, 864–870 (2014).

32 Chu, C. Y., Jiang, B. J., He, J., Gao, Y., Zhang, P. H., Wu, X. M. Genetic diversity of *Borrelia burgdorferi* Sensu Lato isolates from northeastern China. *Vector Borne Zoonotic Dis* **11**, 877–882 (2011).

33 Sun, Y., Liu, G., Yang, L., Xu, R. & Cao, W. *Babesia* microti-like rodent parasites isolated from *Ixodes persulcatus* (Acari: Ixodidae) in Heilongjiang Province, China. *Vet Parasitol* **156**, 333–339 (2008).

34 Du, Y. et al. A survey of ticks in an endemic focus of Lyme disease in Heilongjiang Province. *Chin J Pest Control* **8**, 33–35 (1992).

35 Gong, Z. D. et al. Composition and distribution of ticks in Hengduan Mountains, western Yunnan. *Chin J Pest Control* **17**, 31–33 (2001).

36 Hao, Y. J., Song, S. P., Gao, S. P. & Li, X. S. Investigation on ticks and mice in a Army. *Chin J Pest Control* **18**, 672–674 (2002).

37 Tian, Q. Y. Species of ticks in Shanxi province. *Medical Communication* **8**, 27–28 (1980).

38 Wei, A. M. et al. *Ehrlichia* DNA in ticks found in some parts of northeast china. *J Prev Med Chin PLA* **22**, 430–433 (2004).

39 Pan, L., Yu, E. S., Chen, Z. G. & Chen, Y. K. Study on the host animal and transmission vector of Lyme disease in Fujian Province. *Strail Journal of Preventive Medicine* **2**, 1–2 (1996).

40 Jiang, H. et al. *Ixodes persulcatus* parasitic on human body was found in Dongtai City, Jiangsu Province. *Chin J Parasitol Parasit Dis* **8**, 26 (1990).

41 Cao, W. C., Zhang, P. H., Zhang, X. T., Dai, X. H. & Xu, R. M. Detection and sequence analysis of *Ehrlichia chaffeensis* DNA in ticks. *Acta Parasitol Med Entomol Sin* **6**, 61–66 (1999).

42 Gao, D. Q., Cao, W. C., Zhao, Q. M., Zhang, X. T. & Fang, L. Q. Detection of human *Granulocytic Ehrlichia* 16s rRNA gene in ticks from North China. *Acta Parasitol Med Entomol Sin* **7**, 103–108 (2000).

43 Sun Y, Xu RM, Zhang PH, Guo TY & Cao WC. Isolation of Lyme agent *Borrelia* Spp. from several tick species in China. *Acta Parasitol Med Entomol Sin* **9**, 114–119 (2002).

44 Fan, D. H. et al. A study on population structures of ticks and their infected status by Lyme disease spirochetea at the 7 ports along the east of Heilongjiang Province. *Port Health Control* **11**, 40–42 (2006).

45 Fu, W. M., Wen, Z. Q., Yang, J. & Ju, W. D. Animal hosts and vector investigation of Lyme disease from China-Russia border in Heilongjiang Province. *Port Health Control* **14**, 28–35 (2009).

46 Hu, M. X. The investigation report on ticks at Suifenhe Port. *Port Health Control* **19**, 44–45 (2014).

47 Huang, C. A. & Teng, K. F. Ticks in Kashi and its adjacent areas, Xinjiang. *Acta Entomologica Sinica* **23**, 93–95 (1980).

48 Ma, G. Y. Collection and records of *Ascaris lumbricoides* and ticks of giant panda in Wen County of Gansu Province. *Sichuan Journal of Zoology* **6**, 34 (1987).

49 Ma, Y., Li, H. L., Wei, Y. W. & Wang, X. *Species investigation and faunal analysis of small mammals in Qinghai Province in 2011*. (Beijing, The 3rd National Conference on Zoonoses, 2011).

50 Cao, Y. X. et al. *Borrelia Burgdorferi* isolated from *Irodes Rersuleatus* in Xingjiang. *Endemic Diseases Bulletin* **3**, 13–20 (1988).

51 Xie, X. C., Yu, X., Zhang, T. X. & Jiang, G. Y. A survey report on the natural foci of Russian spring summer encephalitis in the mountainous areas of Tianshan and Altay mountains in Xinjiang. *Endemic Diseases Bulletin* **6**, 109–114 (1991).

52 Ye, R. Y. et al. Acarine fauna and its medical importance in southern Xinjiang. *Endemic Diseases Bulletin* **10**, 30–34 (1995).

53 Wang, Z. et al. Investigation on ticks-borne spotted fever group rickettsiae in western Liaoning region. *Animal Husbandry & Veterinary Medicine* **47**, 143–144 (2015).

54 Tao, N., Chai, Q. & Li, C. P. *Ixodes ovatus* found in Huainan area in Anhui Province. *Chin J Schisto Control* **29**, 647 (2017).

55 Hu, L. M. et al. Investigation on several species of tick carrying Elec in Changbai mountain area. *Chin J Public Health* **20**, 96–97 (2004).

56 Sun, D. W. et al. Seasonal succession of ticks in small wild animals in Chengmai county of Hainan province. *Chin J Public Health* **28**, 78–80 (2012).

57 Wu, Y. M. et al. Investigation on natural epidemic foci of tick-borne spotted fever in some regions. *Chin J Public Health* **19**, 25–26 (2003).

58 Cheng, C. et al. Investigation of emerging tick-borne diseases at Heilongjiang ports. *Chinese Frontier Health Quarantine* **38**, 176–181 (2015).

59 Cui, W. F. Ecological investigation of ticks in the suburb of Suifenhe. *Chinese Frontier Health Quarantine* **12**, 326–327 (1989).

60 Den, H., Gao, Y. F., Cheng, X. L. & Gao, Y. F. Detection and sequence analysis on nucleic acid of *Rickettsia japonica* in ticks of Manzhouli Port. *Chinese Frontier Health Quarantine* **37**, 336–338 (2014).

61 Fu, W. M., He, H., Hu, M. X., Cui, Y. M. & Yang, L. W. *Babesia* Microti-like rodent parasites isolated from *Ixodes Persulcatus* (Acari: Ixodidae) at China-Russia ports of Heilongjiang Province. *Chinese Frontier Health Quarantine* **33**, 99–104 (2010).

62 Fu, Y. Q. et al. Investigation of ticks and carried pathogens on Heixiazi island. *Chinese Frontier Health Quarantine* **38**, 119–123 (2015).

63 Han, H., Wu, H. L., Hu, X. F., Song, Y. J. & Xu, B. L. Surveillance on tick-borne pathogens at Heilongjiang ports. *Chinese Frontier Health Quarantine* **39**, 413–416 (2016).

64 Hu, G. et al. Investigation on ticks at Zhangmu Port, 2014. *Chinese Frontier Health Quarantine* **39**, 275–277 (2016).

65 Sun, X. F., Ding, S. L., Hui, M. X., Guo, X. M. & Zhao, G. Study on tick-borne *Rickettsia dermacentroxenus* infection at Heilongjiang ports. *Chinese Frontier Health Quarantine* **30**, 154–156 (2007).

66 Yang, L. W. et al. Investigation of ticks at 11 frontier ports of Heilongjiang Province. *Chinese Frontier Health Quarantine* **29**, 295–297 (2006).

67 Yang, L. W. et al. Study of tick-borne pathogens at Heilongjiang ports. *Chinese Frontier Health Quarantine* **30**, 77–82 (2007).

68 Yang, L. W. et al. The preliminary study on tick-borne diseases at Heilongjiang ports. *Chinese Frontier Health Quarantine* **32**, 354–362 (2009).

69 Zhu, J. G. et al. Surveillance on ticks in the Argun Port and adjacent forest zones, 2015. *Chinese Frontier Health Quarantine* **39**, 336–338 (2016).

70 Liu, Y. J., Jiang, Y. T., Guo, C. S. & Guan, B. P. Certain epidemiological characteristics of natural foci of tick-borne encephalitis in Jilin Province. *Bulletin of the Academy of Military Medical Sciences* **24**, 109–120 (1979).

71 Huang, H. N. et al. Investigation on *Borrelia burgdorferi* infection in ticks and animal from a forest area of Jilin Province. *Chinese Journal of Zoonoses* **22**, 785–788 (2006).

72 Shi, S. G., Zhang, F. & Liu, Z. J. Molecular epidemiological studies on *Borrelia burgdorferii* in ticks collected from several provinces and autonomous regions of northwestern China. *Chinese Journal of Zoonoses* **27**, 461–463 (2011).

73 Zhan, L. et al. Investigation on the infection with *Anaplasma phagocytophilum* in ticks from the forest area in Jilin Province of China. *Chinese Journal of Zoonoses* **23**, 438–440 (2007).

74 Cai, Z. L. Report on natural epidemic foci of tick-borne spotted fever in Dongning County, Heilongjiang Province. *Chinese Journal of Zoonoses* **2**, 35–37 (1986).

75 Zhang, Q. E. et al. A strain of spirechete isolated from *Ixodes Persuleatus* in Inner Mongolia. *Chinese Journal of Zoonoses* **6**, 5–6 (1990).

76 Zhao, Q. M. et al. Detection of HGE-like pathogen in *Ixodes Persulcatus* collected from Heilongjiang. *Chinese Journal of Zoonoses* **17**, 29–31 (2001).

77 Guo, T. Y., Xu, R. M., Ye, J. C. & Shi, X. L. Study on tick communities of the rodent in Dongling Mountain in Beijing. *Chin J Vector Biol & Control* **12**, 44–46 (2001).

78 Hao, Q. et al. Investigation and genotyping of Lyme bacteria in Jilin Province. *Chin J Vector Bio & Control* **18**, 303–305 (2007).

79 Hu, M. X. et al. Detection of rodent infected by new tick pathogens in Suifenhe and Dongning ports. *Chin J Vector Bio & Control* **20**, 573–576 (2009).

80 Lan, Y. Q. et al. Surveillance of tick-born infections during 2012–2013 in Lishui, Zhejiang. *Chin J Vector Biol & Control* **26**, 512–515 (2015).

81 Li, J. M. et al. Investigation on ticks and mice in forest areas of Beijing, China. *Chin J Vector Biol & Control* **13**, 165–168 (2002).

82 Li, Y., Zuo, S. Y., Tang, K. & Pu, G. Q. Investigation on the infection of ticks with *Anaplasma Phagocytophilum* in the northeast China forest region from 2010 to 2011. *Chin J Vector Biol & Control* **23**, 111–113 (2012).

83 Li, Y. X. et al. *Borrelia burgdorferi* isolated from *Ixodes sinensis* in the border areas between Guangxi and Guizhou in China. *Chin J Vector Biol & Control* **21**, 238–240 (2010).

84 Li, Z. J., Liu, Y. R., Liu, L. P., Dong, M. J. & Xiang, H. Y. A survey of tick in Yichang, Hubei with the delimitation of zoogeographical regions. *Chin J Vector Biol & Control* **13**, 200–201 (2002).

85 Ling, F. et al. Surveillance and study of tick-borne diseases in Zhejiang Province, China. *Chin J Vector Biol & Control* **24**, 19–23 (2013).

86 Liu, M. Z., Chen, W. H., Gu, W. Z. & Deng, F. C. Investigation on rats and insects on their body surface in Shenzhen. *Chin J Vector Biol & Control* **13**, 13–15 (2002).

87 Luo, J., Li, C. & Zheng, Y. The investigation of sucking louse and tick in Sanjiangyuan area. *Chin J Vector Biol & Control* **20**, 182 (2009).

88 Ma, Y. H. et al. Investigation of growth and decline of ticks parasitized on dogs in Guangzhou area. *Chin J Vector Biol & Control* **12**, 31–32 (2001).

89 Mei-Lang, J. C., Jia-Yong, S. D., Qi-Mei, Z. R. & Ma, L. M. A survey report on ticks and fleas in Qamdo area, Tibet. *Chin J Vector Biol & Control* **7**, 214–216 (1996).

90 Pan, L. et al. Investigation of pathogens and distribution of tick and *Tabanus* in the epidemic area of Lyme disease in Fujian Province. *Chin J Vector Biol & Control*, 58 (1993).

91 Shen, X. R. & Shen, D. R. Studies on the ticks and it's transmission diseases in Guizhou Province. *Chin J Vector Biol & Control* **8**, 287–288 (1997).

92 Sun, D. W., Zhao, W., Ceng, L. H. & Li, S. G. Investigation of species of Ixodidae and a new record species of Ixodidae in Hainan Province, China. *Chin J Vector Biol & Control* **24**, 442–443 (2013).

93 Sun, J. M. et al. Molecular epidemiological investigation of *Bartonella* infection in ticks in Zhejiang Province. *Chin J Vector Biol & Control* **21**, 232–234 (2010).

94 Tian, J. H. et al. One newly recorded genus and four newly recorded species of Ixodidae in Hubei Province, China. *Chin J Vector Biol & Control* **24**, 155–156 (2013).

95 Wei, Y. W., Li, C., Zheng, Y., Chen, H. J. & Wang, X. Investigation of fleas, tick and louse at the Sanjiangyuan Tanggula district of Qinghai Province. *Chin J Vector Biol & Control* **20**, 67–69 (2009).

96 Wu, W., Xia, D. F. & Huang, H. Q. Investigation on ticks and the pathogen of Lyme disease in Ningbo Port. *Chin J Vector Biol & Control* **23**, 109–111 (2012).

97 Xu, H. B. et al. Two newly recorded species of Ixodidae in Jiangxi Province, China. *Chin J Vector Biol & Control* **27**, 496–497 (2016).

98 Xu, Z. et al. Comprehensive surveillance of tick-borne diseases in Jiande, Zhejiang Province, China. *Chin J Vector Biol & Control* **25**, 350–353 (2014).

99 Yang, J. et al. Investigation on emerging tick-borne pathogens and co-infection in ticks in lava area of Xunke County, Heilongjiang Province. *Chin J Vector Biol & Control*, **27**, 341–344 (2016).

100 Yin, X. P. et al. Tick fauna and monitoring of tick host animals at Alataw Pass. *Chin J Vector Biol & Control* **21**, 375–377 (2010).

101 Yu, X., Re Zi-wan, A. B. L., Dai, X. & Zhang, Y. J. The relationship between ticks and diseases in Tarim basin and neibouring districts of Xinjiang. *Chin J Vector Biol & Control* **17**, 501–503 (2006).

102 Yu, X. H. et al. Investigation and analysis of ticks on body surfaces of host animals in Wenzhou City, Zhejiang Province, China. *Chin J Vector Biol & Control* **23**, 90–91 (2012).

103 Zhai, S. Y. & Zhao, Z. Q. Catalogue of main vectors in Shijiazhuang, China. *Chin J Vector Biol & Control* **24**, 446–450 (2013).

104 Zhang, D. C. et al. Insolation of *Borrelia burgdorferi* from ticks and mice in Liaoning. *Chin J Vector Bio & Control* **3**, 19 (1992).

105 Zhao, J. W., Wang, H. Y. & Wang, Y. Regional distribution profiles of tick-borne pathogens in China. *Chin J Vector Biol & Control* **23**, 75–78 (2012).

106 Zhao, Q. et al. Investigation of species, temporal and spatial distribution of ticks in Henan Province, China. *Chin J Vector Biol & Control* **26**, 75–77 (2015).

107 Zheng, S. G., Ye, X. D., Zheng, H. O., Huang, L. L. & Wang, S. B. Survey of rodents and their ectoparasitic ticks in Jinhua, China. *Chin J Vector Biol & Control* **25**, 183 (2014).

108 Wang, H. W. et al. Detection and genotyping of *Borrelia burgdorferi* sensu lato in ticks from some areas of China. *Journal of Pathogen Biology* **1**, 81–85 (2006).

109 Lei, M. T., Cai, J. Z., Li, C. H., Wang, F. & Sun, J. Overview of ectoparasite infection in yaks in China. *Chinese Journal of Veterinary Medicine* **52**, 68–70 (2016).

110 Li, M. et al. The Molecular detection of *Theileria orientalisi* in *Haemaphysalis longicornis* and *Ixodes persulcatus* in Yanbian area. *Chinese Journal of Veterinary Medicine* **52**, 90–92 (2016).

111 Liu, J. R., Mi, L., Wang, P. F., Zhang, Y. Y. & Bo, X. W. Faunal distribution and checklist of ticks in the Junggar basin. *Chinese Journal of Animal Infectious Diseases* **21**, 60–65 (2013).

112 Jiang, L. P. et al. Leptospira DNA of Lyme disease was first detected in tick in Zhejiang Province. *Chinese Journal of Health Laboratory Technology* **16**, 30–31 (2006).

113 Wu, Q. et al. Molecular detection of *Rickettsia* DNA in ticks around northeast region of China. *Chin Prev Med* **13**, 892–894 (2012).

114 Huang, W. L. et al. Investigation of the Russian spring summer encephalitis virus in Yunnan province. *Chinese Journal of Preventive Veterinary Medicine* **23**, 72–74 (2001).

115 Gao, Y. et al. Genotyping of *Borrelia burgdorferi* sensu lato isolated from Northeastern Forest Areas of China. *Chin J Microbiol Immunol* **26**, 404–408 (2006).

116 Chu, C. Y. et al. Investigation on *Borrelia buigdorferi* sensu lato in ticks and rodents collected in Da Xing An Mountain Forest areas of China. *Chin J Epidemiol* **27**, 681–684 (2006).

117 Huang, H. N. et al. Study on the coinfection status of *Borrelia burgdorferi* sensu lato and spotted fever group rickettsiae in ticks from Hunchun, Jilin province. *Chin J Epidemiol* **27**, 379–383 (2006).

118 Li, G. H. et al. Molecular epidemiology regarding *Anaplasma phagocytophilum* in *Dermacentor silvarum* in Ningwu County, Shanxi Province. *Chin J Epidemiol* **33**, 642–643 (2012).

119 Tang, K. et al. Dynamic investigation on the co-infection status of two pathogens in ticks from tourist point in Heilongjiang Province. *Chin J Epidemiol* **33**, 513–516 (2012).

120 Zhao, Q. M. et al. Study on the coinfection of three tick-borne infectious diseases in China using polymerase chain reaction method. *Chin J Epidemiol* **26**, 12–16 (2005).

121 Fan, D. H. et al. The situation of mice and ticks infected by *Babesia microtic*. *Chin J Hyg Insect & Equip* **18**, 54–56 (2012).

122 Han, H. et al. Infection status of *Borrelia burgdorferi* sensu lato and *Anaplasma phagocytophilum* in ticks from the region of Jingpo Lake Mudanjiang. *Chin J Hyg Insect & Equip* **21**, 154–157 (2015).

123 Wang, F. et al. Investigation on ticks in Dunhua forest area of Jilin Province. *Chin J Hyg Insect & Equip* **11**, 321–322 (2005).

124 Wang, W. et al. Investigation on ticks and the pathogens they could carry in Tianjin. *Chin J Hyg Insect & Equip* **19**, 220–222 (2013).

125 Yang, J., Xu, W. X., Bai, Y. S. & Liu, G. P. Surveillance and analysis of ticks in frontiers Boyaerkewo Russia and Xunke China. *Chin J Hyg Insect & Equip* **19**, 329–331 (2013).

126 Zhang, F. S., Liu, X. P., Gao, Y. & Ren, X. L. Investigation on the distribution of tick population in Benxi City. *Chin J Hyg Insect & Equip* **21**, 515–516 (2015).

127 Zhang, Z. Q., Hu, X. F., Hu, L. M., We, i. A. M. & Wu, Y. M. Investigation on ticks infected by *Ehrlichia* in parts of northeast region of China. *Shenyang Army Medicine* **18**, 35–37 (2005).

128 Zhang, Z. Q. et al. Detection and sequence analysis of *Ehrlichia chaffeensis* DNA from ticks in Northeast China. *Shenyang Army Medica* **18**, 75–76 (2005).

129 Liao, Q. P. & Wang, S. W. Survey of species of tick in Ovisn in Kashgar of Xinjiang. *Journal of Tarim University* **17**, 42–43 (2005).

130 Liang, F. X. Comprehensive prevention and treatment of external parasitic ticks in Red Deer of Tarim. *Special Economic Animal and Plant*, 16–17 (2009).

131 Cai, Z. L. et al. Epidemiological investigation of epidemic focus of tick-borne encephalitis in parts of three provinces in northeast China, China. *Journal of Microbiology* **16**, 19–22 (1996).

132 Zhang, Z. Q., Wei, A. M., Wu, Y. M., Hu, L. M. & Li, H. B. *Ehrlichia canis* DNA found in ticks of northeastern China. *Journal of Microbiology* **25**, 63–65 (2005).

133 Liao, G. P., Guo, C. H., Pan, X. Y., Yang, J. Z. & Zhang, F. S. Investigation of mites and ticks in livestock and poultry. *Journal of Agricultural Science* **88**, 295–296 (1992).

134 Liu, G. P., Yang, G. P., Ren, Q. M., Wang, F. & Wu, C. G. Study of species composition on the ectoparasitical acarinids of the rodent in frontiers of northeast China. *Acta Arachnologica Sonica* **17**, 106–110 (2008).

135 Liu, Y. R. & Yang, Z. Q. The tick fauna in Hubei Province. *Acta Arachnologica Sinica* **10**, 18–21 (2001).

136 Liu, Y. H., Fang, K., Wang, H. W., Han, Y. H. & Zhou, H. Y. A case of tick bite by *Ixodes persulcatus*. *J Clin Dermatol* **31**, 720 (2002).

137 Gao, D. Q., Cao, W. C., Zhang, X. T., Zhao, Q. M. & Zhu, J. H. Investigation of human ehrlichiosis foci in the Da Xing An Mountains of Inner Mongolia. *Infect Dis Info* **14**, 168–170 (2001).

138 Gao, Y. M. et al. Molecular epidemiological investigation on human monocytic ehelichiosis in the forest area of northwestern Fujian, China. *Infect Dis Info* **14**, 126–127 (2001).

139 Zhang, P. H. et al. Molecular epidemiological investigation of tick-borne spotted fever in some parts of China. *Infect Dis Info* **14**, 27–28 (2001).

140 Wang, X. E., Li, S. S., Yang, L. S. & Shen, X. H. A list of ticks form Gansu, China. *J Lanzhou University* **16**, 87–92 (1980).

141 Liu, Z. & Tian, J. H. A new record species of tick from Hubei province. *Journal of Huazhong Normal University (Nat. Sci.)* **48**, 735–736 (2014).

142 Liu, Y. T. et al. Investigation of ticks in scenic spots and surrounding areas in Heilongjiang Province. *Modern Animal Husbandry Science & Technology* **44**, 25–26 (2016).

143 Liu, M. S., Huang, K. J., Zhao, Z. F., Zhang, L. Z. & Guo, P. R. Investigating the distribution of ticks among nine provinces in china. *Journal of Changzhi Medical College* **19**, 249–250 (2005).

144 Wang Xin-e, et al. A report of ticks of the three prefectures on Hexi Corrider in Gansu Province. *Journal of Lanzhou University* **22**, 97–101 (1986).

145 Pang Dao-mao, Ai Cheng-xu & Guo-shi, C. Fauna and vertical distribution of Ticks in northern Xinjiang. *Sichuan Journal of Zoology* **5**, 15–18 (1985).

146 Wei-xian, L. The ecogeographical distribution of ticks in Liaoning Province. *Acta Entomologica Sinica* **30**, 180–185 (1987).

147 Liu Lian-kun & Jian-jun, G. Species of poultry ticks and its dominant species in Guizhou Province. *Journal of Southwest China Normal University(Natural Science Edition)* **36**, 98–101 (2011).

148 Takada, N. et al. Lyme disease spirochetes in ticks from northeastern China. *J. Parasitol.* **84**, 499–504 (1998).

149 Yu, P.-F., et al. Molecular epidemiological surveillance to assess emergence andre-emergence of tick-borne infections in tick samples from China evaluated by nested PCRs. *ACTA Trop* **158**, 181–188 (2016).

150 Liu Guo-ping, et al. Distribution and medical importance of ticks in three provinces of northeast China. *Chinese Journal of Hygienic Insecticides & Equipments* **63**, 39–42 (2008).

151 Hua man-tang, et al. Investigation on common ticks carrying *Borrelia burgdorferi* in Tierje Ketilin pastoral area of Haba River. *Chinese Journal of Zoonoses* **16**, 105–110 (2000).

152 Lei, Z. *Tick-borne Pathogent Investigation in Inner Mongolia Forest Area. Master Dissertation*. Chinese People's Liberation Army Academy of Military Medical Sciences, (2017).

153 Shang-shu, G. *Cloning and sequence analysis of full-length genome of Nairobi sheep disease virus in ticks from northeastern China*. Master Dissertation. Jilin Agricultural University, (2015).

154 Hua Man-tang, J. Z.-q., Fan Yin-cang, et al. Investigations on ticks in afforested and livestock breeding area of Haba River TieReKeTi. *Medical Animal Control* **15**, 563–565 (1999).

155 Zhang, L. Y. *Preliminary investigation of phagocytic anaplasmosis infection in animals in Hubei province*. (Beijing, The 3rd national conference on zoonoses. 2011).

156 Tian, H. et al. Severe fever with thrombocytopenia syndrome virus in humans, domesticated animals, ticks, and mosquitoes, Shaanxi Province, China. *Am J Trop Med Hyg* **96**, 1346–1349 (2017).

157 Li, W., Liu, L., Jiang, X., Guo, X., Garnier, M., Raoult, D., Parola, P. Molecular identification of spotted fever group rickettsiae in ticks collected in central China. *Clin Microbiol Infect* **15**, 279–280 (2008).

158 Luo, L. M. et al. *Haemaphysalis longicornis* ticks as reservoir and vector of severe fever with thrombocytopenia syndrome virus in China. *Emerg Infect Dis* **21**, 1770–1776 (2015).

159 Wang, D., Wang, Y., Yang, G., Liu, H. & Xin, Z. Ticks and tick-borne novel bunyavirus collected from the natural environment and domestic animals in Jinan City, East China. *Exp Appl Acarol* **68**, 213–221 (2016).

160 Chen, Z., Liu, Q., Jiao, F. C., Xu, B. L. & Zhou, X. N. Detection of piroplasms infection in sheep, dogs and hedgehogs in Central China. *Infect Dis Poverty* **3**, 18 (2014).

161 Zhou, Y., Zhang, H., Cao, J., Gong, H. & Zhou, J. Epidemiology of toxoplasmosis: role of the tick *Haemaphysalis longicornis*. *Infect Dis Poverty* **5**, 14 (2016).

162 Cao, W. C. et al. Identification of *Ehrlichia chaffeensis* by nested PCR in ticks from Southern China. *J Clin Microbiol* **38**, 2778–2780 (2000).

163 Zhang, J. Z. et al. Genetic classification of "*Rickettsia heilongjiangii*" and "*Rickettsia hulinii*," two Chinese spotted fever group rickettsiae. *J Clin Microbiol* **38**, 3498–3501 (2000).

164 Abdallah, M. O. et al. Identification of 12 piroplasms infecting ten tick species in China using reverse line blot hybridization. *J Parasitol* **103**, 221–227 (2017).

165 Hoogstraal, H., el-Kammah, K. M., Santana, F. J. & Van Peenen, P. F. Studies on southeast Asian Haemaphysalis ticks (Ixodoidea: Ixodidae). H. (Kaiseriana) lagrangei Larrousse: identity, distribution, and hosts. *J Parasitol* **59**, 1118–1129 (1973).

166 Chen, Z. et al. Tick-borne pathogens and associated co-infections in ticks collected from domestic animals in central China. *Parasit Vectors* **7**, 237 (2014).

167 Li, Y. et al. First report of *Theileria* and *Anaplasma* in the Mongolian gazelle, Procapra gutturosa. *Parasit Vectors* **7**, 614 (2014).

168 Meng, H. et al. Abundance and seasonal activity of *Haemaphysalis concinna* (Acari: Ixodidae) at the border between China and Russia in Northern Inner Mongolia, China. *Parasit Vectors* **9**, 1 (2016).

169 Wang, Y. Z. et al. A broad-range survey of ticks from livestock in Northern Xinjiang: changes in tick distribution and the isolation of *Borrelia burgdorferi* sensu stricto. *Parasit Vectors* **8**, 449 (2015).

170 Yang, J. et al. Molecular survey and characterization of a novel *Anaplasma* species closely related to *Anaplasma capra* in ticks, northwestern China. *Parasit Vectors* **9**, 603 (2016).

171 Yang, J. et al. Novel spotted fever group rickettsiae in *Haemaphysalis qinghaiensis* ticks from Gansu, Northwest China. *Parasit Vectors* **9**, 146 (2016).

172 Meng, K. et al. First detection of severe fever with thrombocytopenia syndrome virus in the tick species *Haemaphysalis concinna i*n Shandong Province, China. *Parasitol Res* **114**, 4703–4707 (2015).

173 Omar Abdallah, M. et al. Identification of piroplasm infection in questing ticks by RLB: a broad range extension of tick-borne piroplasm in China? *Parasitol Res* **115**, 2035–2044 (2016).

174 Jiao, Y. et al. Experimental and natural infections of goats with severe fever with thrombocytopenia syndrome virus: Evidence for ticks as viral vector. *PLoS Negl Trop Dis* **9**, e0004092 (2015).

175 Chao, L. L., Hsieh, C. K. & Shih, C. M. First report of *Amblyomma helvolum* (Acari: Ixodidae) from the Taiwan stink snake, Elaphe carinata (Reptilia: Colubridae), collected in southern Taiwan. *Ticks Tick Borne Dis* **4**, 246–250 (2013).

176 Dong, X., Chen, X. P., Liu, N., Dumler, S. J. & Zhang, Y. Z. Co-circulation of multiple species of Rickettsiales bacteria in one single species of hard ticks in Shenyang, China. *Ticks Tick Borne Dis* **5**, 727–733 (2014).

177 Luo, L. et al. Detection of a novel *Ehrlichia* species in *Haemaphysalis longicornis* tick from China. *Vector Borne Zoonotic Dis* **16**, 363–367 (2016).

178 Xia, L. Z., Liu, C. W., Wang, Z. X. & Du, J. S. The first records of ticks in Anhui province. *Journal of Anhui Medical College* **16**, 32–36 (1981).

179 Ma, Y. H. et al. Investigation and control of ectoparasitic ticks on Dogs in South China. *Chinese Journal of Pest Control* **17**, 590–592 (2001).

180 Zeng, X. X. et al. A survey report on the pathogenic species of bovine hemosporidiosis and tick transmission in Henan Province. *Henan agriculture and forestry science and technology* **11**, 31–34 (1982).

181 Fan, G. Y., Liu, J. W., Wang, S. G., Zhong, H. & Chen, J. J. Diagnosis and curat of tick paralysis and *Babesia gibsoni* infection of the tibetan mastiff canine induced by tick invasion. *Journal of Henan Agricultural Sciences* **40**, 167–170 (2011).

182 Zhang, Q. Z., Luo, H. L., Li, X. W. & Dong, Z. M. Survey on ticks and pathogens in the serum of its host in Xinyang city, Henan province. *Journal of Henan Normal University (Natural Science Edition)* **44**, 126–130 (2016).

183 Pan, Y. B. Isolation and morphological observation of tyrannosae from Gannan area of Gansu Province. *Gansu Animal and Veterinary Sciences* 23–24 (2003).

184 Sun, C. Q. *Development of the PCR for detection of piroplasms infective to cattle and sheep in Haemaphysalis qinghaiensis and its application*, Master Dissertation, Gansu Agricultural University, (2005).

185 Pu, Y. et al. Detection and analysis on important ticks and tick-borne pathogen at Jilin Port areas, China. *Acta Parasitol Med Entomol Sin.* **16**, 228–232 (2009).

186 Sun, Y., Xu, R. M. & Wei, C. C. Haemaphysalis tick (Ixodoidae, Ixodidae) in China: systematic and key to subgenera. *Acta Parasitol Med Entomol Sin* **18**, 251–258 (2011).

187 Sun Y, Xu RM & Wei CC. Haemaphysalis tick (Ixodoidae, Ixodidae) in China: Subgenus Ornithophysalis, with a new record species. *Acta Parasitol Med Entomol Sin* **19**, 50–56 (2012).

188 Tang, L., Jiang, S. & Tang, H. Community analysis of *haemaphysalis longicornis* from wolf and sheep. *Hunan Journal of Animal Science & Veterinary Medicine* **38**, 36–39 (2016).

189 Fan, G. Y., Liu, J. W. & Zhong, H. Diagnosis and Prevention of Sheep Theileriasis. *Hubei Agricultural Sciences* **51**, 2783–2785 (2012).

190 Sun, B. J., Xue, X. N. & Hou, W. Background investigation of tick and acarid at Qingdao international airport. *Port Health Control* **14**, 39–41 (2009).

191 Tian, Q. Y. Haemaphysalis Concinna, New Record of Haemaphysalis in Shanxi. *Journal of shanxi medical college* **20**, 128 (1989).

192 Wang, P. X., Wang, X. E. & Xu, X. Q. Ticks on the birds and mammals from Eastern Gansu, China. *Sichuan J. Zoology* **6**, 40–41 (1987).

193 Liu, N., Dong, X. & Li, X. Detection of novel tick-borne Bunyavirus in Shenyang. *Disease Surveillance* **29**, 63–66 (2014).

194 Luo, D. et al. The first detection of *Coxiella burnetii* DNA from *Hyalomma asiaticum* at Alataw pass, China-Kazakhstan border area. *Disease surveillance* **31**, 814–816 (2016).

195 Liu, J. R., Annas, D., Serikbul, H. & Zhang, Y. Y. Faunal distribution and community analysis of ticks in Altay region. *Xinjiang Agricultural Sciences* **50**, 1165–1170 (2013).

196 Qi, F. X., Yang, J. & Wang, D. H. Investigation on parasitic ticks of sheep in eastern Qinghai province. *Chinese Qinghai Journal of Animal and Veterinary Sciences* 16 (1991).

197 Wang, K. S. et al. The investigation and analysis of the species composition of geographical distribution of parasitic ticks in the Gurbantonggut Desert. *Journal of shihezi University (Natural Science)* **29**, 575–578 (2011).

198 Zhang, L. et al. Morphology and 16S rDNA sequence analysis of ixodid ticks collected from Boertonggu Pasture in Shihezi-Shaw an Region, Xinjiang. *Journal of Shihezi University (Natural Science)* **32**, 296–301 (2014).

199 Zhang, Y. Y., Adili, K., Damu, B., Wang, K. S. & Liu, J. R. Species investigation and community analysis of ticks in Tacheng area. *Journal of Shihezi University (Natural Science)* **31**, 457–462 (2013).

200 Liu, L. Z., Li, G. P. & Yu, S. R. Study on the infection of *Haemaphysalis Belliculata* against *Rickettsia* Q Fever. *Acta Academiae Medicinae Militaris Tertiae*, 17–21 (1980).

201 Ye, R. Y., Yu, X., Cao, H. L. & Chen, X. R. The fauna of ticks and medical mites in the Ertix River Valley and its Neighboring Mountainous Areas in Xinjiang, China. *Endemic Diseases Bulletin* **5**, 27–31 (1990).

202 Luo, J. X. et al. Collection and identification of *Piroplasma* infected to cattle and sheep in China. *Chin J Parasitol Parasit Dis* **24**, 48–53 (2006).

203 Li, A. J., Ma, Z. P., Zhang, J. J., Xue, H. J. & Wang, H. L. Species survey of ticks in Pingdingshan City. *Chinese Journal of Public Health Management* **29**, 381–382 (2013).

204 Gu, J. Q. et al. Background investigation of ticks and midges in Changshu Port. *Chinese Frontier Health Quarantine* **30**, 230–231 (2007).

205 Xue, X. N., Hou, W. & Sun, B. J. Investigation on medical vectors at Qingdao International Airport. *Chinese Frontier Health Quarantine* **32**, 22–26 (2009).

206 Chen, Z. *Study on piriformis infection in different hosts in Xinyang, Henan Province*, Master Dissertation, Chinese Center for Disease Control and Prevention, (2014).

207 Li, Y. et al. Prevalence and coexistence of *Anaplasma phagocytophilum* and *Borrelia burgdorferi* sensu lato in ticks in Gansu, Hunan and Guangdong provinces, China. *Chinese Journal of Zoonoses* **29**, 117–121 (2013).

208 Yu, Q. et al. Genetic identification of *A. phagocytophilum* from *H. longicornis* in Laizhou Bay of Shandong Province, China. *Chinese Journal of Zoonoses* **28**, 15–18 (2012).

209 Gao, Y. M. et al. Detection of *Ehrlichia chaffeensis* in ticks and rodents using semi-nested PCR. *Chinese Journal of Zoonoses* **16**, 25–28 (2000).

210 An, J. Y., Zhou, G. P., Yao, G. L., Ai, C. X. & Li, Q. M. Morphological observation of *Haemaphysalis congicornis* and *Borrelia Burgdorferi* in the tick' s body in Xishan Mountain in Beijing. *Chin J Vector Bio & Control* **13**, 282–284 (2002).

211 Cao, X. M. et al. Detection of pathogen infections in parasitic ticks of wild rodents at Erenhot Port. *Chin J Vector Biol & Control* **25**, 127–130 (2014).

212 Feng, S. et al. Molecular epidemiological study of tick-borne spotted fever group rickettsiae in western mountain area of Hebei province, China. *Chin J Vector Biol & Control* **24**, 308–312 (2013).

213 Guo, T. Y. et al. Surveillance of ectozoa on the body of rodent and hedgepig in Beijing ports. *Chin J Vector Bio & Control* **20**, 27–29 (2009).

214 Han, H., Yang, Y., Tan, K. W., Song, Y. J. & Xu, B. L. Analysis on risk of tick-borne pathogens at Zhangmu Port in Tibet. *Chin J Vector Biol & Control* **27**, 539–541 (2016).

215 Li, Y. L. et al. Lyme disease spirochetes isolated from *Haemaphysalis bispinosa* in the forest area of East Sichuan. *Chin J Vector Biol & Control* **2**, 386–387 (1991).

216 Liu, S. L. et al. An endemic investigation of Lyme disease in Yimeng Mountains, Shandong Province. *Chin J Vector Bio & Control* **11**, 194–196 (2000).

217 Ma, Y. H. et al. Collection records of Ixodidae in south China. *Chin J Vector Biol & Control* **10**, 85 (1999).

218 Wang, L. J. et al. First isolation and identification of Lyme disease spirochete in Shandong province. *Chin J Vector Bio & Control* **8**, 127–128 (1997).

219 Wang, Y. H. et al. *Francisella tularensis* was detected in *Haemaphysalis longicornis* from Liaoning province *Chin J Vector Bio & Control* **27**, 529–532 (2016).

220 Weng, C. R. et al. Investigation on the distribution of ticks and surveillance of the density of *Haemaphysalis bispinosa* in Guizhou. *Chin J Vector Biol & Control* **14**, 376–377 (2003).

221 Wu, H. X. et al. First detection of *Bartonella henselae* infection in *Haemaphysalis longicornis*. *Chin J Vector Bio & Control* **26**, 16–18 (2015).

222 Xin, Z. et al. Status of tick distribution and tick-borne pathogens in Jinan city. *Chin J Vector Biol & Control* **26**, 179–181 (2015).

223 Ye, L., Ren, Y., Wang, Z. F., Wang, C. W. & Li, S. B. Surveillance and analysis of new Bunia virus infections in Zhoushan Island area during 2011–2012. *Chin J Vector Biol & Control* **24**, 429–431 (2013).

224 Yue, J. N. & Shi, Y. Epidemiological investigation of Lyme disease in parts of forest areas in Qinghai Province. *Chin J Vector Biol & Control* **20**, 358–359 (2009).

225 Zhang, H. L. et al. Spotted fever group rickettsiae DNA was detected in wild rodents and ticks in Dali, Yunnan Province, China. *Chin J Vector Bio & Control* **15**, 461–462 (2004).

226 Wang, L. Y. et al. Survey and genetic analysis of severe fever with thrombocytopenia syndrome virus from *Haemaphysalis longicornis*. *Journal of Pathogen Biology* **9**, 59−62 (2014).

227 Zhang, F. & Liu, Z. J. Molecular epidemiological studies on *Coxiella burnetii* from Northwestern China. *Journal of Pathogen Biology* **6**, 183−185 (2011).

228 Liu, A. H. et al. Isolation and identification of two *Piroplasma* species from Wenchuan County in Sichuan. *Veterinary Science in China* **36**, 972−975 (2006).

229 Sun, J. G., Li, K. J., Wei, H. & He, Z. W. Studies of *Haemaphysalis concinna* outbreaks in Cangxi County of Sichuan Province. *Veterinary Science in China* **36**, 719−723 (2006).

230 Dou, X. F., Fu, F. L., Wang, Y. P. & Ma, X. W. Investigation and control of blood protozoonoses in sheep. *Chinese Journal of Veterinary Science and Technology* **34**, 39−41 (2004).

231 Hui, Y. et al. Epidemiological investigation of Ovinetheileriasis in Zhangjiachuan county, Gansu province. *Chinese Journal of Veterinary Science and Technology* **30**, 16−17 (2000).

232 Luo, J. X. et al. Investigation and distribution characteristics of Ovinetheileriasis in Gansu Province. *Chinese Journal of Veterinary Science and Technology* **27**, 15−16 (1997).

233 Shu, Z. et al. Transmission test of ticks borne Ovinetheileriasis in Gansu Province. *Chinese Journal of Veterinary Science and Technology* **31**, 16−17 (2001).

234 Song, J. G., Li, W. K., Xue, Y. P., Wang, Z. D. & Ha, J. M. Tick survey of sheep blood protozoa endemic area in central Gansu province. *Chinese Journal of Veterinary Science and Technology* **29**, 26−27 (1999).

235 Zhang, S. F., Xu, Y. T., Jin, H. X., Song, J. C. & Li, W. X. Investigation on the floristic and ecological characteristics of ticks in some pastures in the lower Tumen River. *Chinese Journal of Veterinary Science and Technology* **28**, 18−19 (1998).

236 Li, Y. et al. Identification and phylogenetic analysis of spotted fever group rickettsiae isolated from Qinghai Province. *Chin J Vet Sci* **34**, 1956−1961 (2014).

237 Meng, Q. L. et al. Survey on tick-borne *Anaplasmataceae* in the south edge of Gurbantunggut Desert. *Chin J Vet Sci* **32**, 73−78 (2012).

238 Zhou, Y. X. et al. Molecular epidemiological of spotted fever group rickettsiae from ticks in Tangshan area. *Chin J Vet Sci* **31**, 1729−1732 (2011).

239 Dou, X. F., Gao, D. H. & Fu, P. Z. Investigation on tick infection of sheep in cold wet area of Zhangjiachuan County. *Chinese Journal of Veterinary Parasitology* **6**, 28−29 (1998).

240 Guo, S. Z. et al. Epidemiological investigation on tylerosis of sheep in northern China. *Chinese Journal of Veterinary Parasitology* **13**, 15−17 (2005).

241 Guo, S. Z. et al. Epidemiological investigation of sheep Theileriasis in Gannan Tibetan Autonomous Prefecture. *Chinese Journal of Veterinary Parasitology* **11**, 40−41 (2003).

242 Mu, Y. J. et al. Investigation and control of sheep Theileriasis in Gannan Tibetan Autonomous Prefecture. *Chinese Journal of Veterinary Parasitology* **16**, 25−28 (2008).

243 Wang, W. B. & Ma, D. L. Epidemiological investigation of sheep Theileriasis in Aksu, Xinjiang. *Chinese Journal of Veterinary Parasitology* **10**, 27−28 (2002).

244 Yin, H. et al. Experimental transmission of some bovine and ovine tick-borne haemoprotozoans in Gansu Province. *Chinese Journal of Veterinary Parasitology* **8**, 19−21 (2000).

245 Pan, Y. B. Isolation and morphological observation of *Theileria Hirci* in Gannan. *Chinese Journal of Veterinary Medicine* **39**, 24−25 (2003).

246 Wang, W. B., Liu, Z. B., Bao, X. J., Liu, W. C. & Ye, J. H. Epidemiological investigation of tick (ixodida) on police dogs in some areas of China. *Chinese Journal of Veterinary Medicine* **49**, 17−20 (2013).

247 Xian, S. W., Chen, D. L., Wen, X. C., Deng, H. & Zhou, Z. T. Controlling of *Haemaphysalis Concinna* Outbreak. *Chinese Journal of Veterinary Medicine* **38**, 31−32 (2002).

248 Xian, S. W., Chen, D. L., Wen, X. C., Luo, B. P. & Tan, Y. X. Investigation on *Haemaphysalis Concinna* Epidemic in Bazhong. *Chinese Journal of Veterinary Medicine* **38**, 24−25 (2002).

249 Chen, Q. Y. et al. Molecular epidemiological investigation of ticks and tickborne pathogens in domestic animals in Kazak Autonomous Prefecture of Ili, Xinjiang, China. *Chinese Journal of Animal Infectious Diseases* **24**, 72−77 (2016).

250 Song, L. H. et al. Bunyaviruses detected from ticks positive for nucleic acid. *China Tropical Medicine* **12**, 129−130 (2012).

251 Wang, G. R. Investigation and control report of Ovinetheileriasis in Lintan, Gansu. *Journal of Traditional Chinese Veterinary Medicine* **35**, 68 (2016).

252 Hao, Y. J. et al. A new type of spotted fever group rickettsia detected in the area of Changbai Mountain, Jilin Province. *Chin J Epidemiol* **24**, 62−64 (2003).

253 Zhang, L. et al. Isolation of *Borrelia burgdorferi* in Ixodes from four counties, in North Xinjiang. *Chin J Epidemiol* **35**, 262−265 (2014).

254 Cao, X. J. et al. Survey for the tick species parasitized in sheep and the tick-borne pathogeny in Erenhot. *Chin J Hyg Insect & Equip* **20**, 421−423 (2014).

255 He, X. Y., Li, Z. Q. & Zhang, J. M. Preliminary study on natural infection of *Francisella tularensis* in parasitic ticks from Shaanxi Province. *Chin J Hyg Insect & Equip* **20**, 87−89 (2014).

256 Liu, G. P., Ren, Q. M., Wang, F., Sun, W. & Han, X. N. Study on ecology and control counter measures of ticks in frontiers of Russia, Korea and northeast China. *Chin J Hyg Insect & Equip* **12**, 208−210 (2006).

257 Lv, X. H. et al. Investigation and analysis on species and distribution of ticks in Songjiang District of Shanghai. *Chin J Hyg Insect & Equip* **21**, 378−380 (2015).

258 Yuan, Z. L., Liu, J. Q., Wang, G. Z., Wang, Z. G. & Lv, M. J. Investigation on tick hosts and seasonal fluctuation in Dengfeng Shaolin temple scenic spot. *Chin J Hyg Insect & Equip* **19**, 332−333 (2013).

259 Zhang, J. J., Xing, D. M. & Liu, J. Q. Preliminary study on population density of ticks in Pingdingshan City in 2011−2012. *Chin J Hyg Insect & Equip* **19**, 229−231 (2013).

260 Chen, Z. G., Chen, M., Zhong, J. P. & Bi, Z. D. Using PCR/ RFLP to detect spotted fever group rickettsiae in ticks and rodents collected in Ninghua, Fujian Province. *Chin J Prev Med* **36**, 35−37 (2002).

261 Xing, Y. F. et al. Epidemiological investigation of predominance tick and the infectious status of severe fever thrombocytopenia syndrome virus in Penglai and Laizhou counties, Shandong Province. *Chin J Prev Med* **49**, 995−999 (2015).

262 Jiang, X. L. et al. Isolation, Identification and characterization of SFTS bunyavirus from ticks collected on the surface of domestic animals. *Chinese Journal of Virology* **28**, 252−257 (2012).

263 Chen, Z. G., Chen, M., Zhong, J. P., Bi, Z. D. & Zhang, P. H. Detection of spotted fever group rickettsiae (SFGR) by PCR/RFLP in ticks and rodents collected in Ninghua, Fujian. *Science of Travel Medicine* **6**, 30−32 (2002).

264 Lai, C. L. & Liao, G. H. Investigation and control of Lyme disease vector ticks in Guangxi. *Guangxi Journal of Preventive Medicine* **1**, 43−44 (1995).

265 Cao, Y., Guan, W. Y., Cai, Z. C. & Yu, T. Preliminary observation on wintering habits of *haemaphysalis longicornis* in Fangshan District, Beijing. *Heilongjiang Animal Science and Veterinary Medicine* **58**, 75−77 (2015).

266 Zhang, L. J., Zhang, J. S., Fu, X. P. & Luan, M. C. First identification of a *Rickettsia* closely related to *R. heilongjiangii* and *R. massilliae* in South China. *Infect Dis Info* **19**, 65−67 (2006).

267 Sun, C. X. et al. First discovery of *Haemaphysalis danensis* in Sunan, Gansu. *Journal of Lanzhou University (Natural Sciences)* **32**, 138 (1996).

268 Zhao, H. M., Su, R. Y., Wan, K. L. & Wu, N. Epidemiological investigation of Lyme disease in Xilingole District of Inner Mongolia. *Progress In Veterinary Medicine* **23**, 72−74 (2002).

269 Teng, G. F. & Cui, Y. Q. Descriptions of a new species of Haemaphsalis and the male of H. primitive Teng, 1982, from Yunnan (Acarina: Ixodidae). *Acta Zootaxonomica Sinica* **9**, 37−40 (1984).

270 Teng, K. F. Two new species of Haemaphysalis from China(Acarina: Ixodedae). *Acta Zootaxonomica Sinica* **5**, 144−149 (1980).

271 Zhang, Q. Z. et al. Investigation and analysis of tick distribution and tick vector in Yunan district. *Modern Preventive Medicine* **42**, 3578−3580 (2015).

272 Xing, D. R. Investigation on the occurrence of *Haemaphysalis bispinosa* in Jinzhou. *Liaoning Animal Husbandry and Veterinary Medicine* **9,** 15−17 (1980).

273 He, Y. Z. et al. Investigation on mites in tussah silkworm farms in Fengcheng, Liaoning and their protective measures. *Liaoning Agricultural Sciences* **54**, 50−51 (2013).

274 Xu, X. H. et al. Study on the classification of ticks and etiology of novel bunyavirus in ticks, Laizhou City. *Prev Med Trib* **21**, 1−3 (2015).

275 Li, J. M. et al. Investigation on ticks and mice in forest areas of Beijing, China. *Chinese Journal of Vector Biology and Control* **13**, 165–168 (2002).

276 Liu Ming-she, H. K.-j., Zhao Zhong-fu, et al. Investigating the distribution of ticks among nine provinces in China. *Journal of Changzhi Medical College* **19**, 249–250 (2005).

277 Lin, Z. *Study on geographical distribution and detection pathogeny of ticks, north of Xinjiang.* Master Dissertation. Shihezi University, (2014).

278 Xu Hong-bin, H. H., Xu Zhong-ji, et al. Six New Records of Ixodidae from Jiangxi Province. *Journal of Central China Normal University (Natural Science)* **51**, 804–808 (2017).

279 Yang, L. *Survey on ticks and detection of new bunyavirus in theendemic areas of fever, thrombocytopenia and leukopeniasyndrome in Henan province*. Master Dissertation. Zhengzhou University, (2012).

280 Zhu, C. J. & Zheng, X. C. A new record of *Aponomma lucasi Warburton* in Sichuan. *Acta Universitats Scientiae Medicinae Chongqing* **19**, 19−20 (1994).

281 Teng, K. F. A new species of Amblyomma from Hainan Island, China. (Acarina: Ixodidae). *Acta Zootaxonomica Sinica* **6**, 399−401 (1981).

282 Tsui, P. Y. et al. Molecular detection and characterization of spotted fever group rickettsiae in Taiwan. *Am J Trop Med Hyg* **77**, 883−890 (2007).

283 Zhang, J. et al. First report of *Rickettsia felis* in China. *BMC Infectious Diseases* **14**, 682 (2014).

284 Li, Y. et al. *Anaplasma* infection of Bactrian camels (*Camelus bactrianus*) and ticks in Xinjiang, China. *Parasit Vectors* **8**, 313 (2015).

285 Xia, H. et al. Metagenomic profile of the viral communities in *Rhipicephalus* spp. ticks from Yunnan, China. *PloS one* **10**, e0121609 (2015).

286 Wu, J. Y., Chen, S. P., Yan, F. F. & Wang, Y. Z. Morphology and molecular biology identification of *Rhipicephalus turanicus*, Turtle in Kuche County, Xinjiang. *Jiangsu Agricultural Science* **45**, 149−151 (2017).

287 Xiu, F. X. & Sun, N. Investigation on the infection of ticks on dogs in Liaoning area. *Shandong Journal of Animal Science and Veterinary Medicine* **35**, 56 (2014).

288 Wu, Y. H., Liu, L., Wang, Z. & Bayinchahn. Investigation of vector ticks and Piroplasmosis infection in scattered feeding cattle farm in southern Xinjiang. *Xinjiang Agricultural Sciences* **48**, 1918−1922 (2011).

289 Jiang, D. X. et al. Investigation on the species and pathogens of parasitic ticks in cattle in Guangdong Province. *Animal Husbandry & Veterinary Medicine* **47**, 95−98 (2015).

290 Hou, K. Q. Comprehensive prevention and treatment report of *Rhipicephalus Microplus* causing explosive anemia in cattles. *Animal husbandry and veterinary science* **1**, 64−66 (1982).

291 Liu, Z. L., Ma, L. H., Gao, X. S., Chen, X. J. & Wang, S. Y. Studies on the seasonal activity and some biological characters of *Rhipicephalus Haemaphysaloides Haemaphysaloides Spino*. *Chinese Journal of Animal and Veterinary Sciences*, **36**, 155−160 (1988).

292 Zhang, Y. J. et al. Classification and diversity of tick community in Tarim Basin. *Chin J Parasitol Parasit Dis* **24**, 404−409 (2006).

293 Ya, H. X., Shen, S., Su, Z. Y., Deng, F. & Zhang, Y. Z. Tick species and genetic variants analysis of tick gene in Hengduan Mountains, west Yunnan Province, China. *Chinese Journal of Zoonoses* **32**, 865−870 (2016).

294 Ya, H. X., Zhang, Y. Z. & Wang, J. L. Molecular Identification of tick species and sequence analysis of *Ehrilichia* in Lincang area of Yunnan province, China. *Chinese Journal of Zoonoses* **32**, 793−797 (2016).

295 Zhang, L. J., Zhang, J. S., Fu, X. P. & Luan, M. C. The new tick-borne spotted fever group rickettsia exists in ticks from Lianping County of Guangdong province. *Chinese Journal of Zoonoses* **22**, 697−700 (2006).

296 Zhao, Q. L. et al. 16rs RNA sequence analysis of *Anaplasma* and *Ehrlichia* in rodents from parts area of Xinjiang. *Chinese Journal of Zoonoses* **29**, 743−747 (2013).

297 Pan, H. et al. *Ehrlichia cains* DNA found in ticks in the south of China. *Chinese Journal of Zoonoses* **15**, 3−6 (1999).

298 Liu, Y. H. & Zhao, L. Analysis of bm86 gene and its encoding protein of *Rhipicephalus microplus* in southern Xinjiang. *China Animal Husbandry & Veterinary Medicine* **41**, 52−56 (2014).

299 Du, J. Y. et al. Morphological classification and molecular identification of *Rhipicephalus Turanicus* in Yining County. *Chin J Vector Biol & Control* **26**, 271−274 (2015).

300 He, H. J. & Huang, X. M. Investigation of Lyme spirochete on *Rhipicephalus microplus* in Southern Hunan. *Chin J Vector Biol & Control* **14**, 303 (2003).

301 Liu, X. M., Zhang, G. L., Zhao, Y., Sun, X. & Zheng, Z. Detection of pathogens of main tick-borne diseases in ticks from the desert area of Yuli, Xinjiang Uyghur Autonomous Region, China. *Chin J Vector Biol & Control* **23**, 14−16 (2012).

302 Sun, X., Zhang, G. L., Liu, X. M., Zhao, Y. & Zheng, Z. Investigation of tick species and tick-borne pathogens in Hoxud County of Xinjiang Uyghur Autonomous Region, China. *Chin J Vector Biol & Control* **24**, 5−7 (2013).

303 Li, H. M. et al. Detection and identification of *Ehrlichia* sp. in *Boophilus microplus* ticks from Xiamen of Fujian province. *Journal of Pathogen Biology* **1**, 174−176 (2006).

304 Hao, G. F. et al. Detection of tick and tick-borne pathogen in some ports of Inner Mongolia. *Chin J Epidemiol* **30**, 365−367 (2009).

305 Zhang, S. Z., Jiang, B., Wu, S. H. & Lin, L. Investigation and control of parasitic ticks in dogs in Fuzhou area. *Fujian Journal of Animal Husbandry and Veterinary Medicine* **35**, 11−13 (2013).

306 Chen, T. D., Zhao, Z. S. & Huang, L. Q. Biological characteristics of *Boophilus microplus* in Fujian. *Journal of Fujian Agriculture College* **18**, 548−552 (1989).

307 Zhang, B. L. *Rhipicephalus sanguineus* attacked domestic animals in suburban distcricts of Nanning. *Guangxi Agricultural Sciences* **21**, 48 (1984).

308 Chen, S. D., Wu, B. W., Chen, J. R., Lian, J. H. & Lin, D. N. Isolation of Q *Rickettsia* from *Boophilus Microplus* in Hainan Province. *Journal of Guangdong Medical College* **5**, 42−45 (1989).

309 Chen, J. R., Chen, S. D., Wu, B. W., Chen, L. H. & Lin, D. N. Preliminary investigation report of Q fever in Guangdong. *South China Journal of Preventive Medicine* **15**, 87−90 (1989).

310 Yi, C. Y., Guo, Q. Y., Xue, H., Peng, C. & Bayinchahn. Detection of pathogen DNA of *Babesia caballi* from *Rhipicephalus sanguineus* and *Dermacentor nuttalli*. *Heilongjiang Animal Science and Veterinary Medicine* **57**, 38−40 (2014).

311 Xu, H. B. et al. Two new records of ticks from Jiangxi Province (Acaridae: Ixodidae). *Chinese Journal of Vector Biology and Control* **27**, 496–497 (2016).

312 Zhang, Q. et al. Investigation and analysis of tick distribution and tick vector in Yunan district. *Modern Preventive Medicine* **42**, 3578–3580 (2015).

313 Zhan-ke, Q. *Investigation and Carry Ring Worm Taylor South of Gurbantunggut Desert Ticks*. Master Dissertation. Shihezi University, (2017).

314 Kolonin, G. V. Fauna of ixodid ticks of the world (Acari, Ixodidae). http://ixodidae.myspecies.info/biblio (2009).

315 Kang, Y. J. et al. Extensive diversity of Rickettsiales bacteria in two species of ticks from China and the evolution of the Rickettsiales. *BMC Evolutionary Biology* **14**, 167 (2014).

316 Jia, N. et al. Human infections with *Rickettsia raoultii*, China. *Emerg Infect Dis* **20**, 866−868 (2014).

317 Fan, M. Y. et al. Isolation of a spotted fever group rickettsia from a patient and related ecologic investigations in Xinjiang Uygur Autonomous Region of China. *J Clin Microbiol* **25**, 628−632 (1987).

318 Yu, X. et al. Genotypic and antigenic identification of two new strains of spotted fever group rickettsiae isolated from China. *J Clin Microbiol* **31**, 83−88 (1993).

319 Tian, Z. C. et al. First report on the occurrence of *Rickettsia slovaca* and *Rickettsia raoultii* in *Dermacentor silvarum* in China. *Parasit Vectors* **5**, 19 (2012).

320 Zhuang, L. et al. Discovery of Rickettsia species in *Dermacentor niveus Neumann* ticks by investigating the diversity of bacterial communities. *Ticks Tick Borne Dis* **5**, 564−568 (2014).

321 Pang, X. B. An ecological study of *Dermacentor silvarum* in Yangjiaping forest area of Xiaowutai Mountain, Hebei province. *Journal of Medical Pest Control* **20**, 423−424 (2004).

322 Sun, Y., Zheng, S. G. & Xu, R. M. Systematic classification and pictorial key of Dermacentor ticks (Ixodida: Ixodidae) in China. *Acta Parasitologica et Medica Entomologica Sinica* 25−40 (2017).

323 Tian, Q. Y. Two new records of ticks in Shanxi province. *Journal of Shanxi Medical University* **25**, 43 (1983).

324 Qiu, E. C., Jia, J. M., Dang, R. L. & Wu, X. A strain of roseomonas isolation from *dermacentor nuttalli* in Tacheng of Xinjiang, China. *Bulletin of Disease Control & Prevention* **26**, 32−34 (2011).

325 Sun, S. R., Hao, J. M., Luo, T., Wang, Q. G. & Zhang, Y. J. Analysis correlation between Xinjiang hemorrhagic fever prevalence and ecosystem diversity of its natural foci in the Tarim Rivers (II). *Bulletin of Disease Control & Prevention (China)* **29**, 7−9 (2014).

326 Zhang, W., Wang, Z., Wang, X., Shao, J. G. & Bayinchahn. Identification of ixodes on Yak surface and observation before and after bloodsucking. *Xinjiang Xumuye* **28**, 30−32 (2012).

327 Wang, Z. et al. Infection survey of the ticks on Yak of Mountain Grasslands in Xinjiang. *Xinjiang Agricultural Sciences* **48**, 1514−1519 (2011).

328 Liu, D. X. *Morphological study of two species of ticks in Qinghai province and epidemiology survey on the prevalence of tick-borne*, Qinghai University, (2014).

329 Ma, D. X. et al. Vector competence of *Dermacentor ewrestianus* in Rabbit Fever Foci in Ali area. *Endemic diseases bulletin* **12**, 60−61 (1997).

330 Sun, S., Burenminde., Lei, G., Chen, J. X. & Xu, B. C. Detection of *Yersinia Pestis* in *Dermacentor Nuttalli* in Xinjiang. *Bulletin of Disease Control & Prevention (China)* **18**, 93 (2003).

331 Ma, R. L. et al. Investigation on parasitic infection and prevention of sheep in Guinan County. *Animal Husbandry & Veterianry Medicine* **44**, 86−88 (2012).

332 Wu, C. Y. et al. *Coxiella burnetii* was first isolated from adult ticks collected in Mongolia province. *Chinese Journal of Zoonoses* **6**, 9−11 (1990).

333 Shi, Z. Y., Yang, Y. S., Li, Q. & Zhao, H. B. Study on species and geographic distribution of ticks in Gansu province. *Chinese Journal of Veterinary Science and Technology* **34**, 48−49 (2004).

334 Sun, X. et al. Molecular epidemiological study of *Rickettsia raoultii* in ticks from Xinjiang, China. *Chinese Journal of Epidemiology* **34**, 756−757 (2013).

335 Wei, X. Y., Ma, X. & Wu, H. Investigation and control of ixodes parasitism in dairy cattle. *Modern Animal Husbandry* **36**, 40 (2015).

336 Alimijiang, S. K. & Bayinchahn. Epidemiological investigation report on the Yak Theileriosis Annulata in the Yaxiang County. *Contemporary Animal Husbandry* **30**, 25−27 (2012).

337 Fan, D. H. et al. Investigation of population on ticks and it carried pathogens at Suifenhe and Dongning ports. *Science of travel medicine* **13**, 12−14 (2007).

338 Tuersun, S., Li, J., Wang, Z. B., Song, R. Q. & Bayinchahan. Detection of equine piroplasma and identification of vector ticks in Zhaosu County of Xinjiang. *Progress in Veterinary Medicine* **37**, 44−48 (2016).

339 Zhen, W. *The Identification and Evolutionary Analysis of Portion Dermacentor Species, and Serology Detected its Carrying Babesiosis in Xinjiang*. Master Dissertation. Xinjiang Agricultural University, (2014).

340 Zhang Wei, W. Z., Wang Xin, et al. Identification of Ixodes on Yak's Body Surface and Observation before and after Bloodsucking. *Xinjiang Animal Husbandry* **28**, 28–30 (2012).

341 Xia, H. et al. Epidemiological survey of Crimean-Congo hemorrhagic fever virus in Yunnan, China, 2008. *Int J Infect Dis* **15**, e459−463 (2011).

342 Sun, S. et al. Epidemiology and phylogenetic analysis of Crimean-Congo hemorrhagic fever viruses in Xinjiang, China. *J Clin Microbiol* **47**, 2536−2543 (2009).

343 Li, Y. et al. Experimental transmission of *Theileria ovis* by *Hyalomma anatolicum anatolicum*. *Parasitol Res* **106**, 991−994 (2010).

344 Li, W., Sun, Y., Zhang, G. L. & Xu, R. M. *Genus Hyalomma Koch*, 1844 (Ixodoidea, Ixodidae) in China with description of new record species *Hyalomma excavatum Koch*, 1844. *Acta Parasitol Med Entomol Sin* **22**, 94−103 (2015).

345 Guo, B. Y. & He, Z. T. Diagnosis and control measures of bovine theileriasis. *Jilin Animal Husbandry and Veterinary Medicine* **30**, 38−39 (2009).

346 Li, Y. P. & Zhou, X. P. Molecular epidemiological study on natural infection caused by 3 ticks in northwestern China. *Int J Lab Med* **33**, 52−53 (2012).

347 Cao, W. L., Li, L., Wang, B. J., Shi, J. C. & Bayinchahan. Observations on the endoparasites infection in cattle/yaks and living-habits of *Hyalomma Asiaticum Kozlovi* in Hejing County. *Xinjiang Agricultural Sciences* **49**, 186−191 (2012).

348 Kong, Z. M. & Zhou, X. R. Sero-epidemiological investigation and pathogen isolation of Q fever in Xinjiang population. *Medical Journal of National Defending Forces in Northwest China* **12**, 43−44 (1991).

349 Abulikemu, A., Li, B., Jiang, W. & Yu, X. Report on the first discovery of *Hyalomma anatolicum* in Turpan. *Endemic diseases bulletin* **21**, 10 (2006).

350 Kong, Z. M. et al. Investigation on ticks and tick-borne natural focal infections in Xinjiang. *Endemic diseases bulletin* **2**, 1−4 (1987).

351 Yang, Y. et al. Investigation on tick-borne pathogens in Ganqimaodu Port areas on the borders between China and Mongolia, 2012−2013. *Chinese Journal of Frontier Health and Quarantine* **39**, 330−332 (2016).

352 Yang Y et al. Investigation on tick-borne pathogens at Ganqimaodu Port between Sino-Mongolia borders. *Chinese Journal of Frontier Health and Quarantine* **34**, 339−342 (2011).

353 Xun, H. T., Xue, H. W., Yin, H., Sun, X. L. & Luo, J. X. Phylogenetic study of *Hyalomma* spp. from China based on ITS and COI genes. *Chinses Veterinary Science* **46**, 563−567 (2016).

354 Wang, B. J., Zhu, Y. T., Liu, M. L., Song, R. Q. & Bayinchahan. Identification of *Hyalomma anatolicum* and test of its carried pathogen DNA of *Theileria annulata* in cattle in Xinjiang. *Chin J Vet Sci* **35**, 1939−1942 (2015).

355 Dai, X. et al. Geography and host distribution of Crimean-Congo hemorrhagic fever in the Tarim basin. *Chin J Epidemiol* **27**, 1048−1052 (2006).

356 Liu, A. H. et al. Isolation and identification of the piroplasma species transmitted by *Hyalomma scupense* and preliminary study on its biological characteristics. *Progress in Veterinary Medicine* **30**, 17−20 (2009).

357 Hainimuguli, A., Yi, C. Y., Mireguli, T. & Bayinchahan. Morphological identification of vector ticks parasitic in cattle body surface in part of Toksun County. *Modern Agricultural Science and Technology* **44**, 252−254 (2015).

358 Song, S. R., Li, L. R., Shi, X. & Fu, Y. M. Investigation report on Bovine Ring Taylor Coccidiosis in northern provinces (areas) of China. *Guizhou Journal of Animal Husbandry & Veterinary Medicine* **24**, 9−10 (2000).

359 Feng, C. H. et al. A survey on natural foci of Xinjiang hemorrhagic fever in Bachu County of Xinjiang. *Endemic diseases bulletin* **3**, 66−69 (1988).

360 Teng, K. F. & Guang, C. A. A new species of tick of the Genus *Anomalohimalaya* from China (Acarina: Ixodidae). *Acta Entomologica Sinica* **24**, 99−102 (1981).

361 Zhang, S. J., Zhang, G. Z. & Zhang, J. C. Biological characteristics and control of *Ornithodoros lahorensis* in Minghua District of Sunan County. *Gansu Animal Husbandry and Veterinary* **14**, 9−15 (1983).

362 Pang, C. J., Shao, G. N. & Zhu, J. Z. Investigation of tick-borne retrograde fever vectors. *People's Military Surgeon* **11**, 47−52 (1960).

363 Nur, K. et al. Investigation and identification of ticks from sheep in Shanshan, Xinjiang. *Grass-Feeding Livestock* **36**, 51−53 (2015).

364 Zhang, Z., Yang, J., Zhang, G. Z. & Zhang, Z. C. Investigation of *Ornithodoros lahorensis* in Minghua District, Sunan County, Gansu Province. *Chinese Veterinary Science* **11**, 27−29 (1981).

365 Qiu, M. H. & Zhu, C. J. A new species of bat ticks from China (Ixodoidea: Argasidae). *Acta Entomologica Sinica* **25**, 328−331 (1982).

366 Miao, F. & Li, W. Q. A case of Persian acute tick disease. *Chin J Parasit Dis Con* **16**, 27 (2003).

367 Qin, Z. H., Li, Z. P. & Zhou, H. F. Persian sharp tick was found in the hilly area along Yanhuai in Anhui Province. *Acta Arachnologica Sinica* **5**, 53 (1996).

368 Wang, S., Lv, W. M., Yang, Z. & Huang, S. X. Investigation and control of chicken Persian sharp-edged ticks. *Poultry Husbandry and Disease Control* **4**, 33−34 (1985).

369 Teng, K. F. Note on Chinese ticks of the subgenus Argas (Acarina: Argasidae: Argas). *Acta Zootaxonomica Sinica* **8**, 255−261 (1983).

370 Teng, K. F. & Song, J. Y. A new species of Argas from Jiangxi, China (Acarina: Argasidae). *Acta Zootaxonomica Sinica* **8**, 153−156 (1983).
