# Supplementary material for: Mapping ticks and tick-borne pathogens in China
Source: Nat Commun. 2021 Feb 17;12:1075. doi: 10.1038/s41467-021-21375-1 (PMC7889899; doi:10.1038/s41467-021-21375-1)
Supplement: Supplementary file 3 — Description of Additional Supplementary Files [file 41467_2021_21375_MOESM3_ESM.pdf]

Description of Additional Supplementary Files

File Name: Supplementary Data 1
Description: Frequency and vegetation coverage characteristics of counties with detection of each tick species in China. Names of nineteen tick species that were recorded in more than 40 counties are bolded.

File Name: Supplementary Data 2
Description: Tick species detected during 1950 –2018 in seven zoogeographic regions of China: Northeast District (NE), North China District (N), Inner MongoliaXinjiang District (IMX), Qinghai-Tibet District (QT), Southwest District (SW), Central China District (C) and South China District (S). These regions are shown in Fig. 1 in the main text and numbered I –VII.

File Name: Supplementary Code 1
Description: R language code used to build BRT model, filter main variables and generate prediction results in this study.
