# Supplementary material for: Mapping ticks and tick-borne pathogens in China
Source: Nat Commun. 2021 Feb 17;12:1075. doi: 10.1038/s41467-021-21375-1 (PMC7889899; doi:10.1038/s41467-021-21375-1)
Supplement: Supplementary file 7 — Reporting Summary [file 41467_2021_21375_MOESM7_ESM.pdf]

nature research

Corresponding author(s): Li-Qun Fang
Last updated by author(s): Jan 9, 2021

Reporting Summary

Nature Research wishes to improve the reproducibility of the work that we publish. This form provides structure for consistency and transparency in reporting. For further information on Nature Research policies, see our Editorial Policies and the Editorial Policy Checklist.

Statistics

For all statistical analyses, confirm that the following items are present in the figure legend, table legend, main text, or Methods section.

n/a	Confirmed	
☒	☐	The exact sample size (*n*) for each experimental group/condition, given as a discrete number and unit of measurement
☒	☐	A statement on whether measurements were taken from distinct samples or whether the same sample was measured repeatedly
☐	☒	The statistical test(s) used AND whether they are one- or two-sided<br>*Only common tests should be described solely by name; describe more complex techniques in the Methods section.*
☐	☒	A description of all covariates tested
☐	☒	A description of any assumptions or corrections, such as tests of normality and adjustment for multiple comparisons
☐	☒	A full description of the statistical parameters including central tendency (e.g. means) or other basic estimates (e.g. regression coefficient) AND variation (e.g. standard deviation) or associated estimates of uncertainty (e.g. confidence intervals)
☐	☒	For null hypothesis testing, the test statistic (e.g. *F*, *t*, *r*) with confidence intervals, effect sizes, degrees of freedom and *P* value noted<br>*Give P values as exact values whenever suitable.*
☒	☐	For Bayesian analysis, information on the choice of priors and Markov chain Monte Carlo settings
☐	☒	For hierarchical and complex designs, identification of the appropriate level for tests and full reporting of outcomes
☐	☒	Estimates of effect sizes (e.g. Cohen's *d*, Pearson's *r*), indicating how they were calculated

Our web collection on statistics for biologists contains articles on many of the points above.

Software and code

Policy information about availability of computer code

Data collection	No software was used in the data collection.
Data analysis	ArcGIS 10.0 software (ESRI Inc., Redlands, CA, USA), the statistical platform R v3.6.0 (R Core Team, 2019), ROCR v1.0-7, pROC v1.15.0, R dismo v1.1-4, and gbm v2.1.5 packages were used in the data analysis.

For manuscripts utilizing custom algorithms or software that are central to the research but not yet described in published literature, software must be made available to editors and reviewers. We strongly encourage code deposition in a community repository (e.g. GitHub). See the Nature Research guidelines for submitting code & software for further information.

Data

Policy information about availability of data

All manuscripts must include a data availability statement. This statement should provide the following information, where applicable:
- Accession codes, unique identifiers, or web links for publicly available datasets
- A list of figures that have associated raw data
- A description of any restrictions on data availability

Model results generated as part of this study are available. The raw data that support the findings of this study are publicly available. The source data underlying Figs.1, 2, 3, 4, and 5, Supplementary Figs. 1-10, and Supplementary Figs. 30-41 are provided as Source Data file. Source data are provided with this paper.

Field-specific reporting

Please select the one below that is the best fit for your research. If you are not sure, read the appropriate sections before making your selection.

☐ Life sciences ☐ Behavioural & social sciences ☒ Ecological, evolutionary & environmental sciences

For a reference copy of the document with all sections, see nature.com/documents/nr-reporting-summary-flat.pdf

Ecological, evolutionary & environmental sciences study design

All studies must disclose on these points even when the disclosure is negative.

Study description	A comprehensive database that comprised all 124 tick species, 103 tick-borne agents detected from ticks, as well as all human cases infected with 23 species (subspecies) of tick-borne bacteria or 6 tick-borne viruses, which were reported during 1950−2018 in China were assembled, and their spatial distributions were mapped. Impacts of eco-climatic and environmental factors on their distributions were assessed for 19 vector ticks and 2 tick-borne pathogens with the main disease burden using a machine learning algorithm, and the model-predicted risk maps were created.
Research sample	An integrated database comprising 7,344 unique records on geographic distributions of 124 known tick species, including 113 hard tick species in seven genera and 11 soft tick species in two genera, together with 103 tick-associated agents detected in either ticks or humans, which were recorded in 1,134 counties (39% of all counties in China), and no existing datasets on the distributions of ticks and tick-associated agents were involved in this study.
Sampling strategy	There are no sampling procedure in this study.
Data collection	We assembled a comprehensive database of ticks and tick-borne pathogens (the database is available upon request) by combining data from a variety of sources, including (1) literatures reporting the occurrence of 124 ticks and 103 tick-associated agents in China, published between Jan, 1950 and Dec, 2018, (2) historical data (before 1990) on presence records of ticks across China that are not formally published but available in the Medical Entomology Gallery (MEG) and unpublished data on the prevalence of ticks or tick-borne pathogens from entomological surveys conducted by our institute in mainland China from 1990 to 201839, and (3) newly conducted field surveys of tick species across the country. All the entomological surveys in literature and conducted by our institute were cross-sectional studies. For the literature review, five main electronic databases (PubMed and ISI Web of Science, China WanFang database, China National Knowledge Infrastructure, and Chinese Scientific Journal Database) were searched for studies published between Jan, 1950 and December, 2018, using the following keywords: ("Tick" or "Ticks") and "China". We also checked the references in retrieved articles to reach more relevant articles. Each article was carefully reviewed by two team members independently to collect the following information using a standard form: study date, study location, spatial resolution, tick species identified, laboratory methods, and detection results for tick-borne pathogens. Any disagreement between the two staff members was resolved by discussion and consensus among the reviewers and other co-authors. Only studies with clearly identifiable results, i.e., presence or absence, time and location of tick species or tick-borne pathogens were included in our database.
Timing and spatial scale	Our analyses were performed at the county level, and the risk distributions of 19 vector ticks and 2 tick-borne pathogens were assessed by using cross-section data during 1950-2018 and 2005-2018, respectively. To our knowledge, it is the study with the highest spatial resolution on the systematical investigation of ecological niches of major tick species or prevalent tick-borne pathogens in China up to now.
Data exclusions	No data were excluded in the analyses.
Reproducibility	The analyses were performed independently four times in the last year, and all attempts to repeat the analyses were successful.
Randomization	A training set with 75% of data points was randomly selected by bootstrapping without replacement (i.e., bootstrapping the 1,134 counties with survey of ticks), the remaining 25% serving as a test set. A BRT model was built using the training set, and then applied to the test set for validation if needed.
Blinding	No blinding was used in the data analyses, which was not needed for the BRT models.

Did the study involve field work? ☐ Yes ☒ No

Reporting for specific materials, systems and methods

We require information from authors about some types of materials, experimental systems and methods used in many studies. Here, indicate whether each material, system or method listed is relevant to your study. If you are not sure if a list item applies to your research, read the appropriate section before selecting a response.

Materials & experimental systems

n/a	Involved in the study
☒	☐ Antibodies
☒	☐ Eukaryotic cell lines
☒	☐ Palaeontology and archaeology
☒	☐ Animals and other organisms
☒	☐ Human research participants
☒	☐ Clinical data
☒	☐ Dual use research of concern

Methods

n/a	Involved in the study
☒	☐ ChIP-seq
☒	☐ Flow cytometry
☒	☐ MRI-based neuroimaging
